# PLETHORA‐WOX5 interaction and subnuclear localization control *Arabidopsis* root stem cell maintenance

**DOI:** 10.15252/embr.202154105

**Published:** 2022-04-04

**Authors:** Rebecca C Burkart, Vivien I Strotmann, Gwendolyn K Kirschner, Abdullah Akinci, Laura Czempik, Anika Dolata, Alexis Maizel, Stefanie Weidtkamp‐Peters, Yvonne Stahl

**Affiliations:** ^1^ Institute for Developmental Genetics Heinrich‐Heine University Düsseldorf Germany; ^2^ Center for Organismal Studies (COS) University of Heidelberg Heidelberg Germany; ^3^ Center for Advanced Imaging Heinrich‐Heine University Düsseldorf Germany; ^4^ Present address: Biological and Environmental Sciences and Engineering (BESE) King Abdullah University of Science and Technology (KAUST) Thuwal Saudi Arabia; ^5^ Present address: Molecular Plant Science/Plant Biochemistry University of Wuppertal Wuppertal Germany

**Keywords:** differentiation, nuclear bodies, prion‐like domains, root stem cells, transcription factor complexes, Chromatin, Transcription & Genomics, Plant Biology, Stem Cells & Regenerative Medicine

## Abstract

Maintenance and homeostasis of the stem cell niche (SCN) in the *Arabidopsis* root is essential for growth and development of all root cell types. The SCN is organized around a quiescent center (QC) maintaining the stemness of cells in direct contact. The key transcription factors (TFs) WUSCHEL‐RELATED HOMEOBOX 5 (WOX5) and PLETHORAs (PLTs) are expressed in the SCN where they maintain the QC and regulate distal columella stem cell (CSC) fate. Here, we describe the concerted mutual regulation of the key TFs WOX5 and PLTs on a transcriptional and protein interaction level. Additionally, by applying a novel SCN staining method, we demonstrate that both WOX5 and PLTs regulate root SCN homeostasis as they control QC quiescence and CSC fate interdependently. Moreover, we uncover that some PLTs, especially PLT3, contain intrinsically disordered prion‐like domains (PrDs) that are necessary for complex formation with WOX5 and its recruitment to subnuclear microdomains/nuclear bodies (NBs) in the CSCs. We propose that this partitioning of PLT‐WOX5 complexes to NBs, possibly by phase separation, is important for CSC fate determination.

## Graphical abstract

Stem cell maintenance in the *Arabidopsis* root is controlled by transcription factor complexes containing PLETHORAs and WOX5. Their localization in nuclear bodies (NBs), dependent on the differentiation status, controls cell fate determination.

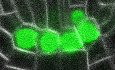

## Introduction

**Figure 1:**
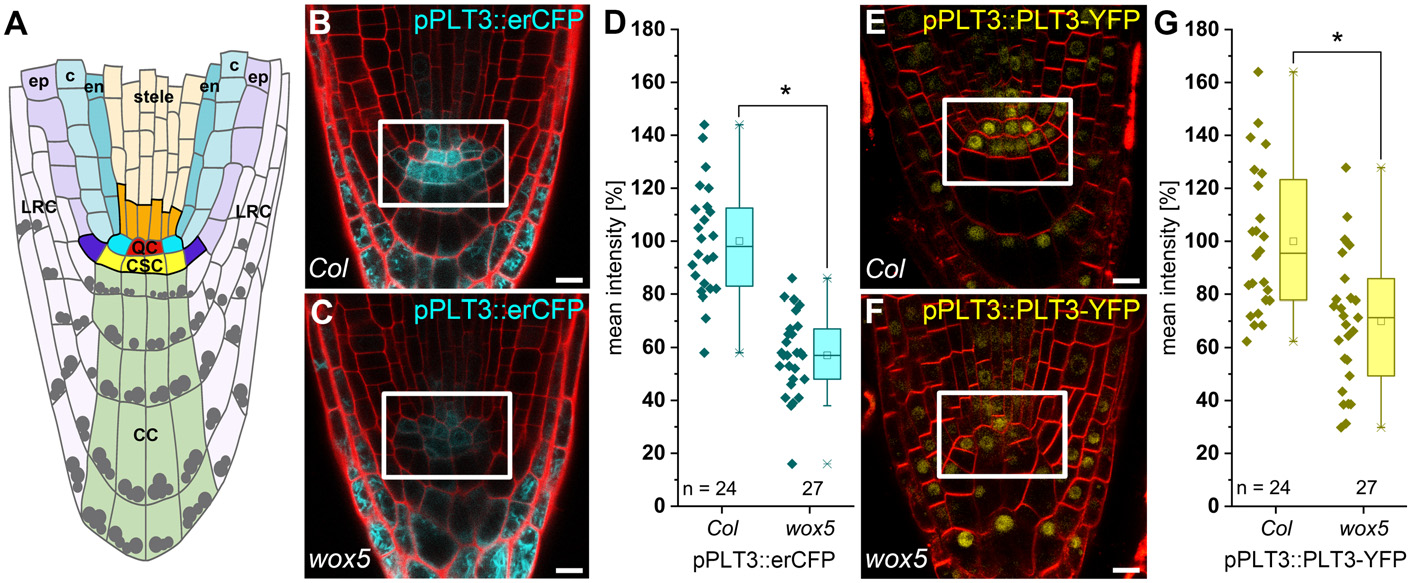
WOX5 positively regulates *PLT3* expression ASchematic representation of the *Arabidopsis* root meristem. The QC cells (red) maintain the surrounding stem cells (initials) outlined in black together building the root stem cell niche (SCN). The different cell types are color coded. QC = quiescent center (red); CSC = columella stem cells (yellow); CC = columella cells (green); LRC = lateral root cap (light purple); ep = epidermis (purple); c = cortex (light turquoise); en = endodermis (dark turquoise); bright turquoise = cortex/endodermis initials; dark purple = epidermis/lateral root cap initials; dark orange = stele initials; stele = light orange; grey dots = starch granules.B, CRepresentative images of pPLT3::erCFP (cyan) expressing and PI‐stained (red) *Arabidopsis* roots in *Col* or *wox5* background, respectively.DMean fluorescence intensities of the pPLT3::erCFP roots summarized in box and scatter plots. The mean fluorescence intensity of the CFP signal in *Col* roots was to set to 100%.E, FRepresentative images of pPLT3::PLT3‐YFP (yellow) expressing and FM4‐64‐stained (red) *Arabidopsis* roots in *Col* or *wox5* mutant background, respectively.GMean fluorescence intensities of the pPLT3::PLT3‐YFP expressing roots summarized in box and scatter plots. The mean fluorescence intensity of the YFP signal in *Col* roots was to set to 100%. Data information: (D, G) Box = 25–75% of percentile, whisker = 1.5 interquartile range, − = median, □ = mean value, X = minimum/maximum. The data were statistically analyzed by one‐way ANOVA and Holm–Sidak *post‐hoc* multiple comparisons test. Asterisks indicate statistically significant differences (α = 0.01). Number of analyzed roots (*n*) (biological replicates) is indicated for each genotype and results from two technical replicates. (B, C, E, F) Scale bars represent 10 µm. SCN = stem cell niche; PI = propidium iodide; YFP = yellow fluorescent protein; CFP = cyan fluorescent protein.

The root system of higher plants is essential for plant life, as it provides anchorage in the soil and access to nutrients and water. It arises from a population of long‐lasting stem cells residing in a structure called root apical meristem (RAM) at the tip of the root. Within the *Arabidopsis thaliana* RAM, the stem cell niche (SCN) consists of on average four to eight slowly dividing cells, the QC cells, which act as a long‐term reservoir and signaling center by maintaining the surrounding shorter‐lived, proliferating stem cells (also called initials) in a non‐cell autonomous manner (van den Berg *et al*, 1997; Lu *et al*, 2021). These stem cells continuously divide asymmetrically, thereby generating new stem cells that are still in contact with the QC. The hereby‐produced daughter cells frequently undergo cell divisions and are shifted further away from the QC to finally differentiate into distinct cell fates. By this mechanism, the position of the stem cells in the root remains the same throughout development and their precise orientation of division leads to the formation of concentrically organized clonal cell lineages representing a spatio‐temporal developmental gradient (Dolan *et al*, 1993; van den Berg *et al*, 1997; Benfey & Scheres, 2000). From the inside to the outside, the following root cell tissues develop: vasculature, pericycle, endodermis, cortex, and epidermis plus columella and lateral root cap at the distal root tip (Fig 1A).

The necessary longevity and continuous activity of the RAM can only be achieved if its stem cell pool is constantly replenished, since cells are frequently leaving the meristematic region due to continuous cell divisions. Therefore, complex regulatory mechanisms involving phytohormones and key TFs regulate stem cell maintenance and the necessary supply of differentiating descendants (Drisch & Stahl, 2015). Here, the APETALA2‐type PLT TF family and the homeodomain TF WOX5 play important roles (Aida *et al*, 2004; Sarkar *et al*, 2007). WOX5 is expressed mainly in the QC, but maintains the surrounding stem cells non‐cell‐autonomously by repressing their differentiation (Sarkar *et al*, 2007; Pi *et al*, 2015). Loss of WOX5 causes the differentiation of the CSCs, also called distal stem cells, into starch‐accumulating columella cells (CCs), while increased WOX5 expression causes CSC over‐proliferation. Hence, WOX5 abundance is critical and necessary to suppress premature CSC differentiation (Sarkar *et al*, 2007; Pi *et al*, 2015). WOX5 also represses QC divisions, maintaining the quiescence of the QC by repressing CYCLIN D (CYCD) activity within the QC (Forzani *et al*, 2014).

The auxin‐induced PLTs form a clade of six TFs and act as master regulators of root development, as multiple *plt* mutants fail to develop functional RAMs (Aida *et al*, 2004; Galinha *et al*, 2007; Mähönen *et al*, 2014). PLT1, 2, 3, and 4 are expressed mainly in and around the QC and form an instructive gradient, which is required for maintaining the balance of stem cell fate and differentiation. This PLT gradient is also necessary for separating auxin responses in the SCN, for the correct positioning of the QC, and the expression of QC markers (Aida *et al*, 2004; Galinha *et al*, 2007; Mähönen *et al*, 2014). Genetically, WOX5 and PLT1 were shown to play an interconnected role in auxin‐regulated CSC fate, whereas PLT1 and PLT3 were found to directly positively regulate WOX5 expression (Ding & Friml, 2010; Shimotohno *et al*, 2018).

Although PLTs and WOX5 are known for controlling stem cell regulation and maintenance in the *Arabidopsis* RAM and genetic evidence for cross regulation exists, the underlying molecular mechanisms are until now largely elusive. Here, we show for the first time that the mutual regulation of expression, but importantly also the ability of PLTs to directly interact with and recruit WOX5 to NBs in CSCs controls stem cell homeostasis in the *Arabidopsis* RAM. NBs are membrane‐less, self‐assembling protein/RNA containing compartments thought to regulate a variety of physiological responses to differential environmental cues like light, temperature, or osmotic changes (Mao *et al*, 2011; Jung *et al*, 2020; Meyer, 2020). Therefore, we propose a model in which differential PLT/WOX5 complexes depending on their subnuclear localization in NBs or in the nucleoplasm regulate stem cell fate in the RAM, possibly by phase separation.

## Results

### WOX and PLTs regulate each other’s expression in the root SCN

WOX5 and PLTs are essential players in distal stem cell maintenance (Aida *et al*, 2004; Galinha *et al*, 2007; Sarkar *et al*, 2007; Pi *et al*, 2015). This, as well as their overlapping expression and protein localization domains in the root SCN raised the question if they could act together in distal stem cell regulation, where, in comparison to all the other PLTs, particularly PLT3 is highly expressed (Fig 1B) (Galinha *et al*, 2007). Furthermore, PLT3 was recently predicted as one of the central nodes regulating other QC‐enriched TFs in the underlying gene regulatory network (GRN) within the *Arabidopsis* root SCN. In contrast, PLT1 and PLT2 were predicted as minor nodes only and PLT4 (BBM) was not predicted as a node (de Luis Balaguer *et al*, 2017).

**Figure 2:**
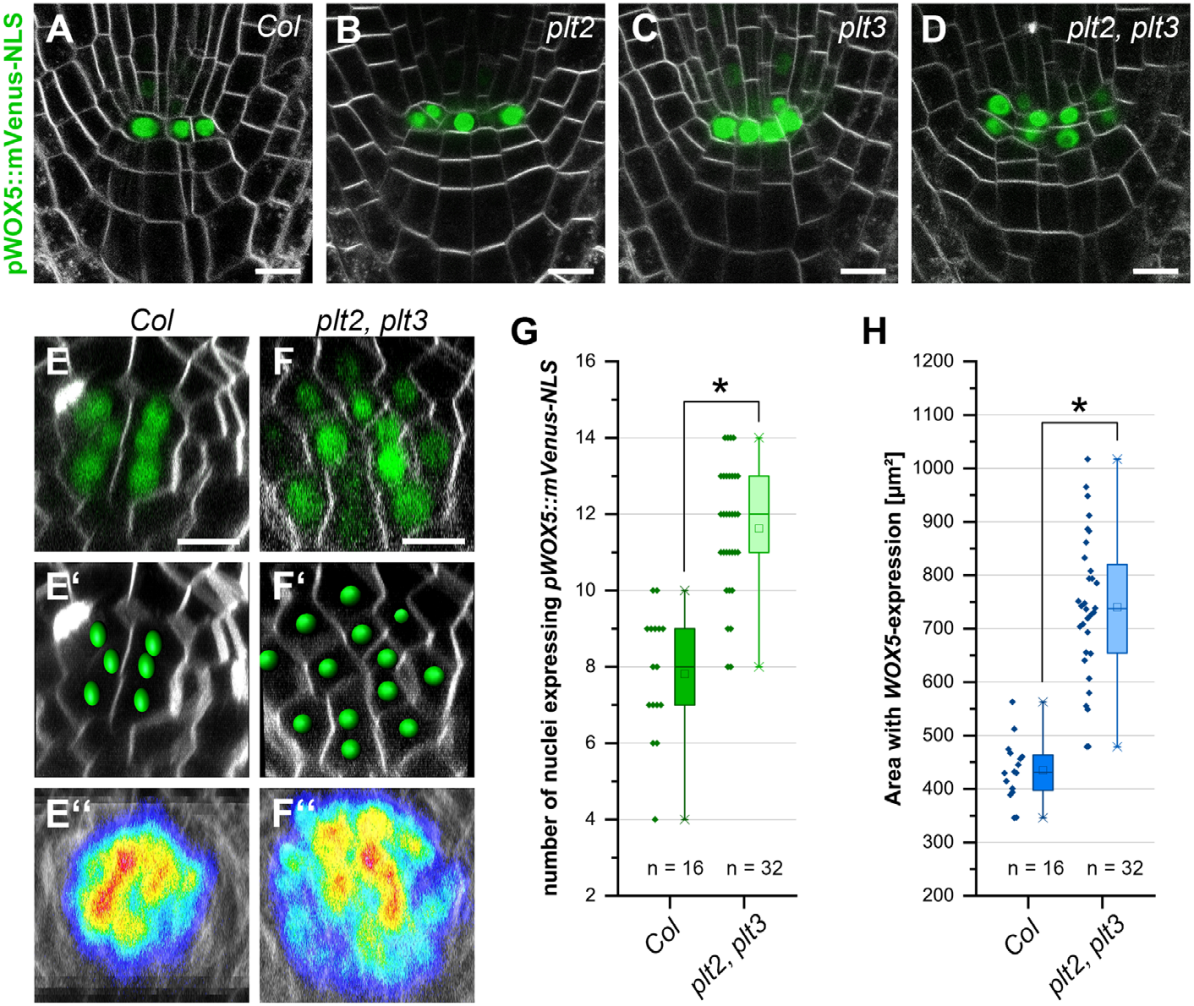
PLTs constrain the *WOX5* expression domain A–FRepresentative FM4‐64‐stained *Arabidopsis* roots (grey) expressing pWOX5::mVenus‐NLS (green) in *Col*, *plt2*, *plt3,* and *plt2, plt3* double mutant background in longitudinal (A‐D), or transversal (E‐F) optical sections. (E′, F′) Analysis of representative images in (E) and (F) in Imaris to detect and count individual expressing nuclei. (E″, F″) Overlay of 10 roots (biological replicates) showing the area of detected fluorescence (high levels in red, low levels in blue) in *Col* and *plt2, plt3* double mutant roots.GNumber of nuclei (biological replicates) expressing pWOX5::mVenus‐NLS in *Col* and *plt2, plt3* double mutant roots summarized in box and scatter plots.HArea of WOX5 expression in µm^2^ in *Col* and *plt2, plt3* double mutant roots summarized in box and scatter plots. Data information: (G, H) Box = 25‐75% of percentile, whisker = 1.5 interquartile range, − = median, □ = mean value, X = minimum/maximum. (G, H) Kruskal–Wallis ANOVA with subsequent Dunn’s test (G) or one‐way ANOVA and *post‐hoc* Holm–Sidak multiple comparisons test was used to test for statistical significance (H). Asterisks indicate statistically significant differences (α = 0.01). Number of analyzed roots (*n*) (biological replicates) is indicated for each genotype and results from three technical replicates per genotype. Scale bars represent 10 µm; NLS = nuclear localization signal.

First, we tested if WOX5 influences *PLT3* expression. Both a transcriptional and translational PLT3 fluorescent reporter line showed a reduced expression in the QC and CSC in a *wox5* mutant background to around 57–70% compared to the *Col‐0* (*Col*) wild‐type roots (Fig 1B–G, Appendix Table S3). Next, we addressed, if *PLT3* expression is regulated directly or indirectly upon WOX5 induction by using the published *Arabidopsis* lines 35S::WOX5‐GR (Sarkar *et al*, 2007) and 35S::WOX5‐GFP‐GR (Berckmans *et al*, 2020) in quantitative PCR experiments (qPCR) (Appendix Fig S1A, Appendix Table S1) and crosses with pPLT3::erCFP (Galinha *et al*, 2007) (Appendix Fig S1B–E, Appendix Table S2), respectively. In both independent experiments, we found no change of *PLT3* expression 4 h after WOX5 induction. After 21 h of WOX5 induction, we found *PLT3* expression significantly upregulated up to two‐fold and therefore, we conclude that *PLT3* expression is not directly regulated by WOX5 (Appendix Fig S1B–E, Appendix Table S2). This extends the previously reported regulation of *PLT1* expression by WOX5 (Ding & Friml, 2010) and shows that WOX5 positively regulates expression of several *PLTs*, albeit in an indirect manner. To test if *WOX5* expression also depends on PLTs, we produced a transcriptional reporter, which expresses a nuclear‐localized mVenus under control of the *WOX5* promoter. In agreement with previous reports, expression of *WOX5* in our transcriptional reporter line is confined to the QC and is only weakly expressed in the stele initials (Sarkar *et al*, 2007; Pi *et al*, 2015) (Fig 2A).

PLTs are known for their redundant function in SCN maintenance, that can be very strong especially when PLT1 is mutated in combination with other PLTs (Aida *et al*, 2004; Galinha *et al*, 2007). Because we aimed to look at the rather subtle QC and distal SCN phenotypes, we therefore included only *plt2* mutants for our analyses. In *plt2* and *plt3* single mutants, we observed additional mVenus‐expressing cells in the QC region, which may derive from aberrant periclinal cell divisions of the QC (Fig 2B and C, Appendix Table S4). This effect is even stronger in the *plt2, plt3* double mutant roots, where extra cells are found in all observed roots and often even form an additional cell layer of *WOX5* expressing cells (Fig 2D).

Previously, it was reported that the *Arabidopsis* wild‐type QC is composed of four to eight cells with a low division rate (Truernit *et al*, 2008; Cruz‐Ramírez *et al*, 2013; Stahl *et al*, 2013; Lu *et al*, 2021). We quantified the number of *WOX5* expressing cells and the area of *WOX5* expression per root by acquiring transverse optical sections through the roots. We observed four to ten *WOX5* expressing cells in the *Col* wild type (Fig 2E and G, Appendix Table S4), whereas we found eight to 14 *WOX5* expressing cells and a laterally expanded *WOX5* expression domain in the *plt2, plt3* double mutants (Fig 2F–H, Appendix Table S4). Taken together, our data show that WOX5 positively regulates *PLT* expression, here shown for PLT3, whereas PLT2 and PLT3 redundantly restrict *WOX5* expression to a limited number of cells at QC position, possibly by negative feedback regulation. These observations are in agreement with a previous report, where a role for PLT1 and PLT2 in confining *WOX5* expression was reported (Sarkar *et al*, 2007).

### A novel SCN staining method for simultaneous QC division and CSC differentiation analyses

**Figure 3:**
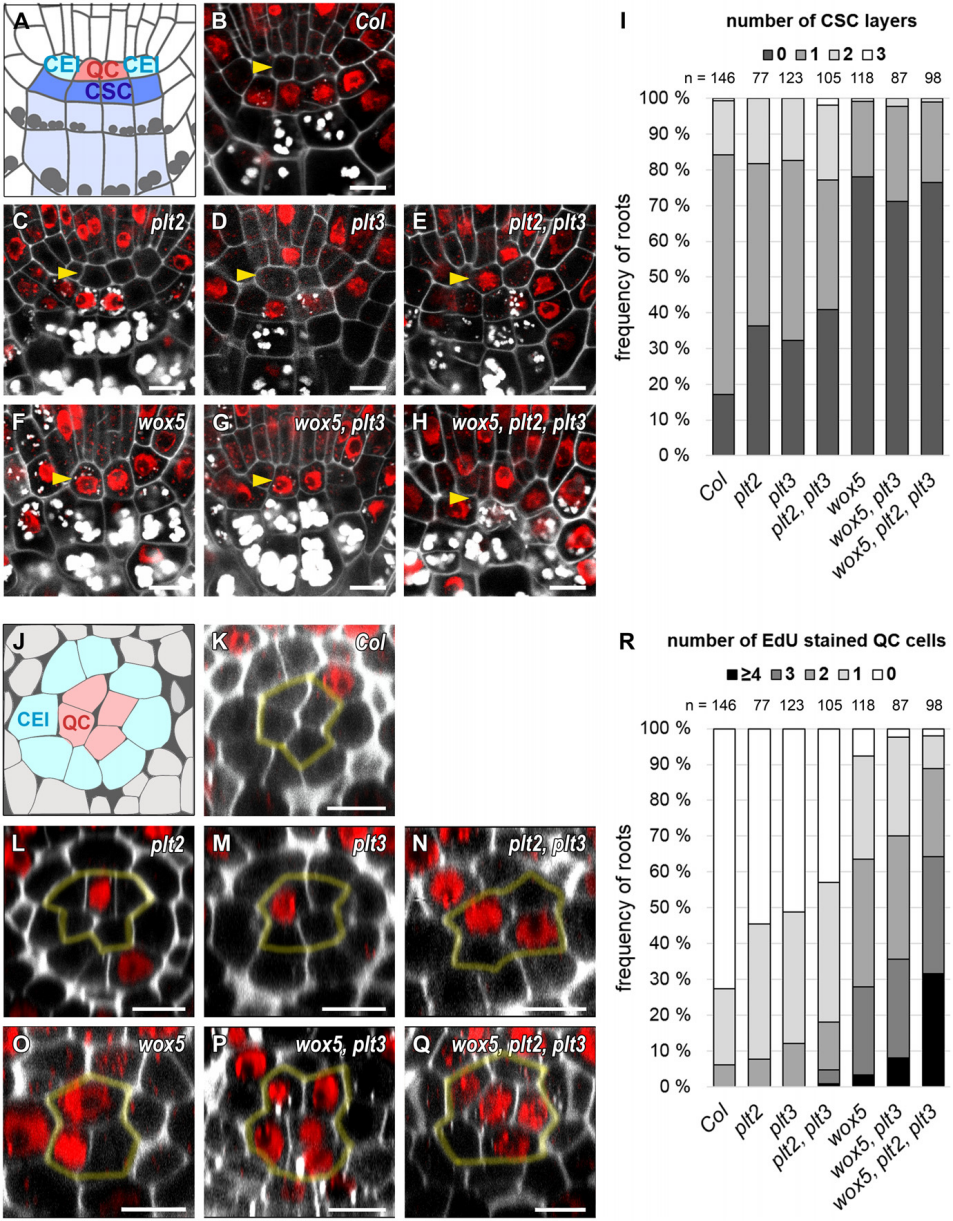
*plt* and *wox5* mutants show more CSC differentiation and QC divisions ASchematic representation of a longitudinal section of an *Arabidopsis* RM. QC cells are marked in red, CSCs are marked in dark blue, CCs in light blue. Combined mPSPI (grey) and EdU (red) staining for 24 h (SCN staining) to analyze the CSC (A‐I) and QC division phenotype (J‐R) within the same roots are shown.B–HRepresentative images of the SCN staining in *Col*, and the indicated single, double, and triple mutant roots. QC positions are marked by yellow arrowheads.IAnalyses of the SCN staining for CSC phenotypes. Frequencies of roots showing 0, 1, 2, or 3 CSC layers are plotted as bar graphs.JSchematic representation of a transversal section of an *Arabidopsis* RM. QC cells are marked in red, CEI initials are marked in turquoise.K–QRepresentative images of transversal sections with QC cells outlined in yellow.RAnalyses of the SCN staining for QC division phenotypes. Frequencies of roots showing 0, 1, 2, 3 or ≥ 4 dividing QC cells are plotted as bar graphs. Data information: Number of analyzed roots (*n*) (biological replicates) is indicated for each genotype and results from 2‐5 technical replicates per genotype. QC = quiescent center, CSC = columella stem cell, CEI = cortex endodermis initial, SCN = stem cell niche, mPSPI = modified pseudo‐Schiff propidium iodide, EdU = 5‐ethynyl‐2′‐deoxyuridine, scale bars represent 5 µm. Source data are available online for this figure.

QC cells rarely divide as they provide a long‐term reservoir to maintain the surrounding stem cells (Cruz‐Ramírez *et al*, 2013; Vilarrasa‐Blasi *et al*, 2014). As WOX5 and PLTs control QC cell divisions and CSC maintenance (Aida *et al*, 2004; Galinha *et al*, 2007; Sarkar *et al*, 2007; Forzani *et al*, 2014; Mähönen *et al*, 2014; Pi *et al*, 2015), we asked if these two aspects are interdependent. Therefore, we analyzed the cell division rates in the QC and the CSC phenotypes in wild‐type and mutant roots. To assess these two phenotypes and to probe for their interdependency, we needed to measure the number of dividing QC cells and CSC layers within the same root simultaneously. To enable this, we established a novel staining method, named SCN staining, by combining the 5‐ethynyl‐2′‐deoxyuridine (EdU) and modified pseudo Schiff base propidium iodide (mPS‐PI) stainings to simultaneously visualize cell divisions, starch granule distribution as well as cell walls within the same root (Truernit *et al*, 2008; Schiessl *et al*, 2012 §; Cruz‐Ramírez *et al*, 2013). Applying this new staining combination, potential correlations between QC‐divisions and CSC cell fates can be uncovered. The EdU‐staining is used to analyze QC‐divisions by staining nuclei that have gone through the S‐phase, detecting cells directly before, during, and after cell division (Cruz‐Ramírez *et al*, 2013). However, cell layers and different cell types are hard to distinguish using only EdU staining due to the lack of cell wall staining. Therefore, we additionally applied the mPS‐PI‐method to stain cell walls and starch which is commonly used for CC and CSC cell fate determination (Truernit *et al*, 2008; Stahl *et al*, 2009, 2013). CCs are differentiated, starch granule‐containing cells in the distal part of the root mediating gravity perception. They derive from the CSCs that form one or, directly after cell division, two cell layers distal to the QC. The CSCs lack big starch granules and can thereby easily be distinguished from the differentiated CCs by mPS‐PI staining (Truernit *et al*, 2008; Stahl *et al*, 2009, 2013) (see Fig 3A, B and I, Appendix Table S5).

### QC division rate and CSC differentiation correlate in the root SCN

WOX5 was shown to be necessary for CSC maintenance, as loss of WOX5 causes their differentiation, while inducible overexpression of WOX5 leads to enhanced proliferation (Sarkar *et al*, 2007; Pi *et al*, 2015; Berckmans *et al*, 2020; Savina *et al*, 2020). In agreement with this, we found that the *wox5* mutants lack a starch‐free cell layer in 78% of analyzed roots, indicating differentiation of the CSCs, compared to 17% in *Col* (Fig 3A, B, F and I, Appendix Table S5). In the *plt2* and *plt3* single mutants, the frequency of roots lacking a CSC layer increases to above 30% (36 and 32%, respectively), and in the *plt2, plt3* double mutant to 41% (see Fig 3C–E and I, Appendix Table S5). After overexpression of PLT3‐mV by estradiol induction in wild‐type *Col‐0* background, we observed the opposite effect, an increase from 29 to 50% of two CSC layers (see Appendix Fig S2A–F). Therefore, we argue, that the observed CSC phenotypes are due to PLT3 function and are not caused by potential early embryonic defects described previously for multiple *plt* mutants (Aida *et al*, 2004).

Interestingly, the *wox5, plt3* double mutant as well as the *wox5, plt2, plt3* triple mutant show a frequency of differentiated CSCs comparable to the *wox5* single mutant (71 and 77%, respectively) (Fig 3G–I, Appendix Table S5). This data suggests that PLTs and WOX5 may act together in the same pathway to maintain CSC homeostasis, as there is no additive effect observable in the multiple mutant roots.

To analyze QC division phenotypes in detail, we quantified the number of EdU‐stained cells in QC position in transversal optical sections. QC cells were identified by their position within the root SCN, as they are located directly distal to the stele initials and surrounded by the CEIs in a circular arrangement (Fig 3A and J). In *Col*, 27% of the analyzed roots show at least one cell division in the QC within the 24 h staining window (Fig 3J, K and R, Appendix Table S5), which is consistent with already published frequencies (Cruz‐Ramírez *et al*, 2013). This frequency almost doubles to 45–50% in the *plt2* and *plt3* single mutants and is even higher in the *plt2, plt3* double mutant (57%) (Fig 3L–N and R, Appendix Table S5). Additionally, the *plt* double mutant roots often show disordered QC regions with a disruption of the circular arrangement of cells surrounding the QC (Fig 3N) which could be a result of uncontrolled divisions. *wox5* mutants show a disordered SCN accompanied by a high overall QC cell division frequency of at least one dividing QC cell in 92% of roots (Fig 3O and R) and on average more dividing QC cells per root (Appendix Table S5). The number of dividing QC cells per root increases further in the *wox5*, *plt3* double mutant and is even higher in the *wox5*, *plt2*, *plt3* triple mutant; here, in one third of the roots all QC cells undergo cell division (Fig 3P–R, Appendix Table S5). Taken together, this data implies an additive effect of PLT2, PLT3, and WOX5 regarding the QC‐division phenotype, suggesting that WOX5 and PLTs act in parallel pathways to maintain the quiescence of the QC.

**Figure EV1:**
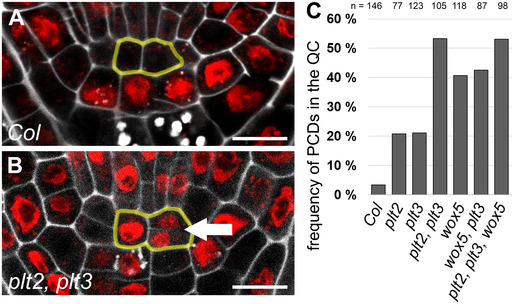
*plt* and *wox5* mutants show more periclinal cell divisions in the QC ARepresentative figure of an *Arabidopsis* wild‐type root SCN staining. QC cells are outlined in yellow. Scale bars represent 10 µm.BRepresentative figure of an *Arabidopsis plt2, plt3* double mutant root SCN staining showing a periclinal cell division (PCD) in the QC (arrow). QC cells are outlined in yellow. Scale bars represent 10 µm.CAnalysis of the PCD phenotype. The frequency of roots (in percent) showing at least one PCD in the QC is plotted as a bar graph. Number of analyzed roots (*n*) (biological replicates) is indicated for each genotype and results from 2 to 5 technical replicates. PCD = periclinal cell division.

Additionally, we quantified roots showing at least one aberrant periclinal cell division in the QC in longitudinal optical sections (Fig EV1). Whereas the occurrence of these aberrant periclinal divisions in *Col* wild‐type roots is very rare (3%) (Fig EV1A), it increases in the *plt*‐single mutants to 21% and in *wox5* and *wox5, plt3* mutants to around 40% (Fig EV1B and C). We found the most severe phenotypes in the *plt2, plt3* double and *wox5, plt2, plt3* triple mutants with an occurrence of periclinal QC‐cell divisions in 53% of the observed roots, indicating a synergistic regulatory role of PLTs in periclinal QC cell divisions (Fig EV1B and C, Appendix Table S6).

### 2D plots of SCN staining facilitate assessment of root phenotypes

**Figure 4:**
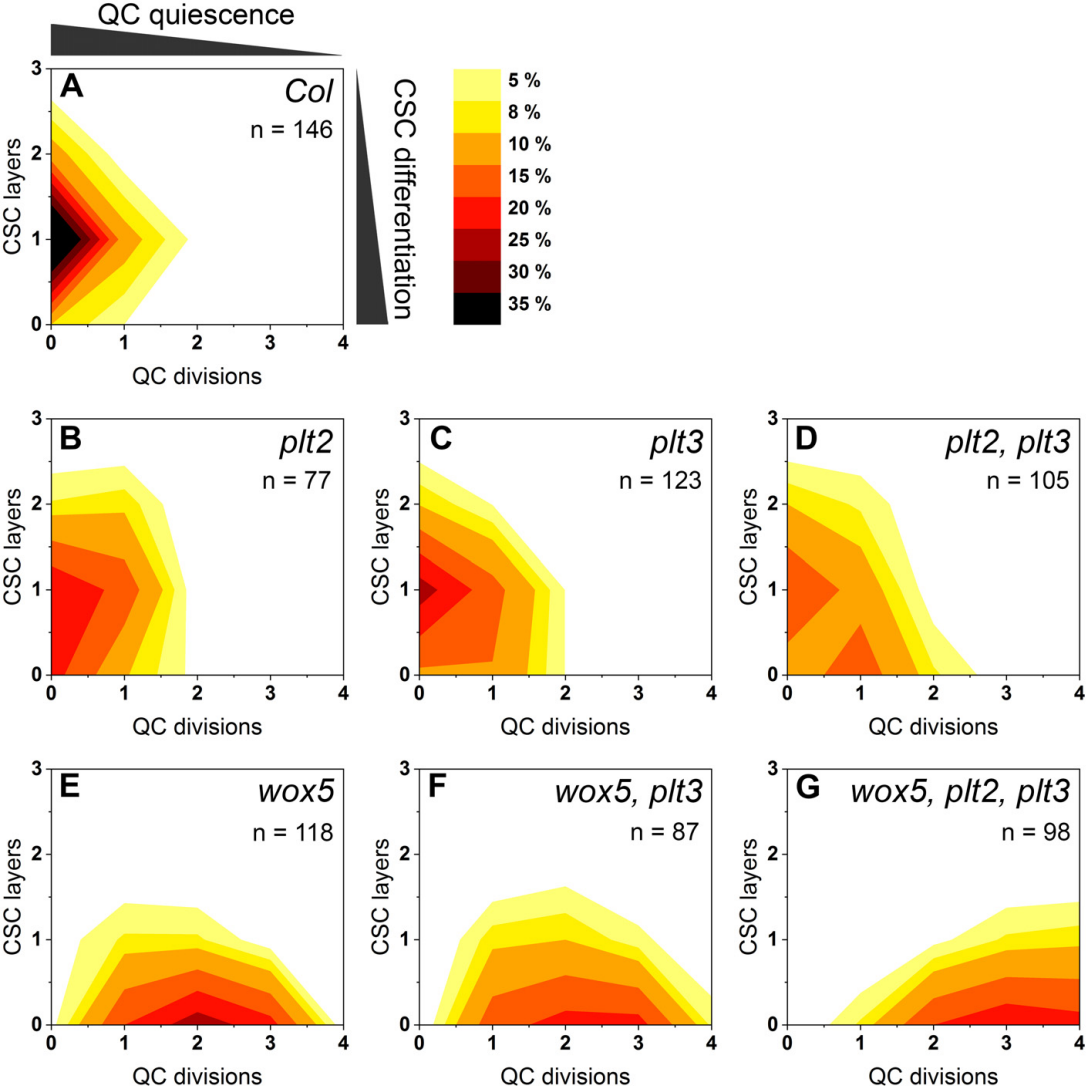
QC divisions correlate negatively with the number of CSC layers A–GThe combined results of the SCN staining in Fig 3 are shown as 2D plots to visualize the correlation of the CSC layer and QC division phenotypes. Number of CSC layers are shown on the y axis and the QC division phenotype is shown on the x‐axis. The darker the color, the more roots show the respective phenotype (see color gradient top right indicating the frequencies in percent). *Col* wild‐type roots show one layer of CSCs and no EdU stained cells (no QC division) after 24 h EdU staining. Number of analyzed roots (*n*) (biological replicates) is indicated for each genotype and results from 2 to 5 technical replicates per genotype. Source data are available online for this figure.

To visualize correlations of QC division and CSC differentiation, we combined the acquired data in 2D‐plots in which the frequencies of the two phenotypes are color‐coded (Fig 4). This visualization reveals a regular pattern for *Col* wild‐type roots, which peaks at one CSC layer and no QC divisions (Fig 4A). The pattern of the *plt* single mutants is more irregular with a shift to less CSC layers (indicating more differentiation) and more EdU‐stained QC cells (indicating more QC divisions) compared to the wild‐type *Col* roots (Fig 4B and C). The *plt2, plt3* double mutants have an additional maximum at a position showing no CSC layer and one divided QC cell, resulting in two phenotypic populations, one at a wild‐type‐like position, the other showing a strong mutant phenotype (Fig 4D). The 2D‐pattern for the *wox5* mutant shifts to less CSC‐layers and more QC‐divisions with a maximum at no CSC‐layers and two QC‐divisions (Fig 4E). The QC phenotype is more severe in the *wox5, plt3* double mutant towards more cell divisions and is even stronger in the *wox5, plt2, plt3* triple mutant which peaks at zero CSC layers and three QC‐divisions (Fig 4F and G). In summary, our data acquired by applying the novel SCN staining demonstrates that higher CSC differentiation correlates with a higher division rate in the QC, possibly to replenish missing stem cells by increased QC divisions.

### PLT3, but not WOX5, localizes to nuclear bodies (NBs)

**Figure EV2:**
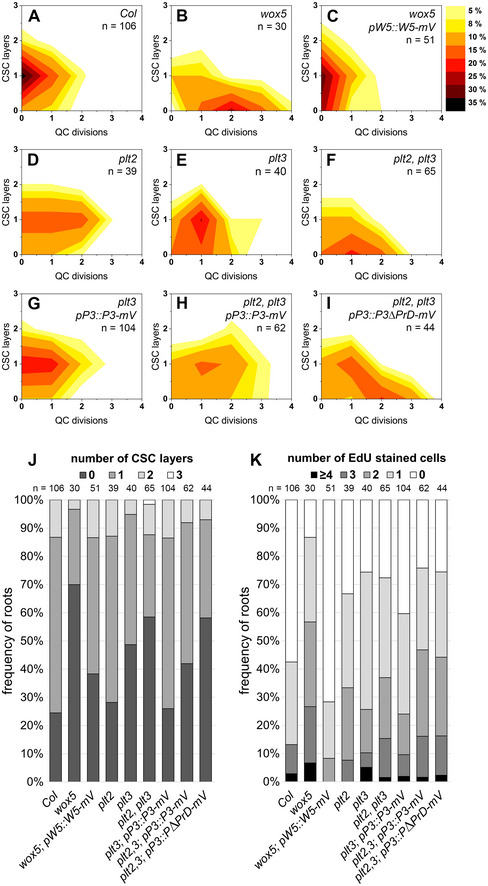
Mutant rescue experiments SCN stainings were performed in *Arabidopsis thaliana* seedlings in the indicated single and double mutant backgrounds expressing either WOX5‐mV, PLT3‐mV or PLT3ΔPrD‐mV driven by their endogenous promoters as well as in *Col* wild type. A–IThe combined results of the SCN staining are shown as 2D plots. Number of CSC layers is shown on the y axis and the QC division phenotype is shown on the x‐axis. The darker the color, the more roots show the respective phenotype (see color gradient on the right indicating the frequencies).J, KAnalyses of the SCN staining for CSC layer (J) or QC division (K) phenotypes. The frequencies of roots showing 0–3 CSC layers, or 0–4 dividing QC cells are plotted as bar graphs. Number of analyzed roots (*n*) (biological replicates) is indicated for each genotype and results from 2 to 4 technical replicates. EdU = 5‐ethynyl‐2′‐deoxyuridine; CSC = columella stem cell; QC = quiescent center; W5 = WOX5, P3 = PLT3.
Source data are available online for this figure.

**Figure 5:**
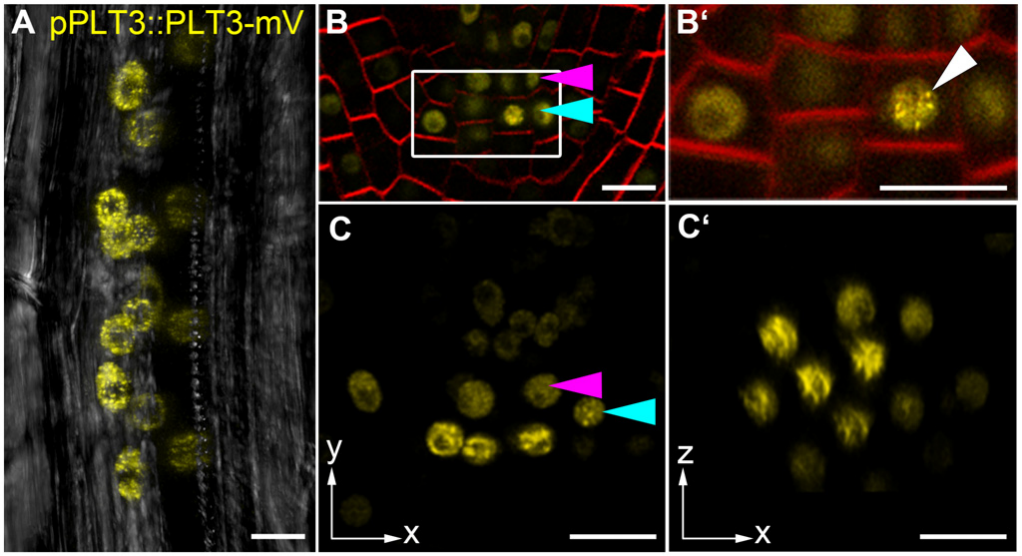
PLT3 localizes to NBs in *Arabidopsis thaliana* LRPs and CSCs A–C′PLT3‐mV expression driven by the PLT3 endogenous promoter in LRP (A) and primary root SCN (B‐C′) in *plt3* mutant *Arabidopsis* roots. (A) Representative image of PLT3‐mV expression (yellow) in an LRP showing the subnuclear localization to NBs. Transmitted light image in grey. (B, B′) SCN of an PLT3‐mV expressing FM4‐64‐stained (red) *Arabidopsis* primary root. The magnification of the CSC layer (B′) shows the subnuclear localization of PLT3 to NBs in a CSC. White arrowhead points at a NB. (C, C’) SCN of an PLT3‐mV expressing *Arabidopsis* primary root. NBs are visible in the CSC layer in (C, also in the transversal view of the CSC layer C′). Arrowheads in B and C point at the QC (magenta) and CSC (cyan) positions. mV = mVenus; LRP = lateral root primordium; SCN = stem cell niche; NBs = nuclear bodies; CSC = columella stem cell. Scale bars represent 10 µm.

**Figure 6:**
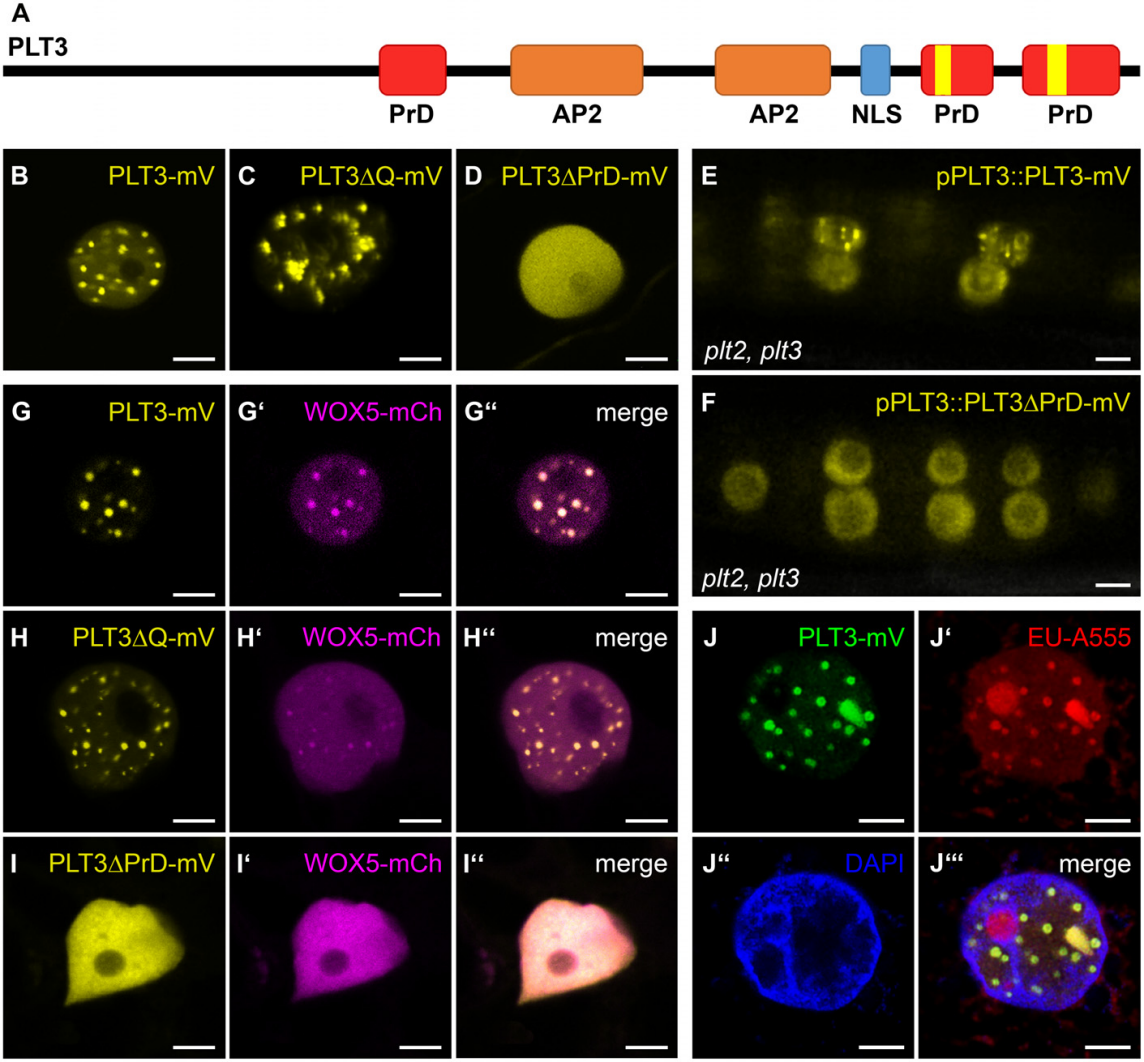
PLT3 PrD domains influence its subnuclear localization ASchematic representation of PLT3 protein domains. The areas in red are predicted prion‐like domains (PrDs) and were deleted in PLT3ΔPrD‐mV. The areas highlighted in yellow contain polyQ‐stretches and were deleted in PLT3ΔQ‐mV.B–DRepresentative images of PLT3‐mV (B), PLT3ΔQ‐mV (C) and PLT3ΔPrD‐mV (D) in transiently expressing *N. benthamiana* leaf epidermal cells.E, FPLT3‐mV (E) and PLT3ΔPrD‐mV (F) expression driven by the PLT3 endogenous promoter in lateral root primordia of *plt2, plt3* double mutant *Arabidopsis* roots.G‐I″Co‐expression of PLT3‐mV (G), PLTΔQ‐mV (H) and PLT3ΔPrD‐mV (I) with WOX5‐mCh (G′, H′, I′) in transiently expressing *N. benthamiana* leaf epidermal cells.J–J‴Expression of PLT3‐mV (J) in transiently expressing *N. benthamiana* leaf epidermal cells in combination with RNA staining with EU (18 h), visualized by click‐reaction with Alexa Fluor^®^ 555 (J′) and a DNA staining with DAPI (J″). Data information: mV = mVenus; PrD = prion‐like domain; AP2 = APETALA2 domain; NLS = nuclear localization signal; EU = 5‐ethynyl‐2′‐uridine. Scale bars in (B–J‴) represent 5 µm.

WOX5 and PLT3 are expressed and localize to overlapping domains in the SCN of the *Arabidopsis* root and based on our results regulate SCN maintenance together. To test for functionality of our generated reporter lines, we used the mVenus (mV) tagged WOX5 and PLT3 versions driven by their own endogenous promoters for rescue experiments in the respective mutant phenotypes in *Arabidopsis*. We observed a rescue of the *wox5* mutant expressing pWOX5::WOX5‐mV (Fig EV2A–C, J and K, Appendix Table S7) and a rescue of the *plt3* mutant expressing pPLT3::PLT3‐mV to almost wild‐type *Col* phenotypes (Fig EV2E, G, J and K, Appendix Table S7), indicating that the labelling with mVenus did not or only very little influence WOX5 or PLT3 functionality. To our surprise, we observed PLT3 localization in bright subnuclear structures, hereafter called NBs, in the PLT3‐mV reporter line. Most frequently, we found PLT3 NBs in young, developing lateral root primordia (LRP) (Fig 5A, Movie EV1) already at stages where PLT1 and PLT2 are not yet expressed (Du & Scheres, 2017). Importantly, we also observed PLT3 NBs in CSCs of some established primary roots, but never in QC cells (Fig 5B and C′). To further examine the PLT3 NBs in a context, where no other PLTs are expressed, we used an estradiol‐inducible system to control expression of FP‐tagged PLT3 and WOX5 transiently in *Nicotiana benthamiana* (*N. benthamiana*) leaf epidermal cells (Stahl *et al*, 2013). In agreement with our observations in *Arabidopsis*, we found that PLT3 mainly localizes to NBs and to a lesser extend to the nucleoplasm (Fig 6B). We quantified NB formation of PLTs in this transient system using estradiol inducible versions of PLT1‐4 tagged with mVenus by acquiring z‐stacks through the expressing nuclei exactly 5 h after induction. Here, we found that PLT3 forms at least three times more NBs compared to PLT1, PLT2 and PLT4 (see Appendix Fig S3A–E, Appendix Table S8).

**Figure EV3:**
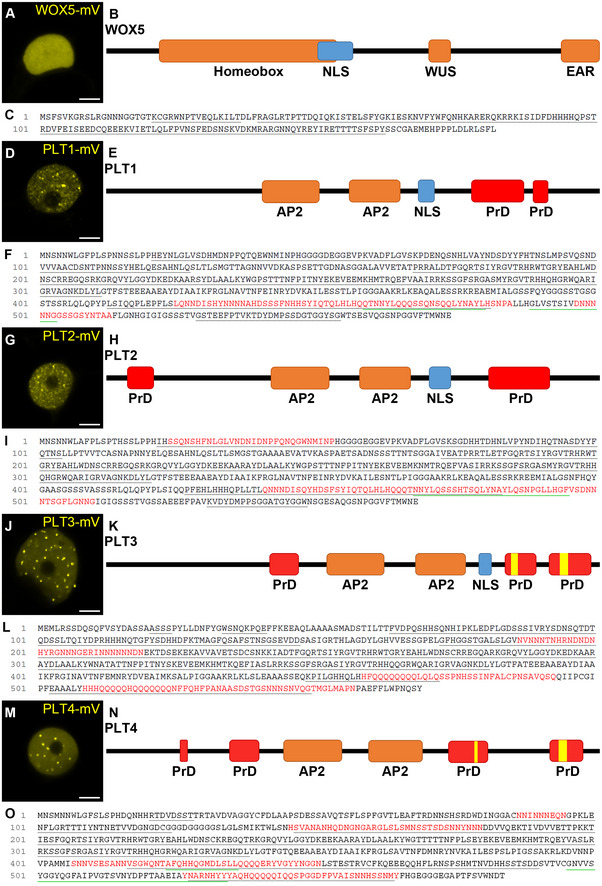
Subnuclear localization and PrD prediction of WOX5, PLT1, PLT2, PLT3, and PLT4 A, D, G, J, MRepresentative images of (sub‐)nuclear localization of WOX5‐mV (A), PLT1‐mV(D), PLT2‐mV (G), PLT3‐mV (J) and PLT4‐mV (m) in transiently expressing *N. benthamina* epidermal cells. Scale bars represent 5 µm.B, E, H, K, NSchematic representation of WOX5 (B), PLT1 (E) PLT2 (H), PLT3 (K), and PLT4 (N) protein domains. The areas in red are predicted prion‐like domains (PrDs), analyzed using the PLAAC prediction tool. Yellow areas are polyQ stretches (defined as more than three Qs in a row) in the PLT3 and PLT4 amino acid sequence.C, F, I, L, OProtein sequences of WOX5 (C) PLT1 (F), PLT2 (I), PLT3 (L), and PLT4 (O). The red highlighted sequences are the predicted prion‐like domains (PrDs). mV = mVenus fluorescent protein; PrD = prion‐like domain; EAR = Ethylene‐responsive binding factor‐associated repression domain; WUS = WUSCHEL box; AP2 = APETALA2 domain; NLS = nuclear localization signal. Source data are available online for this figure.

In co‐expression experiments in *N. benthamiana*, we found that PLT3 recruits WOX5 to the same NBs, whereas on its own WOX5 remains homogenously localized within the nucleoplasm (Fig 6G–G″, Fig EV3A).

### Prion‐like domains (PrDs) are responsible for NB localization of PLT3

Next, we examined the protein domains putatively responsible for the localization of PLT3 to NBs and found that the PLT3 amino acid (aa) sequence contains two glutamine (Q)‐rich regions in the C‐terminal part of the protein (see Figs 6A and EV3K and L). Proteins containing polyQ stretches form aggregates or inclusions, a process often linked to pathological conditions in humans, such as Huntington’s disease (Scarafone *et al*, 2012). However, polyQ proteins can also convey diverse cellular functions like promotion of nuclear assemblies (e.g., the transcription initiation complex), formation of protein‐protein complexes, recruitment of other polyQ‐containing proteins (Mikecz, 2009; Atanesyan *et al*, 2012), and enhancement of the transcriptional activation potential of TFs (Gerber *et al*, 1994; Schwechheimer *et al*, 1998; Atanesyan *et al*, 2012). PolyQ domains were also found to be enriched in plant TFs (Kottenhagen *et al*, 2012).

Next, we tested if the polyQ‐stretches in PLT3 are responsible for the subnuclear localization and the recruitment of WOX5 to NBs. To this end, we deleted the polyQ domains of PLT3 and expressed the resulting PLT3ΔQ fused to mVenus transiently in *N. benthamiana*. We found that the subnuclear localization and the recruitment of WOX5 did not change compared to the full‐length PLT3 (see Fig 6B, C and H–H″). Therefore, we conclude that the polyQ domains in PLT3 are not, or at least not alone, responsible for the subnuclear localization and translocation to NBs.

Apart from proteins with polyQ domains, many proteins that form concentration‐dependent aggregates contain larger, intrinsically disordered regions (IDRs) with a low complexity similar to yeast prions (Cuevas‐Velazquez & Dinneny, 2018). Prion‐like proteins in *Arabidopsis* were first discovered by analyzing protein sequences of 31 different organisms, identifying Q‐ and N‐rich regions in the proteins to be sufficient to cause protein aggregation (Michelitsch & Weissman, 2000). Recently, the existence of more than 500 proteins with prion‐like behavior in *Arabidopsis* was reported (Chakrabortee *et al*, 2016) and the presence of prion‐like domains (PrDs) in protein sequences is predictable with web‐based tools (Lancaster *et al*, 2014). Therefore, we analyzed the PLTs and WOX5 sequences using the PLAAC PrD prediction tool and found that PLT3 has three predicted PrDs in its aa sequence, two of them located at the C terminus, containing the above described two polyQ‐stretches (see Figs 6A and EV3K and L). PLT1, PLT2, and PLT4 also show predicted PrD domains, but PLT1 and PLT2 contain no and PLT4 shorter polyQ stretches within them (Fig EV3D–I and M–O). WOX5 does not show any predicted PrD domains, nor any polyQ stretches (Fig EV3A–C). To test the importance of the PrD domains in PLT3, we replaced the first PrD by a 27 aa linker (AAGAAGGAGGGAAAAAGGAGAAAAAGA) and deleted the C‐terminally located PrDs. The resulting PLT3‐version (PLT3ΔPrD) was fused to the mVenus FP and inducibly expressed in *N. benthamiana* epidermal cells. Here, we did not observe a localization of PLT3ΔPrD‐mVenus to NBs, but in contrast a homogenous distribution within the nucleus (Fig 6D). In addition, upon co‐expression of PLT3ΔPrD‐mVenus with WOX5‐mCherry, we observed that WOX5 was no longer recruited to NBs (Fig 6I–I″). In line with this, we observed PLT3 NBs in developing *Arabidopsis* LRP expressing pPLT3::PLT3‐mVenus, but no more NBs were found in a pPLT3::PLT3ΔPrD‐mVenus expressing line (Fig 6E and F). Based on these observations, we conclude that the PrD domains of PLT3 are responsible for its localization to NBs and the recruitment of WOX5 to NBs. This is further supported by our observation that PLT3, in contrast to PLT1, 2 and 4, is found most frequently in NBs in transiently expressing in *N. benthamiana* correlating with the presence of PrD domains containing long polyQ‐stretches in its aa sequence (Fig EV3D–O, Appendix Fig S3A–E).

### PLT NBs recruit RNA

Proteins containing polyQ‐stretches or PrDs are often involved in RNA binding, RNA processing and/or RNA compartmentalization (Macknight *et al*, 1997; Schomburg *et al*, 2001; Alberti *et al*, 2009; Sonmez *et al*, 2011; Castilla‐Llorente & Ramos, 2014; Fang *et al*, 2019). To test if PLT3 is involved in these processes, we performed an RNA‐staining in *N. benthamiana* epidermal cells transiently expressing PLT3‐mVenus with 5‐ethynyl‐2′‐uridine (EU) (see Fig 6J–J‴). EU is incorporated into RNA during transcription, and we found that most of the stained RNA co‐localizes with the PLT3‐mVenus NBs except for the EU‐stained nucleolus (see Fig 6J–J‴). Based on these observations, we conclude that the PLT NBs act as important sites for the recruitment of RNA and other factors, including WOX5.

### WOX5 and PLT proteins can interact with each other

**Figure 7:**
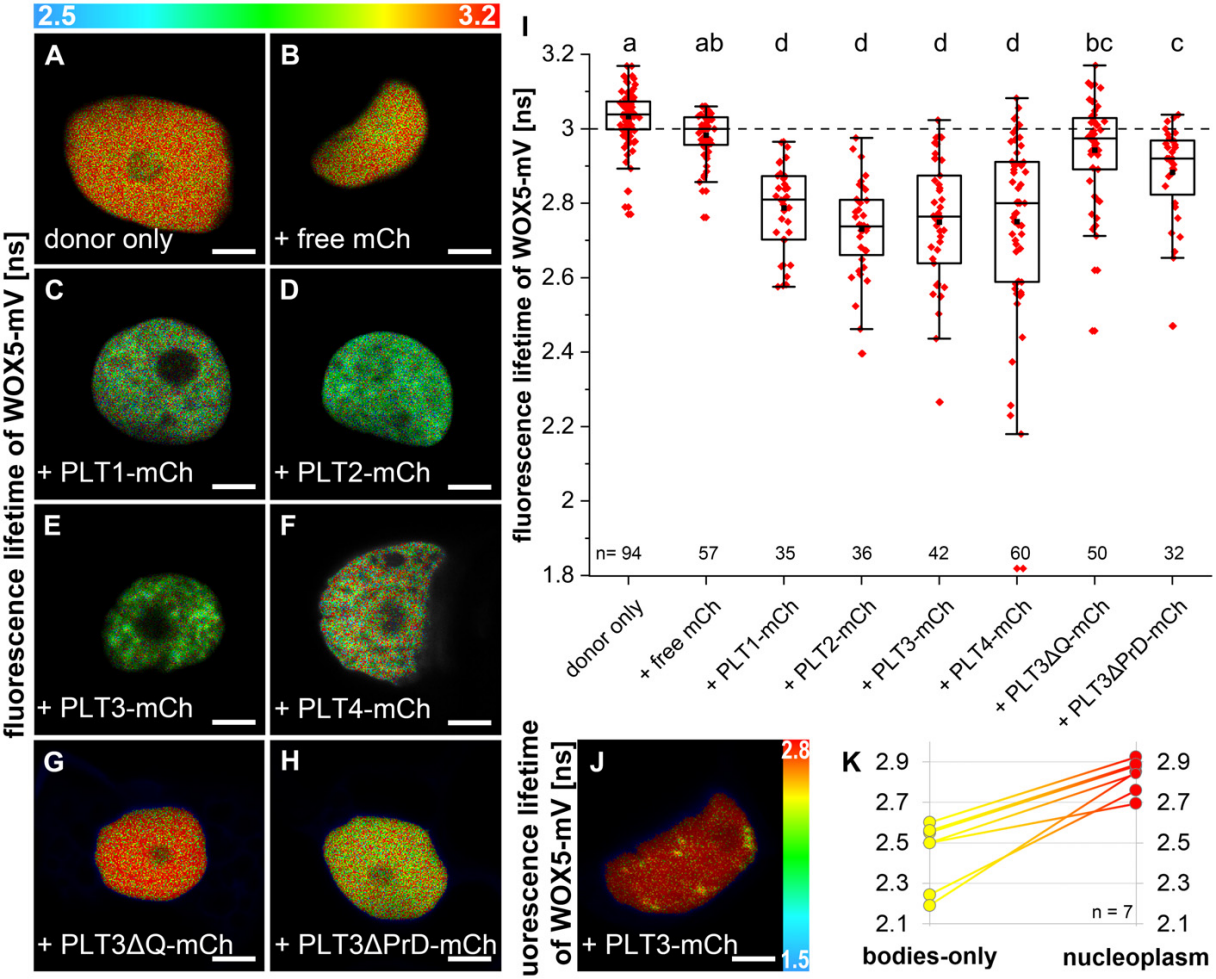
WOX5 can interact with PLTs A–HFluorescence Lifetime Imaging (FLIM) results of transiently expressing *N. benthamiana* leaf epidermal cells. Representative FLIM images of WOX5‐mVenus (donor only) plus the indicated acceptors after a pixel‐wise mono‐exponential fit of the mVenus fluorescence signal. The fluorescence lifetime of WOX5‐mVenus in ns is color‐coded. Low lifetimes (blue) due to FRET indicate strong interaction of the two proteins and high lifetimes (red) indicate weaker or no interaction. Scale bars represent 5 µm.IFluorescence lifetimes in ns are summarized in combined jitter and box plots. The dashed line represents the fluorescence lifetime mean value of the WOX5‐mV co‐expressed with free mCh as negative control. The one‐way ANOVA and Holm–Sidak *post‐hoc* multiple comparisons test was used to test for statistical significance. Samples with identical letters do not show significant differences (α = 0.01). Number of nuclei analyzed (*n*) (biological replicates) is indicated and results from 2 to 9 technical replicates. Box = 25–75% of percentile, whisker = 1.5 interquartile range, − = median, ▪ = mean value.JRepresentative FLIM image of WOX5‐mVenus plus the indicated acceptor after a pixel‐wise mono‐exponential fit of the mVenus fluorescence signal. The fluorescence lifetime of WOX5‐mVenus in ns is color‐coded. Low lifetimes (blue) due to FRET indicate strong interaction of the two proteins and high lifetimes (red) indicate weaker or no interaction. Scale bars represent 5 µm.KIndividual nuclei showing nuclear bodies during co‐expression of WOX5‐mV and PLT3‐mCh were analyzed for WOX5‐mV lifetime in the nuclear bodies or nucleoplasm separately (*n* = 7 (biological replicates), from four technical replicates. mCh = mCherry. mV = mVenus. Source data are available online for this figure.

Because the WOX5 and PLT protein expression domains overlap in the SCN and PLT1, PLT2, PLT3 and PLT4 contain PrD domains, we asked whether PLTs and WOX5 can interact *in vivo*, especially considering the observed recruitment of WOX5 to PLT3 NBs. For this, we used fluorescence lifetime imaging microscopy (FLIM) to measure Förster resonance energy transfer (FRET) to analyze the potential protein‐protein interaction of WOX5 and PLTs *in vivo*. To perform FLIM, we inducibly co‐expressed WOX5‐mVenus as donor together with individual PLTs‐mCherry as acceptors for FRET in *N. benthamiana* leaf epidermal cells. The fluorescence lifetime of the donor fluorophore mVenus fused to WOX5 alone is 3.03 ± 0.07 ns. A reduction of fluorescence lifetime is due to Förster resonance energy transfer (FRET) of the two fluorophores in very close proximity (≤ 10 nm) caused by the direct interaction of the two observed proteins. When free mCherry is co‐expressed as a negative control, the WOX5‐mVenus mean fluorescence lifetime is not significantly decreased (3.00 ± 0.06 ns) (Fig 7A, B and I). When WOX5‐mVenus is co‐expressed with PLT1‐mCherry the fluorescence lifetime significantly decreases to 2.79 ± 0.11 ns, with PLT2‐mCherry to 2.73 ± 0.12 ns, with PLT3‐mCherry to 2.75 ± 0.17 ns and with PLT4‐mCherry to 2.75 ± 0.24 ns, indicating FRET and hence direct protein‐protein interactions (Fig 7C–F and H). The observed interaction of WOX5 with PLT1‐4 lead us to propose that they regulate SCN maintenance by the formation of complexes, either all together or in diverse compositions depending on the cell identity or their function. Interestingly, we observed a stronger lifetime decrease of WOX5‐mVenus in the PLT3 NBs than in the nucleoplasm, indicating that the NBs function as main interaction sites of WOX5 with PLT3 (Fig 7J and K, Appendix Table S9).

**Figure EV4:**
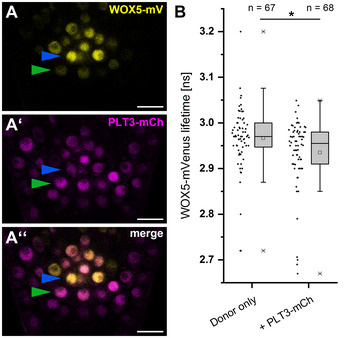
PLT3‐WOX5 interaction in the *Arabidopsis* root A–A′Representative image of the SCN in a lateral root of an *Arabidopsis* reporter line expressing WOX5‐mV (A) and PLT3‐mCh (A′) in *Col‐0* background driven by their respective endogenous promoters. The TFs localize to overlapping domains (A″). Blue arrowheads mark QC cells, green arrowheads mark CSCs. Scalebars represent 10 µm.BFluorescence Lifetime Imaging (FLIM) results of experiments performed in the SCN of lateral *Col‐0* roots expressing either only WOX5‐mV (donor‐only) or both WOX5‐mV and PLT3‐mCh driven by their respective endogenous promoters. Number of analyzed roots (*n*) (biological replicates) is indicated for each genotype and results from 4 technical replicates. Donor fluorescence lifetimes in ns are summarized in combined scatter and box plots. Box = 25–75% of percentile, whisker = 1.5 interquartile range, − = median, □ = mean value, X = minimum/maximum value. The Kruskal–Wallis ANOVA with subsequent Dunn’s test was used to test for statistical significance. Asterisk indicates statistical significance (α = 0.01). mV = mVenus; mCh = mCherry; SCN = stem cell niche.

To address this, we measured the interaction between WOX5 and PLT3 in *Arabidopsis* roots via FLIM experiments in a translational line expressing WOX5‐mVenus and PLT3‐mCherry under control of their respective endogenous promoters. This resulted in the inevitable low protein concentration in comparison to the inducible system used in *N. benthamiana*. Probably due to this, we could not observe NBs in established root meristems of our *Arabidopsis* FLIM line (Fig EV4A–A″) and we could only measure a very small, albeit statistically significant, decrease in fluorescence lifetime from 2.97 ± 0.07 ns in the pWOX5::WOX5‐mVenus (donor only FRET control) expressing roots to 2.94 ± 0.08 ns when pPLT3:PLT3‐mCherry is co‐expressed in the SCN of LRP in *Col‐0* background (Fig EV4B).

### Formation of PLT3 NBs is concentration‐dependent

**Figure EV5:**
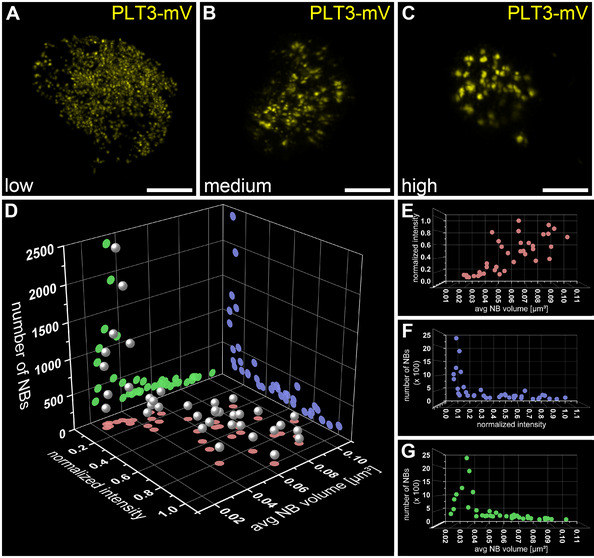
Concentration dependency of PLT3 nuclear body formation A–CRepresentative images of low (A), medium (B), and high (C) PLT3‐mVenus expressing nuclei in transiently expressing *N. benthamiana* leaf epidermal cells are shown. Scale bars represent 5 µm.D–GAnalyses of intensities, numbers, and average volume of PLT3 NBs in individual nuclei, *n* = 37 (biological replicates).

In *Arabidopsis* seedlings, we only sometimes observed PLT3 NBs in the CSC layer of the root tip, but more frequently in young, developing LRP (Fig 5A), whereas in *N. benthamiana* we observed NBs in almost all cells. Therefore, we argue that the formation of the NBs is flexible because it is concentration‐dependent. In a transient *N. benthamiana* experiment, we could observe a correlation between the fluorescence intensity of nuclear PLT3‐mVenus and the size and number of the NBs (Fig EV5). NBs start to form after a certain intensity‐threshold (Fig EV5A–C and F), and their number decreases while their volume increases with overall rising fluorescence intensity (Fig EV5D, E and G). A similar concentration‐dependency has been previously described for proteins that undergo phase separation, in particular for liquid‐liquid phase separation (LLPS) (McSwiggen *et al*, 2019). This mechanism separates membrane‐free microdomains from the surrounding liquid and could potentially represent the underlying NB‐forming process of PLTs. This process is possibly PrD‐dependent as we observed less NB formation in the PrD‐deletion variant of PLT3 (Fig 6D and F).

### PrD‐domains in PLT3 are required for interaction with WOX5 and are necessary for root SCN maintenance

Moreover, we asked if the PrD and polyQ domains in PLT3 are required for protein‐protein interaction with WOX5. To test this, we performed FLIM experiments with mCherry‐tagged full‐length PLT3, PLT3ΔQ, and PLT3ΔPrD as acceptors and WOX5‐mVenus as donor in *N. benthamiana*. Here, we observed that co‐expression of the PLT3ΔQ and PLT3ΔPrD deletion variants did not lead to a significantly reduced fluorescence lifetime, and therefore, no protein‐protein interaction takes place in comparison to the full‐length version (see Fig 7E and G–I). This implies that PrD domains, containing the polyQ domains in PLT3, are necessary for the NB localization, but also, notably, for protein complex‐formation with WOX5. Still, the exact effect of the polyQ domains on PLT‐WOX5 interaction remains to be determined as PLT1, PLT2 and PLT4 still show protein‐protein interaction with WOX5, even though they do not contain extended polyQ domains like in PLT3. We therefore cannot rule out that the deletion of the polyQ domain in PLT3 (PLT3ΔQ) could lead to a disturbed PrD domain resulting in a loss of interaction with WOX5.

Next, we asked if the PrD domains of PLT3 are also required for root SCN homeostasis. Therefore, we tested if the *plt2, plt3* double mutant phenotypes can be rescued by expressing full‐length PLT3 or PLT3ΔPrD under control of the endogenous PLT3 promoter. We observed that full‐length PLT3 expression can rescue the *plt2, plt3* double mutant phenotype of more QC divisions and less CSC layers to the expected levels of *plt2* or *plt3* single mutant phenotypes (Fig EV2D–F, H, J and K, Appendix Table S7). Importantly, PLT3ΔPrD could not rescue the *plt2, plt3* double mutant phenotype, suggesting that the PrD domains in PLT3 are indeed functionally relevant for SCN regulation and maintenance (Fig EV2I–K, Appendix Table S7).

In summary, our findings show that QC quiescence and CSC maintenance are interdependently regulated by WOX5 and PLTs. We could show that mutual transcriptional regulation of PLTs and WOX5, but also their direct protein‐protein interaction is required for QC division and CSC fate regulation. Here, especially the observed subnuclear partitioning of PLT3 to NBs in the CSCs of mature RAMs which is dependent on the presence of PrDs is important as it could provide another layer of regulation to the complex and intertwined SCN maintenance of the *Arabidopsis* root.

## Discussion

In summary, we found that in agreement with a previous report (Sarkar *et al*, 2007), high *PLT* expression in the QC‐region is promoted by WOX5, albeit in an indirect manner, possibly by other factors, such as the described WOX5‐induced TAA1‐mediated auxin biosynthesis (Savina *et al*, 2020; Pardal & Heidstra, 2021). This *PLT* expression confines WOX5 to a defined and restricted number of cells within the QC region. In line with this, loss of PLTs leads to an expanded expression domain of *WOX5* and a reduced QC quiescence reflected in more QC divisions. Therefore, we could confirm that the control of QC quiescence and CSC maintenance is mediated by mutual transcriptional regulation of PLTs and WOX5 possibly by negative feedback loop regulation. As *WOX5* expression is normally limited to the QC, the question arises if, in absence of PLTs, either the *WOX5* expression domain expands to regions surrounding the QC or the QC region itself expands and therefore also the expression domain of *WOX5*. Interestingly, a previous report demonstrated that the expression of several QC markers is missing or highly reduced in *plt* mutants, suggesting that they fail to maintain an intact QC (Aida *et al*, 2004). Therefore, the expansion of the *WOX5* expression domain in the *plt* mutants is likely uncorrelated to the QC identity of these cells.

The observed higher frequency of cell divisions in the QC region of *wox5* mutants could be explained by the reduced expression of *PLTs,* which consequently negatively impacts QC quiescence but also by a PLT‐independent pathway where WOX5 itself may have a positive effect on QC quiescence. Previous findings also suggest that WOX5 maintains QC quiescence through the repression of CYCD activity (Forzani *et al*, 2014). Considering our observation that PLT2, PLT3, and WOX5 show additive effects regarding the QC division phenotype, we propose a model in which WOX5 and PLTs could act in parallel pathways to maintain QC quiescence. The noted correlation of reduced QC quiescence and higher CSC differentiation that we could now show for individual roots by our newly introduced SCN staining could be necessary to replenish missing stem cells by QC divisions. This possible explanation is in agreement with the proposed function of the QC as a long‐term stem cell reservoir, especially in case of stress or damage (Vilarrasa‐Blasi *et al*, 2014).

For CSC homeostasis, PLTs and WOX5 may act together in the same pathway, possibly by complex formation, as there is no observable additive effect in the multiple mutant roots which is in agreement with previous findings (Ding & Friml, 2010). We show that WOX5 can directly interact with PLT1, PLT2, PLT3 and PLT4, which indicates that they regulate CSC maintenance by the formation of complexes, either all together or in diverse homo‐ or heteromeric compositions depending on cell identity or function.

In transient *N. benthamiana* experiments, PLT3 forms NBs and recruits WOX5 into them. The stronger decrease of fluorescence lifetime in NBs compared to the nucleoplasm measured by FLIM implies that the PLT3 NBs function as major sites of protein‐protein interaction with WOX5, which could be due to a favored complex‐formation within the NBs or due to transport of preformed WOX5/PLT3 complexes from the cytosol to NBs. We could observe PLT3 NBs in cells of the CSC layer of some *Arabidopsis* primary root tips, but never in the QC region. Nevertheless, PLT3 NBs were found more frequently in several cells of developing LRPs. LRPs are in a younger and less‐determined stage than the primary root and the observed subnuclear localization to NBs could represent a marker for the occurring determination and future cell differentiation. This agrees with the observed localization of PLT3 to NBs in the CSCs in some of the primary roots. Here, PLT3 NBs could represent compartments for the recruitment of and interaction with WOX5 and possibly other factors involved in CSC fate determination and maintenance.

Furthermore, we found that the aa sequence of PLT3 comprises PrDs (including polyQs) that are necessary for the localization to NBs, complex formation with WOX5, and for SCN maintenance in the primary root meristem. Proteins containing polyQ‐stretches or PrDs are often involved in RNA binding, RNA processing, and/or RNA compartmentalization (Macknight *et al*, 1997; Schomburg *et al*, 2001; Alberti *et al*, 2009; Sonmez *et al*, 2011; Castilla‐Llorente & Ramos, 2014; Fang *et al*, 2019) and we could show that the PLT3 NBs indeed co‐localize with RNA. Just as PLT3, FLOWERING CONTROL LOCUS A (FCA) is a PrD‐containing protein (Chakrabortee *et al*, 2016) that localizes to subnuclear structures (Fang *et al*, 2019). The FCA bodies separate from the cytosol by LLPS to provide compartments for RNA 3′‐end processing factors (Fang *et al*, 2019). Similarly, PLT3 NBs could represent compartments for the recruitment of interacting factors and RNA for further processing, sequestration, or transportation. As PLT3 is a TF, the co‐localizing RNA could also represent newly transcribed RNA at the transcription sites where PLT3 binds to DNA, for example, to the WOX5 promoter region (Shimotohno *et al*, 2018). Another recently described PrD‐ and polyQ‐containing protein, EARLY FLOWERING3 (ELF3), forms NBs by LLPS in response to temperature thereby regulating flowering time in *Arabidopsis* (Jung *et al*, 2020).

The possible liquid‐like nature of the PLT3 NBs will be an interesting subject for further studies investigating its putative phase separation properties in response to external cues. In this study, we showed that the PLT3 NB formation is concentration‐dependent, which is indicative for LLPS. This concentration‐dependency of PLT3 NB formation could also explain the rare occurrence of PLT3 NBs at endogenous protein levels in the CSCs of the *Arabidopsis* RAM. Here, NB formation is possibly triggered only above a certain protein concentration threshold serving as a read‐out for future cell fate. In the established distal root meristem, this is not continuously needed and therefore the protein concentration is mainly maintained below this threshold, so that often no NBs are formed. Consequently, the observed PLT3‐FP fluorescence intensity in the CSCs can vary and is lower or higher at a given time (Figs 1E and 5B and C).

**Figure 8:**
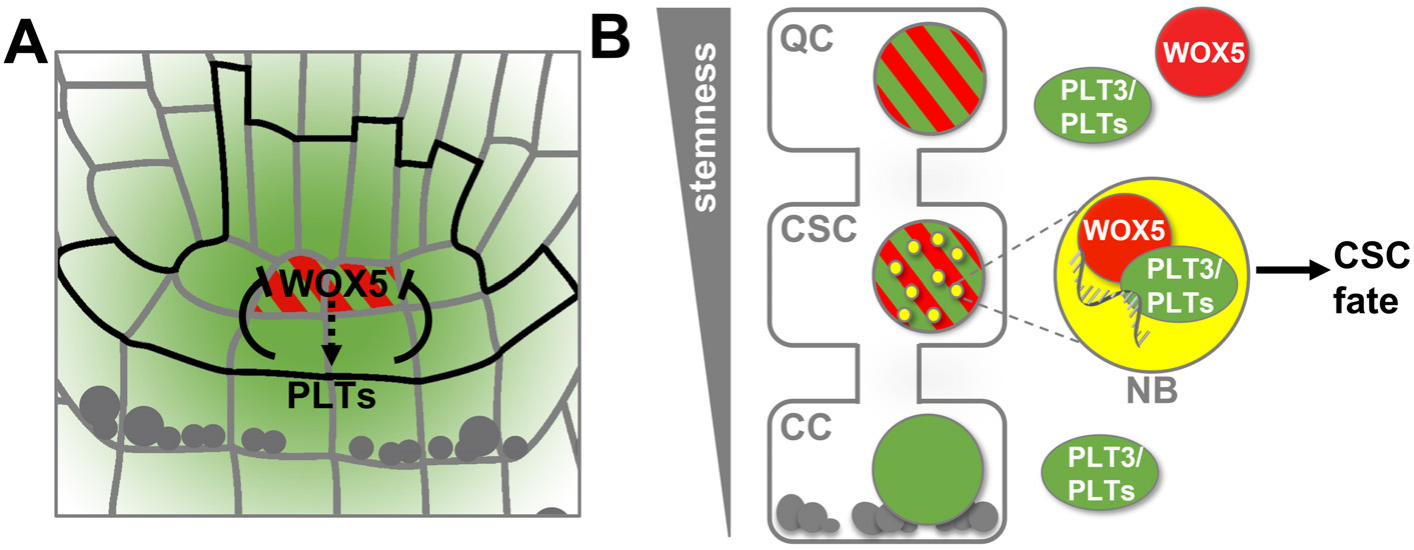
Model of PLT and WOX5 transcriptional regulation, interaction and subnuclear localization during distal root stem cell maintenance Transcriptional regulation of *WOX5* (red) and *PLT* (green) expression by negative feedback regulation in the *Arabidopsis* RAM. *WOX5* is expressed in the QC and indirectly promotes *PLT* expression (dashed arrow), whereas *PLT* expression is directly regulating *WOX5* expression confining it to the QC position (solid barred lines).Both WOX5 (red) and PLT3 (green) are present homogenously within the nuclei of the QC cells. WOX5 can move to the CSCs and is recruited there by PLT3 to NBs (yellow), where interaction takes place and RNA is present (grey in magnification). This maintains the stem cell character of the CSCs (arrow) but already leads to a determination to subsequent CC fate. Gray dots represent starch granules.

Therefore, we propose that the regulation of QC quiescence and CSC maintenance are not only mediated by the mutual transcriptional regulation of PLT and WOX5 (see Fig 8A), but also, importantly, by building protein complexes that are differentially localized to distinct nuclei within the SCN (see Fig 8B). The observed subnuclear localization of PLT3 to NBs could represent a marker for the determination to future cell differentiation in the CSC layer. Furthermore, the PrD domains in PLT3 may act as an initial starting point to compartmentalize and partition WOX5 that has moved from the QC towards the CSC layer into NBs, possibly by a concentration‐dependent LLPS process. The observed sites could represent transcriptionally active sites for the regulation of target genes involved in CSC fate determination or repress WOX5 expression.

The dynamic compartmentalization to subcellular or subnuclear microdomains of proteins with intrinsically disordered, PrD and/or polyQ domains was shown to have severe effects, for example, in human pathological disorders like Huntington’s disease (Scarafone *et al*, 2012). But also beneficial functions of prions that are responsible for some neurodegenerative diseases in mammals (Aguzzi *et al*, 2013; Kim *et al*, 2013) as a protein‐based memory is highly discussed, as their self‐replicating conformations could act as molecular memories to transmit heritable information (Bailey *et al*, 2004; Shorter & Lindquist, 2005). If this is also the case in plants remains to be determined. In general, the dynamic formation of subcellular structures could be necessary for a changing composition of assemblies in dependence of their functional status (Mikecz, 2009). The transition of these proteins between condensed and soluble forms requires high flexibility in their protein structure, which can be provided by flexible intrinsically disordered domains, such as polyQ‐stretches which are predominantly positioned at the surface of a protein, supporting the idea of their involvement in protein‐protein interactions (Totzeck *et al*, 2017).

In *Arabidopsis*, PrD and polyQ dependent compartmentalization could present a fast and reversible concentration‐dependent regulatory mechanism (Cuevas‐Velazquez & Dinneny, 2018), for example, in case of PLT3 and WOX5 to determine CSC cell fate but probably also in other developmental contexts such as lateral root development. It remains to be determined if LLPS is the underlying mechanism of the observed subnuclear compartmentalization of these key TFs involved in *Arabidopsis* root stem cell homeostasis and if also other processes necessary for determination of cell fates and stemness in *Arabidopsis* are regulated by this or similar dynamic processes.

## Materials and Methods

### Cloning

pWOX5::mVenus‐NLS, pWOX5::WOX5‐mVenus, pPLT3::PLT3‐mVenus, pPLT3::PLT3‐mCherry, pPLT3::PLT3ΔPrD‐mVenus, β‐estradiol inducible PLT3ΔPrD‐mVenus, and β‐estradiol inducible PLT3ΔPrD‐mCherry were created by using the GreenGate cloning method (Lampropoulos *et al*, 2013). The internal *BsaI* restriction sites in the WOX5 promoter and WOX5 CDS were removed by PCR amplification of the sequences upstream and downstream of the *BsaI* sites with primer pairs whereof one primer has an altered nucleotide sequence at this site (Appendix Table S10), followed by an overlap extension PCR to reconnect the gene fragments. The sequences upstream of the ATG start codon of WOX5 (4,654 bp) and PLT3 (4,494 bp) were used as promoter regions and were amplified by PCR and primers to add flanking *BsaI* restriction sites and matching overlaps for the GreenGate cloning system. Afterward, they were cloned into the GreenGate entry vector pGGA000 via *BsaI* restriction and ligation. The GreenGate promoter module carrying the β‐estradiol inducible cassette was provided by (Denninger *et al*, 2019). The CDS of WOX5, PLT2, PLT3, PLT3ΔPrD and PLT4 as well as the FPs mVenus and mCherry were amplified by PCR using adequate primer pairs to add flanking *BsaI* restriction sites and matching overlaps for cloning into the GreenGate entry vectors pGGC000 (for CDS) and pGGD000 (for FPs) via *BsaI* restriction and ligation. All created entry vectors were confirmed by sequencing. The expression cassettes were created with a GreenGate reaction using pGGZ001 as destination vector. The correct assembly of the modules was controlled by sequencing. All module combinations used to construct the expression vectors can be found in Appendix Table S11.

All other β‐estradiol inducible constructs for *N. benthamiana* expression (free mCherry, WOX5‐mVenus, WOX5‐mCherry, PLT1‐mVenus, PLT1‐mCherry, PLT2‐mCherry, PLT3‐mVenus, PLT3‐mCherry, PLT3ΔQ‐mVenus, PLT3ΔQ‐mCherry were created by Gateway cloning (Invitrogen™, Thermo Fisher Scientific Inc.). The CDS of WOX5, PLT1, PLT2, PLT3, PLT3ΔQ were amplified and cloned into pENTR/D‐TOPO^®^. The Entry‐vectors were confirmed by sequencing. The destination vector carrying the mVenus (pRD04) is based on pMDC7 (Curtis & Grossniklaus, 2003) which contains a β‐estradiol inducible system for expression *in planta*. The mVenus was introduced via restriction/ligation C‐terminally to the Gateway cloning site. The destination vector carrying the mCherry (pABindmCherry) was described before (Bleckmann *et al*, 2010). The expression vectors were created by LR‐reaction of destination and entry vectors. Gateway expression vectors were verified by test digestion.

For the creation of the domain‐deletion variants of PLT3 (PLT3ΔQ and PLT3ΔPrD), the CDS parts upstream and downstream of the desired sequence deletions were amplified with PCR and afterward reconnected with overlap‐PCR. The 27 aa linker (AAGAAGGAGGGAAAAAGGAGAAAAAGA) to replace the first PrD in PLT3ΔPrD was also introduced by overlap‐PCR. All primers used for cloning can be found in Appendix Table S10. For a list of the constructs produced in this study see Appendix Table S11.

### Plant work

All *Arabidopsis* lines used in this study were in the Columbia (*Col‐0*) background. The single mutants *wox5‐1* and *plt3‐1* have been described before (Galinha *et al*, 2007) (Appendix Table S4). The *plt2* (SALK_128164) and *wox5‐1* (SALK038262) single mutants were provided by the *Arabidopsis* Biological Resource Center (ABRC, USA). The homozygous double and triple mutants were created by crossings (Appendix Table S12) and homozygous F3 es were confirmed by PCR with appropriate primer pairs (Appendix Table S13). The transgenic lines were created by floral dip as described before (Zhang *et al*, 2006) except for the published, transgenic *Col‐0* lines with pPLT3::erCFP and pPLT3::PLT3‐YFP (Galinha *et al*, 2007) constructs. They were crossed into the *wox5‐1* mutant background. Homozygous lines were confirmed by genotyping and hygromycin selection. All plants for crossing, floral dips, genotyping, and seed amplification were grown on soil in phytochambers under long day (16 h light/ 8 h dark) conditions at 21°C. For microscopy *Arabidopsis* seeds were fume‐sterilized (50 ml 13% sodiumhypochlorite (v/v) + 1 ml hydrochloric acid), imbedded in 0.2% (w/v) agarose, stratified at 4°C for 2 days and plated on GM agar plates (1/2 strength Murashige Skoog medium including Gamborg B5 vitamins, 1.2% (w/v) plant agar, 1% (w/v) sucrose, supplemented with 0.05% (w/v) MES hydrate). *Arabidopsis* seedlings were grown for 5 days under continuous light at 21°C and directly imaged afterward.

### Cell wall and plasma membrane staining

For root imaging, the cell walls in *Arabidopsis* seedlings were stained by incubation in aqueous solutions of either 10 µM propidium iodide (PI) or 2.5 µM FM4‐64 dye (Invitrogen™, Thermo Fisher Scientific Inc.). The staining solution was used as mounting medium for microscopy.

### *Nicotiana benthamiana* infiltration

For transient gene expression in *N. benthamiana*, the *Agrobacterium* strain GV3101::pMP50 was used as vector, carrying plasmids with the desired constructs and additionally either the helper plasmid p19 as silencing suppressor or the helper plasmid pSOUP that harbours a replicase needed for GreenGate vectors. Cultures were grown over night in 5 ml dYT‐medium at 28 C on a shaker. The cultures were centrifuged for 10 min at 3,345 *g*, the pellet was resuspended in infiltration medium (5% (w/v) sucrose, 0.01% (v/v) Silwet, 0.01% (w/v) MgSO4, 0.01% (w/v) glucose, 450 µM acetosyringone) to an optical density OD_600_ of 0.4 and cultures were incubated for 1 h at 4°C. The infiltration was done either with one single or with a combination of two different *Agrobacteria* cultures for co‐expression of two constructs. A syringe without needle was used for the infiltration on the adaxial side of the leaves of well‐watered *N. benthamiana* plants. For the expression of GreenGate constructs, an *Agrobacterium* strain carrying the p19 plasmid was co‐infiltrated. The expression was induced 2–5 days after infiltration by applying an aqueous β‐estradiol solution (20 µM β‐estradiol, 0.1% (v/v) Tween^®^‐20) to the adaxial leaf surface. Imaging or FLIM experiments were done 3–16 h after induction, depending on the expression level.

### Quantification of nuclear bodies

NBs were quantified by expression of translational fusions of PLTs to mVenus in *N. benthamiana* after induction with β‐estradiol for exactly 5 h. All image acquisition settings (e.g., laser power, gain, filter, pixel size, averaging, scan speed) were kept the same for the analyses of the different PLTs for comparability. Optical z‐stacks containing the whole nuclei were acquired and the number of NBs per nucleus and the nuclear volumes quantified using Imaris (version 9.1.2, Bitplane, Oxford Instruments plc).

### SCN staining

*Arabidopsis* seedlings were grown under continuous light for 5 days on GM agar plates without sucrose and then transferred on fresh plates containing additionally 7 µg/ml EdU to continue growing for 24 h. Afterward, we performed an mPS‐PI staining like described before (Truernit *et al*, 2008). Preliminary to the clearing step, the EdU‐staining was performed. The permeabilization of the cells and the subsequent staining of EdU‐containing DNA with Alexa Fluor^®^ 488 was done as described in the Click‐it^®^ EdU Imaging Kits from Invitrogen^TM^ (Thermo Fisher Scientific Inc.) with adapted incubation times for *Arabidopsis* seedlings (permeabilization for 1–2 h and click‐reaction for 1 h). The click‐reaction cocktail was prepared freshly with self‐made solutions (Tris buffer with 50 mM Tris and 150 mM NaCl at pH 7.2–7.5; 4 mM CuSO4; 1.5 µM Alexa Fluor^®^ 488 picolyl azide; 50 mM ascorbic acid). The Alexa Fluor^®^ 488 picolyl azide (Thermo Fisher Scientific Inc.) was added from a 500 µM stock in DMSO. The ascorbic acid was added last from a freshly prepared aqueous 500 mM stock solution. After staining was done, the clearing step with chloralhydrate was performed like described before (Truernit *et al*, 2008). Images were acquired with a ZEISS LSM880 confocal microscope with imaging acquisition settings as stated below. z‐stacks through the QC‐region were recorded to obtain transversal views. To calculate the CSC phenotype, the number of CSC layers was counted in xy‐images of each root. For this, we defined an intact CSC layer as a stem‐cell layer below QC position without any differentiated cells (visible by no starch‐accumulation). Cells in the layer below QC position containing starch granules were scored as differentiated, and we therefore score the whole layer as differentiated, even if only some of these cells contain starch.

The QC‐division phenotype is the number of EdU‐Alexa Fluor^®^ 488‐stained cells in the QC, which was counted in the cross‐sectional images up to a maximal number of 4 stained QC cells. In case of duplicated QC cells as in *plt2, plt3* double mutants only one layer of QC cells, the one with the most QC divisions, was counted (usually the bottom layer).

The phenotype frequencies of CSC differentiation and QC divisions (Fig 3) where visualized in bar graphs with Excel (Microsoft Office 365 ProPlus, Microsoft Corporation). In order to correlate the two investigated phenotypes, we combined the CSC data and the QC‐division data in 2D‐plots. The combined QC/CSC‐phenotype of every root was entered in a matrix with QC‐divisions on the x‐ and CSC layers on the y‐axis. 2D plots were created with Origin 2018b and 2020b (OriginLab Corporation).

### RNA staining

RNA‐staining in *N. benthamiana* epidermal cells was done on *N. benthamiana* leaves harboring a construct for a β‐Estradiol inducible *PLT3‐mVenus* expression. 5‐ethynyl‐2′‐uridine (EU) was infiltrated in *N. benthamiana* leaves the day before staining. The expression of PLT3‐mVenus was induced the next morning, 3 h before fixation of the plant tissue. For fixation and permeabilization of cells, pieces of the leaves were cut and treated with 4% (w/v) paraformaldehyde and 0.5% (v/v) TritonX‐100 in PBS under vacuum for 1 h. The click‐reaction of EU with Alexa Fluor^®^ 555 picolyl azide was performed similarly to the EdU‐Alexa Fluor^®^ 488‐staining described for the SCN staining in this article. A DAPI‐ counterstaining was carried out afterward by incubating the leaf pieces in 0.1 µg/ml DAPI for 30 min. PBS was used as mounting medium for imaging.

### Microscopy

Imaging of *Arabidopsis thaliana* roots and *Nicotiana benthamiana* leaves was carried out with a ZEISS LSM780 or LSM880. Excitation and detection of fluorescent emission of fluorescent dyes was done as follows: DAPI was exited at 405 nm and emission was detected at 408–486 nm, Cerulean was excited at 458 nm and emission was detected at 460–510 nm; CFP was excited at 458 nm and emission was detected at 463–547 nm. mVenus was excited at 514 nm and emission was detected at 517–560 nm, or for co‐expression with red dyes excited at 488 nm and detected at 500–560 nm. YFP was excited at 514 nm and emission was detected at 518–548 nm. Alexa Fluor^®^ 488 was excited at 488 nm and emission was detected at 490–560 nm. Alexa Fluor^®^ 555 was excited at 561 nm and emission was detected at 565–640 nm. PI was excited at 561 nm and emission was detected at 590–710 nm. FM4‐64 was excited at 514 or 561 nm and emission was detected at 670‐760 nm. mCherry was excited at 561 nm and emission was detected at 590–640 nm. Imaging of more than one fluorophore was done in sequential mode to avoid cross talk. The movie of pPLT3::PLT3‐mVenus in a lateral root primordium was acquired with a MuViSPIM (Luxendo, Bruker) light sheet microscope equipped with a 40×/0.8 Nikon objective with a 1.5× tube lens on the detection axis to provide a 60× magnification.

### Image deconvolution

The microscope images in Fig 5A and C–C′ were deconvolved with Huygens 16.10.0p3 64b (Scientific Volume Imaging B.V.).

### Analyses of expression patterns and levels in *Arabidopsis*

For the comparison of relative fluorescence levels in the SCN of 5‐day‐old *Arabidopsis* seedlings expressing either transcriptionally FP tagged PLT3 (pPLT3::erCFP) or translationally FP tagged *PLT3* (pPLT3::PLT3‐YFP) driven by the endogenous PLT3 promoter in either the *Col* wild type or the *wox5‐1* mutant, images were acquired with constant settings per FP. A ZEISS LSM880 confocal microscope was used. The mean fluorescence levels were measured with Fiji (Schindelin *et al*, 2012) in equally sized rectangular ROIs including the QC and CSC positions in the SCN. The thereby generated values were normalized to the *Col* mean fluorescence intensity and visualized in box and scatter plots created with Origin 2018b (OriginLab Corporation).

Images of the root tips of 5 day old *Arabidopsis* seedlings expressing *mVenus‐NLS* driven by the endogenous WOX5 promoter in *Col* and *plt2* or *plt3‐1* single mutants and the *plt2,plt3* double mutant were acquired. Additionally, z‐stacks through the QC region of the roots were recorded to get a transversal view of the QC. The visualization and counting of nuclei with *WOX5* expression (Fig 2) was done with Imaris (version 9.1.2, Bitplane, Oxford Instruments plc). Box and scatter plots showing the number of expressing nuclei were created with Origin 2018b and 2020b (OriginLab Corporation). For the heat‐map images, 10 acquired images were overlaid with Fiji (Schindelin *et al*, 2012) and the resulting fluorescence distribution was displayed with a 16‐colors lookup table. To calculate the area of lateral *WOX5* expression in the QC region, a freehand‐ROI surrounding the expressing cells was created in every image with Fiji (Schindelin *et al*, 2012). The ROI‐areas were visualized in box and scatter plots, and all statistical analyses were carried by using Origin 2018b and 2020b (OriginLab Corporation).

### Measurement of *PLT3* expression upon WOX5 induction

For qPCR analyses of *PLT3* expression after WOX5 induction, *Arabidopsis thaliana* p35S:WOX5‐GR seeds were sterilized, stratified, and sown on GM plates without sucrose as described above onto a 20 µm nylon mesh for easy and fast transfer. The seedlings were grown under continuous light conditions at 21°C and 60% humidity for 5 days. For cycloheximide (CHX) and CHX + dexamethasone (DEX) treatments, the seedlings were transferred to GM plates without sucrose containing 10 µM CHX (Sigma) for 15 min and then transferred to GM plates without sucrose containing 10 µM CHX or 10 µM CHX and 20 µM DEX (Sigma), respectively. For DEX induction tests, seedlings were transferred to GM plates without sucrose containing 20 µM DEX. As a control, seedlings were transferred to GM plates without sucrose containing 10 µM DMSO. To ensure uptake of CHX and/or DEX, seedlings were additionally sprayed with a solution containing 0.1% Tween‐20 and 10 µM CHX or 20 µM DEX or both. Isolation of RNA from roots was performed with the “RNeasy Plant Mini Kit” (Qiagen). For cDNA synthesis, 1 µg total root RNA per sample was reverse‐transcribed using SuperScript III first strand synthesis kit (Invitrogen) according to the manufacturer’s protocol. Quantitative real‐time PCR (qPCR) was performed using Luna^®^ Universal qPCR Master Mix (NEB) according to manufacturer’s instructions on a Stratagene Mx3005P (Agilent technologies). Data normalization was performed as described before and *ACTIN* used as reference gene (Livak & Schmittgen, 2001). qPCR primers are listed in Appendix Table S13.

F1 generation seeds of a crossing of *Arabidopsis* lines carrying 35S::WOX5‐GFP‐GR or pPLT3::erCFP in *Col‐0* background were grown as described above at 21°C and continuous light for 5 days. For DEX induction, seedlings were treated with 20 µM DEX. Images were taken 4 or 21 h after treatment, respectively. To visualize the cell walls, seedlings were mounted in 25 µM propidium iodide staining solution prior to imaging. Imaging was carried out with a ZEISS LSM780, with two different tracks: Track 1 was used for simultaneous detection of GFP and PI with a 488/561 nm beam splitter, track 2 was used for CFP detection with a 458 nm beam splitter. GFP was excited at 488 nm and was detected at 500–535 nm and PI was excited at 561 nm and was detected at 597–663 nm. CFP was excited at 458 nm and was detected at 464–509 nm and tracks were acquired framewise to avoid crosstalk. Laser power and detector gain were kept the same during the experiment and the same acquisition settings were used for all images. For quantification of fluorescence intensities, three different ROIs were chosen with a size of 200 × 150 or 70 × 180 pixels, respectively, and were arranged at the same location within the root during the experiment. For each ROI, the ratio of CFP to GFP fluorescence intensity was calculated and normalized to the control group of the same ROI.

### FLIM measurements

FLIM was performed either in *N. benthamiana* leaf epidermal cells expressing the desired gene combinations or in roots of 6‐10 dag old *Col‐0 Arabidopsis* seedlings expressing WOX5‐mVenus and PLT3‐mCherry with their endogenous promoters. The FLIM measurements of the SCNs in *Arabidopsis* were performed in LRPs due to higher fluorescence levels and less movement during measurements compared to the main RAM. mVenus‐tagged proteins were always used as donor and mCherry‐tagged proteins as acceptor for FRET. A ZEISS LSM 780 was used for the experiments equipped with a time correlated single‐photon counting device (Hydra Harp 400, PicoQuant GmbH). The mVenus donor was excited with a linearly polarized diode laser (LDH‐D‐C‐485) at 485 nm and a pulse frequency of 32 MHz. The excitation power was adjusted to 0.1–0.5 µW at the objective (C‐Apochromat 40×/1.2 W Corr M27, ZEISS) for experiments in *N. benthamiana* and 1.5–2 µW for experiments in *Arabidopsis*. The higher laser power in *Arabidopsis* was needed due to lower fluorescence levels. τ‐SPAD single photon counting modules with 2 channel detection units (PicoQuant GmbH) and a bandpass filter (534/30) were used to detect parallel and perpendicular polarized emission of the mVenus fluorescence. Images were acquired with a frame size of 256 × 256 pixel, a pixel dwell time of 12.6 µs and a zoom factor of 8. 40–60 frames were recorded in the *N. benthamiana* experiments, 80 frames in the experiments performed in *Arabidopsis*.

Fluorescent lifetimes were obtained by further analyses of the acquired data with SymPhoTime64 (PicoQuant GmbH). The instrument response function (IRF) of the microscope hardware is needed for fluorescence lifetime calculation to correct the system‐specific internal time lag between laser pulse and data acquisition. The IRF was recorded preliminary to each experiment by time‐correlated single photon counting (TCSPC) of an aqueous solution of erythrosine B in saturated potassium iodide. For data analysis of *N. benthamiana* experiments, an intensity threshold of 100–200 photons per pixel was applied to remove background fluorescence and a monoexponential fit was used. Due to low fluorescence intensities in *Arabidopsis* experiments, no threshold was applied to obtain the maximal possible photon number. In this case, a two‐exponential fit was used to separate the mVenus fluorescence signal from the background fluorescence created by the plant tissue. This results in two lifetimes whereof one matches with the mVenus fluorescence lifetime of about 3 ns and the other representing the very short background lifetime of < 0.4 ns. All data were obtained in at least two independent experiments. For visualization of the lifetimes, box and scatter plots were created with Origin 2020b (OriginLab Corporation). Lifetime images of representative measurements were created with a pixel wise FLIM‐fit in SymPhoTime64 (PicoQuant GmbH). The line graph showing the lifetime difference between the bodies and the nucleoplasm of WOX5‐mVenus co‐expressed with PLT3‐mCherry was created using Excel (Microsoft Office 365 ProPlus, Microsoft Corporation).

### Statistical testing

Data were tested for normal distribution by Kolmogorov‐Smirnov testing. In case of normal distribution below 0.05 niveau, the data were subsequently analyzed by one‐way ANOVA and *post‐hoc* Holm–Sidak multiple comparisons test with a = 0.01 to identify statistically significant differences. In case of non‐normal distribution of the data, the non‐parametric Kruskal–Wallis ANOVA with subsequent Dunn’s test was used to test for statistical significance (a = 0.01). Statistical tests were carried out in Origin 2020b (OriginLab Corporation) or in R.

### Prediction of protein domains

The PrDs in the WOX5, PLT1, PLT2, and PLT3 aa sequences were predicted using the PLAAC application (Lancaster *et al*, 2014). The nuclear localization signals (NLSs) of WOX5 and the studied PLT proteins were predicted using the cNLS Mapper (Kosugi *et al*, 2009) for WOX5 and PLT3 and SeqNLS (Lin & Hu, 2013) for PLT1 and PLT2.

### Figure assembly

All figures in this study were assembled using Adobe Photoshop (Adobe Inc.).

## Author contributions

**Rebecca C Burkart:** Conceptualization; Data curation; Validation; Investigation; Visualization; Methodology; Writing – original draft. **Vivien I Strotmann:** Data curation; Investigation; Writing – review & editing. **Gwendolyn K Kirschner:** Investigation. **Abdullah Akinci:** Investigation. **Laura Czempik:** Investigation. **Anika Dolata:** Investigation; Visualization. **Alexis Maizel:** Investigation; Visualization. **Stefanie Weidtkamp‐Peters:** Validation. **Yvonne Stahl:** Conceptualization; Data curation; Formal analysis; Supervision; Funding acquisition; Validation; Investigation; Methodology; Writing – original draft; Project administration; Writing – review & editing.

In addition to the CRediT author contributions listed above, the contributions in detail are:

YS conceived the project. YS, RCB and VIS designed the experiments, analyzed and interpreted the data. RCB, VIS, AA, AD, LC, and GKK carried out experiments. SW‐P contributed to FLIM data analyses. AM carried out light sheet imaging. YS and RCB wrote the manuscript. All authors commented on the manuscript.

## Disclosure and competing interests statement

The authors declare that they have no conflict of interest.

## Supporting information



Appendix

Expanded View Figures PDF

Movie EV1

Source Data for Expanded View and Appendix

Source Data for Figure 3

Source Data for Figure 4

Source Data for Figure 7

## Data Availability

No primary datasets have been generated and deposited.

## Footnotes

^§^
Correction added on 7 June 2022, after first online publication: The authors became aware of a publication that describes a different combined mPSPI and EdU staining protocol for Arabidopsis floral meristems and would like to acknowledge this work (Schiessl *et al*, 2012).
