# Supplementary material for: PLETHORA‐WOX5 interaction and subnuclear localization control Arabidopsis root stem cell maintenance
Source: EMBO Rep. 2022 Apr 4;23(6):e54105. doi: 10.15252/embr.202154105 (PMC9171415; doi:10.15252/embr.202154105)
Supplement: Supplementary file 1 — Appendix [file EMBR-23-e54105-s005.pdf]

Appendix

Table of content

Appendix Figure S1: Quantification of *PLT3* expression after WOX5 induction 2
Appendix Figure S2: Ectopic expression of PLT3-mV leads to supernumerary CSC layers 3
Appendix Figure S3: Quantification of NBs in PLT1-4.. 4
Appendix Table S1: *PLT3* expression after WOX5 induction related to Appendix Figure S1A............ 5
Appendix Table S2: *PLT3* expression after WOX5 induction related to Appendix Figure S1B-E 6
Appendix Table S3 Intensity values of transcriptional and translational FP-tagged *PLT3* expression experiments in *Col-0* and *wox5* related to Figure 1.. 7
Appendix Table S4: Quantification of transcriptional mVenus-tagged *WOX5* expression in the QC region of *Col* and *plt2, plt3* related to Figure 2 8
Appendix Table S5: Average QC and CSC phenotypes related to Figures 3 and 4................................ 9
Appendix Table S6: Percentage of periclinal cell divisions in the QC shown in Figure EV1 10
Appendix Table S7: Average QC and CSC phenotypes of rescue experiments shown in Figure EV2 11
Appendix Table S8: Quantification of NBs in PLT1-4 related to Appendix Figure S3........................ 12
Appendix Table S9: FLIM results of subnuclear data analysis in WOX5-mV and PLT3-mCh co-expressing *N. benthamiana* epidermal cells related to Figure 7K.. 13
Appendix Table S10: List of cloning primers 14
Appendix Table S11: List of expression vectors created in this study.. 15
Appendix Table S12: *Arabidopsis* mutants and transgenic lines used in this study 16
Appendix Table S13: List of genotyping and qPCR primers... 17
References 18

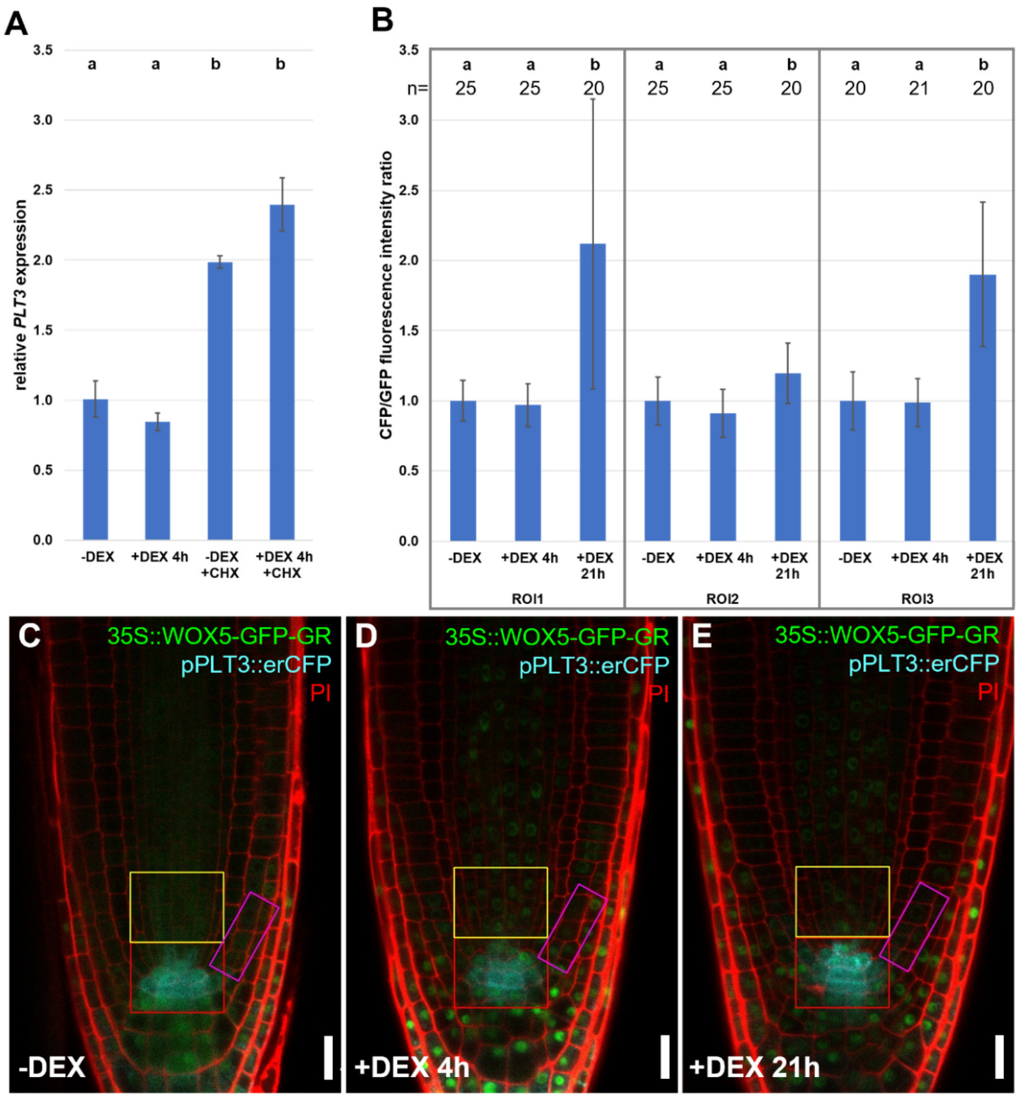

Appendix Figure S1: Quantification of *PLT3* expression after WOX5 induction

A, qPCR analyses on root RNA isolated from 5 days old 35S:WOX5-GR *Arabidopsis* seedlings uninduced or induced for 4 h with 20 µM dexamethasone (DEX) with or without simultaneous incubation with 10 µM cycloheximide (CHX). Three biological replicates and two technical replicates per treatment were carried out. The data was normalized to the -DEX sample. Mean values ± standard deviations are shown. **B**, Quantification of CFP/GFP fluorescence intensity ratios in 5 days old *Arabidopsis* roots of F1 crosses (35S::WOX5-GFP-GR × pPLT3::erCFP) without induction (-DEX), or induction with dexamethasone (+DEX) for 4 or 21h. The data was normalized to the not induced roots (-DEX) per analyzed region of interest (ROI). Mean values ± standard deviations are shown. The data in A and B was statistically analysed by one-way ANOVA and Holm-Sidak post-hoc multiple comparisons test. Samples with identical letters do not show significant differences ($\alpha = 0.01$). The number of roots (n) (biological replicates) is indicated for each sample and results from two independent technical replicates. **C-E**, representative images of the roots analyzed in B. Cell walls are stained by propidium iodide (PI) in red. ROI1 is marked in red, ROI2 is marked in yellow, ROI3 is marked in magenta. Scalebars represent 10 µm.

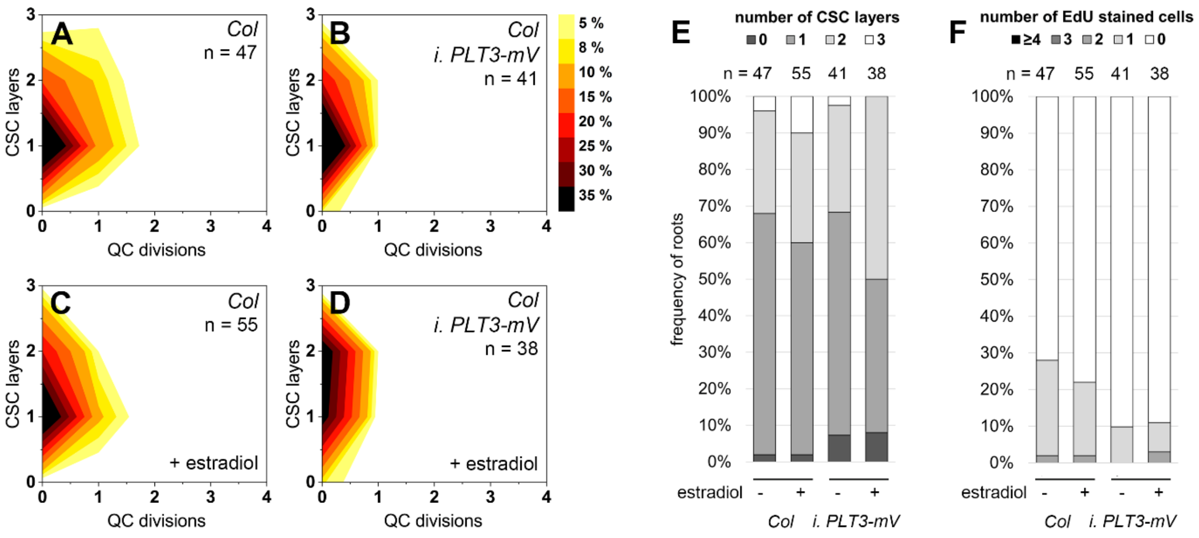

Appendix Figure S2: Ectopic expression of PLT3-mV leads to supernumerary CSC layers

SCN stainings were performed for 24 h on five days old *Arabidopsis thaliana Col-0* seedlings (**A**, **C**) or *Col-0* carrying an estradiol inducible PLT3-mV construct (**B**, **D**). A, B, non-induced seedlings. **C**, **D**, seedlings induced with 25 µM β-estradiol. **A-D**, combined results of the SCN staining are shown as 2D plots. Number of CSC layers are shown on the y axis and the QC division phenotype is shown on the x axis. The darker the colour, the more roots show the respective phenotype (see colour gradient on the right indicating the frequencies). **E**, **F**, Analyses of the SCN staining for CSC layer (**E**) or QC division (**F**) phenotypes. The frequencies of roots showing 0 - 3 CSC layers or 0 - 4 dividing QC cells are plotted as bar graphs. Number of analysed roots (n) (biological replicates) is indicated for each genotype and results from three independent technical replicates. EdU = 5-ethynyl-2′-deoxyuridine; i = inducible; CSC = columella stem cell; QC = quiescent centre.

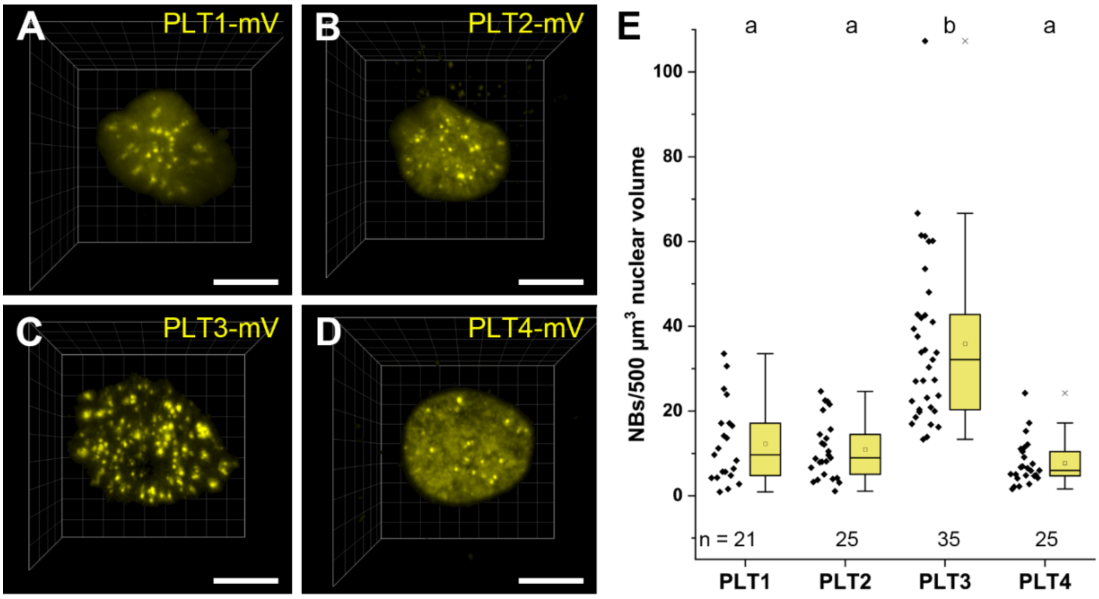

Appendix Figure S3: Quantification of NBs in PLT1-4

A-D, Representative 3D images of nuclei expressing PLT1-mV (**A**), PLT2-mV (**B**), PLT3-mV (**C**), and PLT4-mV (**D**) in *N. benthamiana* leaf epidermis 5 hours after induction with β-estradiol. Scalebars represent 10 µm. **E**, quantification of the number of NBs per 500 μm^3 nuclear volume summarized in box plots. Box = 25 – 75 % of percentile, whisker = 1.5 interquartile range, – = median, □ = mean value, × = outliers. The Kruskal-Wallis ANOVA analysis with subsequent Dunns test was used to test for statistical significance. Samples with identical letters do not show significant differences ($\alpha = 0.01$). The number of nuclei (n) (biological replicates) is indicated for each sample and results from two independent technical replicates. NBs = nuclear bodies; mV = mVenus.

Appendix Table S1: ***PLT3*** **expression after WOX5 induction related to Appendix Figure S1A**

Sample	PLT3 Ct1	PLT3 Ct2	PLT3 Ct MV	ACTIN Ct1	ACTIN Ct2	ACTIN Ct MV	ΔCt	ΔΔCt	2^-(ΔΔCt)
-DEX 1	23.89	23.89	23.89	19.09	19.18	19.14	4.76	0.07	0.95
-DEX 2	24.31	24.28	24.30	19.84	19.88	19.86	4.44	-0.25	1.19
-DEX 3	23.81	23.84	23.83	18.91	19.03	18.97	4.86	0.17	0.89
+DEX 4h 1	23.94	23.85	23.90	18.83	18.82	18.83	5.07	0.39	0.76
+DEX 4h 2	24.74	24.42	24.58	19.65	19.72	19.69	4.90	0.21	0.86
+DEX 4h 3	23.56	23.51	23.54	18.78	18.66	18.72	4.82	0.13	0.91
+CHX 1	21.79	21.88	21.84	18.28	18.03	18.16	3.68	-1.00	2.00
+CHX 2	22.31	22.34	22.33	18.65	18.53	18.59	3.74	-0.95	1.93
+CHX 3	21.98	22.12	22.05	18.24	18.54	18.39	3.66	-1.02	2.03
+DEX 4h +CHX 1	22.66	22.80	22.73	19.16	19.16	19.16	3.57	-1.11	2.16
+DEX 4h +CHX 2	22.88	22.75	22.82	19.39	19.41	19.40	3.42	-1.27	2.41
+DEX 4h +CHX 3	23.11	22.85	22.98	19.71	19.67	19.69	3.29	-1.39	2.62
Calibrator (average ΔCt -DEX)	4.68								

Descriptive statistics:

	MV	SD	SE
-DEX	1.01	0.16	0.09
+DEX 4h	0.85	0.06	0.04
+CHX	1.99	0.05	0.03
+DEX 4h +CHX	2.40	0.19	0.11

qPCR analysis on root RNA isolated from 5 days old 35S:WOX5-GR *Arabidopsis* seedlings uninduced or induced for 4 h with 20 µM dexamethasone (DEX) with or without simultaneous incubation with 10 µM cycloheximide (CHX). Three biological replicates and two technical replicates per sample were carried out. The data was normalized to the -DEX sample using ACTIN as reference gene according to (Livak & Schmittgen, 2001). MV = mean value, SD = standard deviation, SE = standard error.

Appendix Table S2: ***PLT3*** **expression after WOX5 induction related to Appendix Figure S1B-E**

Technical replicate	ROI1			ROI2			ROI3		
	-DEX	+DEX 4h	+DEX 21h	-DEX	+DEX 4h	+DEX 21h	-DEX	+DEX 4h	+DEX 21h
1	0.87	0.82	1.50	0.70	0.61	1.22	1.04	1.03	1.65
	0.60	0.74	1.86	0.75	0.71	1.12	0.89	0.81	1.69
	0.98	1.03	1.83	1.09	0.66	1.22	1.07	0.61	1.75
	1.09	0.75	2.33	0.96	0.63	1.15		0.73	2.84
	1.17	0.97	6.50	1.12	0.89	1.21		1.02	1.77
	1.08	0.87		1.17	0.99				
	0.87	0.82		0.95	0.83				
	1.34	0.62		1.26	0.57				
		1.03			0.86				
2	1.08	1.25	1.71	1.04	1.08	0.91	0.79	1.41	0.89
	1.02	0.98	1.98	1.19	0.94	1.46	1.08	0.87	2.54
	1.07	0.84	1.85	0.99	0.84	1.32	0.77	0.94	1.76
	0.87	1.09	2.06	1.06	1.12	1.34	1.10	1.14	2.15
	0.90	1.00	1.97	0.91	1.23	1.14	0.65	0.97	2.55
	0.95	0.97	2.13	0.95	1.05	1.31	0.75	1.16	1.37
	1.21	1.03	2.20	1.04	0.85	1.05	1.07	0.89	1.37
	0.92	0.99	1.89	1.05	0.97	0.90	1.00	1.00	1.58
	1.10	1.12	2.15	1.43	0.92	1.78	1.47	1.13	2.70
	1.10	0.95	1.75	0.74	1.15	1.01	0.78	1.15	2.39
	0.95	1.03	2.06	1.14	0.86	1.31	1.17	0.83	1.57
	0.98	1.13	1.59	0.84	1.01	1.00	1.30	0.90	2.47
	0.95	1.32	1.99	0.76	1.02	1.48	1.09	1.13	1.76
	1.07	1.00	1.31	0.95	0.97	0.88	0.98	1.02	1.31
	0.84	0.99	1.72	1.10	0.94	1.12	0.93	1.03	1.91
	0.92	0.88		0.93	1.06		0.78	0.94	
	1.06			0.87			1.28		
**MV**	**1.00**	**0.97**	**2.12**	**1.00**	**0.91**	**1.20**	**1.00**	**0.99**	**1.90**
**SD**	**0.14**	**0.15**	**1.03**	**0.17**	**0.17**	**0.22**	**0.21**	**0.17**	**0.51**
**n**	**25**	**25**	**20**	**25**	**25**	**20**	**20**	**21**	**20**

CFP/GFP fluorescence intensity ratios in 5 days old *Arabidopsis* roots of F1 crosses (PLT3::CFP × 35S::WOX5-GFP-GR) without induction (-DEX), or induction with dexamethasone (+DEX) for 4 or 21 h. The data was normalized to the not induced roots (-DEX) per analyzed region of interest (ROI). MV = mean value, SD = standard deviation, n = number of analyzed roots (biological replicates) from two technical replicates.

Appendix Table S3 Intensity values of transcriptional and translational FP-tagged *PLT3* expression experiments in *Col-0* and *wox5* related to Figure 1

**Technical replicate**	**root #**	**pPLT3::erCFP fluorescence intensity [%]**		**pPLT3::PLT3-YFP fluorescence intensity [%]**	
		Col-0	***wox5***	***Col-0***	***wox5***
**1**	**1**	84	65	96	69
	**2**	108	67	121	31
	**3**	81	78	73	72
	**4**	93	48	68	64
	**5**	101	46	68	38
	**6**	95	65	95	109
	**7**	113	52	109	75
	**8**	128	53	72	39
	**9**	82	58	78	79
	**10**	71		127	30
	**11**	105		164	63
	**12**	139		84	39
	**13**			145	66
	**14**				55
	**15**				56
	**16**				99
**2**	**1**	87	57	104	78
	**2**	94	74	84	78
	**3**	91	62	62	76
	**4**	82	68	102	49
	**5**	111	79	126	43
	**6**	112	41	78	128
	**7**	102	39	82	101
	**8**	120	41	83	86
	**9**	144	58	139	98
	**10**	79	57	137	96
	**11**	121	57	104	71
	**12**	58	86		
	**13**		16		
	**14**		58		
	**15**		76		
	**16**		48		
	**17**		53		
	**18**		38		
	**MV**	**100**	**57**	**100**	**70**
	**SD**	**21**	**15**	**27**	**25**
	**n**	**24**	**27**	**24**	**27**

Number of roots (n) (biological replicates) from two independent technical replicates. MV = mean value, SD = standard deviation, n = number of analyzed roots.

Appendix Table S4: Quantification of transcriptional mVenus-tagged *WOX5* expression in the QC region of *Col* and *plt2, plt3* related to Figure 2

Technical replicate	root #	number of cells with *WOX5* expression		lateral area of *WOX5* expression in the root tip [µm²]	
		Col-0	*plt2, plt3*	*Col-0*	*plt2, plt3*
**1**	**1**	7	14	432.03	719.28
	**2**	4	9	345.88	886.24
	**3**	7	13	393.67	861.28
	**4**	7	13	400.62	882.48
	**5**	8	12	346.86	548.73
	**6**	6	11	430.11	736.44
	**7**	8	14	467.05	793.77
	**8**	6	12	512.33	693.18
	**9**	7	13	444.99	948.22
	**10**	9	14	474.60	747.27
**2**	**1**	9	11	388.87	725.36
	**2**	9	12	456.44	708.46
	**3**		11		479.29
	**4**		10		832.45
	**5**		14		793.67
	**6**		13		742.52
	**7**		12		1017.338
	**8**		13		807.87
	**9**		12		965.16
	**10**		11		730.23
	**11**		11		703.79
**3**	**1**	9	11	562.83	479.18
	**2**	10	9	414.58	738.43
	**3**	10	10	460.27	655.29
	**4**	9	12	429.66	555.08
	**5**		12		751.29
	**6**		10		606.65
	**7**		13		785.02
	**8**		8		640.39
	**9**		8		653.3
	**10**		11		911.77
	**11**		13		579.41
	**MV**	**7.81**	**11.63**	**435.05**	**739.96**
	**SD**	**1.59**	**1.65**	**54.38**	**132.08**
	**n**	**16**	**32**	**16**	**32**

Number of roots (n) (biological replicates) from three technical replicates. MV = mean value, SD = standard deviation, n = number of analyzed roots.

Appendix Table S5: Average QC and CSC phenotypes related to Figures 3 and 4

**Genotype**	**average QC cell-divisions per root (± SD)**	**average CSC layers per root (± SD)**	**number of analyzed roots (n)**
Col-0	0.34 ± 0.59	0.99 ± 0.59	146
plt2	0.53 ± 0.64	0.82 ± 0.72	77
plt3	0.61 ± 0.64	0.85 ± 0.69	121
plt2, plt3	0.81 ± 0.88	0.84 ± 0.82	105
wox5	1.87 ± 0.98	0.23 ± 0.44	118
wox5, plt3	2.11 ± 0.98	0.31 ± 0.51	87
wox5, plt2, plt3	2.83 ± 1.04	0.24 ± 0.46	98

n = number of analyzed roots (biological replicates) from 2-5 technical replicates per genotype. SD = standard deviation.

Appendix Table S6: Percentage of periclinal cell divisions in the QC shown in Figure EV1

**Genotype**	**periclinal QC cell divisions [%]**	**number of analyzed roots (n)**
Col	3	146
plt2	21	77
plt3	21	121
plt2, plt3	53	105
wox5	41	118
wox5, plt3	43	87
wox5, plt2, plt3	53	98

n = number of analyzed roots (biological replicates) from 2 - 5 technical replicates.

Appendix Table S7: Average QC and CSC phenotypes of rescue experiments shown in Figure EV2

Genotype	average QC cell-divisions per root (± SD)	average CSC layers per root (± SD)	number of analyzed roots (n)
Col	0.58 ± 0.79	0.89 ± 0.60	106
wox5	1.77 ± 1.12	0.33 ± 0.54	30
wox5 pWOX5::WOX5-mV	0.43 ± 0.66	0.88 ± 0.65	51
plt2	1.08 ± 0.94	0.85 ± 0.62	39
plt3	1.13 ± 1.03	0.55 ± 0.59	40
plt2, plt3	1.26 ± 1.06	0.55 ± 0.74	65
plt3 pPLT3::PLT3-mV	0.95 ± 1.01	0.88 ± 0.62	104
plt2, plt3 pPLT3::PLT3-mV	1.40 ± 1.05	0.66 ± 0.62	62
plt2, plt3 pPLT3:: **PLT3ΔPrD-mV**	1.34 ± 1.09	0.48 ± 0.62	44

Number of analysed roots (n) (biological replicates) is indicated for each genotype and results from 2 - 4 technical replicates. SD = standard deviation.

Appendix Table S8: Quantification of NBs in PLT1-4 related to Appendix Figure S3

Technical replicate	nucleus #	PLT1	PLT2	PLT3	PLT4
**1**	**1**	5.64	8.11	13.86	4.82
	**2**	5.68	5.06	13.31	15.26
	**3**	13.63	9.60	34.40	9.11
	**4**	23.89	13.55	33.85	11.36
	**5**	25.22	12.10	61.25	12.11
	**6**	14.13	8.03	23.11	6.52
	**7**	30.59	8.94	30.32	6.94
	**8**	17.12	22.47	16.79	10.45
	**9**	17.13	10.48	37.59	2.78
	**10**		12.41	32.11	6.73
	**11**		20.23	27.15	17.17
	**12**		7.90	53.54	5.93
	**13**		15.67	20.87	24.23
	**14**		22.27	19.78	
	**15**			19.97	
	**16**			20.31	
	**17**			18.52	
	**18**			42.59	
	**19**			61.43	
**2**	**1**	1.59	3.97	16.20	11.04
	**2**	33.53	3.74	39.37	2.19
	**3**	0.92	1.07	60.04	7.55
	**4**	16.51	8.77	41.97	4.11
	**5**	4.77	4.17	48.03	4.69
	**6**	4.27	3.24	107.30	5.06
	**7**	6.39	3.09	27.30	4.88
	**8**	9.66	14.48	66.67	2.09
	**9**	8.32	24.65	60.15	4.20
	**10**	4.20	6.65	42.76	1.60
	**11**	2.75	21.56	41.00	5.97
	**12**	11.21		27.03	5.11
	**13**			33.77	
	**14**			16.96	
	**15**			23.62	
	**16**			22.33	
	**MV**	**12.25**	**10.89**	**35.86**	**7.68**
	**SD**	**9.32**	**6.77**	**19.50**	**5.18**
	**n**	**21**	**25**	**35**	**25**

Number of NBs per 500 μm^3 nuclear volume. MV = mean value, SD = standard deviation, n = number of analyzed nuclei (biological replicates) from two technical replicates.

Appendix Table S9: FLIM results of subnuclear data analysis in WOX5-mV and PLT3-mCh co-expressing *N. benthamiana* epidermal cells related to Figure 7K

Technical replicate	measurement #	WOX5-mVenus fluorescence lifetime [ns]	
		bodies-only	nucleoplasm
1	WOX5-mV PLT3-mCh_7	2.24	2.76
	WOX5-mV PLT3-mCh_15	2.19	2.85
2	WOX5-mV PLT3-mCh_23	2.60	2.92
	WOX5-mV PLT3-mCh_25	2.55	2.88
3	WOX5-mV PLT3-mCh_4	2.56	2.89
4	WOX5-mV PLT3-mCh_1	2.50	2.84
	WOX5-mV PLT3-mCh_9	2.50	2.69
**MV**		**2.45**	**2.83**
**SD**		**0.15**	**0.07**
**n**		**7**	**7**

Number of analyzed nuclei (n) (biological replicates) from four technical replicates. MV = mean value, SD = standard deviation.

Appendix Table S10: List of cloning primers

cloning system	gene ID	alias	primer name	orientation	Sequence 5´→ 3´
GreenGate			**Promoter modules**		
	AT3G11260	pWOX5	RD_GreenGate pWOX5 F	F	AAAGGTCTCAACCTAAAGACTTTTATCTACCA ACTTCAAAAG
			RD_GreenGate pWOX5 R	R	AAAGGTCTCATGTTCGTTCAGATGTAAAG
			RD_GG pWOX5 BsaI a v2 F	F	AGAGACCAAATTATTTGGTTATATGGTAG
			RD_GG pWOX5 BsaI a v2 R	R	CTACCATATAACCAAAATAATTTGGTCTCT
			RD_GreenGate pWOX5 BsaI b F	F	ATTACGATGTGAGAGCGCCTTCAACTTT
			RD_GreenGate pWOX5 BsaI b R	R	AAAGTTGAAGGCGCTCTCACATCGTAAT
	AT5G10510	pPLT3	RD_GG pPLT3 F V2	F	AAAGGTCTCAACCTAATTTTAACGTATTCTTTC
			RD_GG pPLT3 R V2	R	AAAGGTCTCATGTTAAACTTTCTTATAAAAAC AATT
			**CDS modules**		
	AT3G11260	WOX5	RD_GreenGate WOX5 F	F	AAAGGTCTCAGGCTTAATGTCTTTCTCCGTG
			RD_GreenGate WOX5 Mitte R	R	GACGTCGTGGTGGTTTCTCGAATATATT
			RD_GreenGate WOX5 Mitte F	F	AATATATTCGAGAAACCACCACGACGTC
			RD_GreenGate WOX5 R	R	AAAGGTCTCACTGAAAGAAAGCTTAATCG
	AT5G10510	PLT3	RD_GreenGate PLT3 F	F	AAAGGTCTCAGGCTTAATGGAGATGTTGAG
			RD_GreenGate PLT3 R	R	AAAGGTCTCACTGAGTAAGACTGATTAGGC
		PLT3ΔPrD	RD_GreenGate PLT3 F	F	AAAGGTCTCAGGCTTAATGGAGATGTTGAG
			RD_PLT3ΔPrD1 CDS1 R	R	CCAGCTGCAACACCAAGTGACAAAG
			RD_PLT3ΔPrD1 linker F	F	CTTGGTGTTGCAGCTGGTGCTG
			RD_PLT3ΔPrD1 linker R	R	GTCTTCTCTGCTCCTGCGGCAG
			RD_PLT3ΔPrD1 CDS2 F	F	CGCAGGAGCAGAGAAGACAGATTCTG
			RD_GG PLT3ΔPrDs R	R	AAAGGTCTCACTGAGTGAAGTTGATGATGAC
	AT5G17430	PLT4	RD_GreenGate BBM F	F	AAAGGTCTCAGGCTTAATGAACTCGATGAAT
			RD_GreenGate BBM R	R	AAAGGTCTCACTGAAGTGTCGTTCCAAAC
			RD_GreenGate BBM Mitte F	F	ATTTACAATACCAACGAAACCGTTGTAGAT
			RD_GreenGate BBM Mitte R	R	ATCTACAACGGTTTCGTTGGTATTGTAAAT
Gateway	AT3G11260	WOX5	YS_WOX5 F CACC	F	CACCATGTCTTTCTCCGTGAAAGGTCGAAGCTT ACG
			YS_WOX5 R -stop	R	AAGAAAGCTTAATCGAAGATCTAATGGC
	AT3G20840	PLT1	YS_PLT1 F CACC	F	CACCATGAATTCTAACAACTGGCTTGGCT
			YS_PLT1 r -stop	R	CTCATTCCACATAGTGAAAACACCACCAGGG
	AT1G51190	PLT2	YS_PLT2 F CACC	F	CACCATGAATTCTAACAACTGGCTCGCGTTCCC TCT
			YS_PLT2 R -stop	R	TTCATTCCACATCGTGAAAACACCTCCT
	AT5G10510	PLT3	YS_PLT3 F CACC	F	CACCATGGAGATGTTGAGGTCATCTGATCAGT CTCA
			YS_PLT3 R -stop	R	GTAAGACTGATTAGGCCAGAGGAAG
		PLT3ΔQ	YS_PLT3 F CACC	F	CACCATGGAGATGTTGAGGTCATCTGATCAGT CTCA
			RD_PLT3 Seg1 R	R	GAGATGAGAAATGGTGAAGTTGATGATGAC
			RD_PLT3 Seg2 F	F	CTTCACCATTTCTCATCTCCTAATCACAGTAGC
			RD_PLT3 Seg2 R	R	GAAGAAGTTGTGGTGGTGGTAAAGAGCAG
			RD_PLT3 Seg3 F	F	CACCACCACAACTTCTTCCAGCATTTTCC
			YS_PLT3 R -stop	R	GTAAGACTGATTAGGCCAGAGGAAG

Appendix Table S11: List of expression vectors created in this study

cloning	constructs	promoter	N-tag	CDS	C-tag	term-inator	plant sel. marker	destination Vector	bacterial sel.	plasmid ID
		module A	module B	module C	module D	module E	module F	module Z		
GreenGate	**pWOX5:: mVenus-NLS**	WOX5 promoter	Ω-element (pGGB002)	mVenus	linker-NLS (pGGD007)	tUBQ10 (pGGE009)	BASTA (pGGF002)	pGGZ001	Spec	pVS10
	**pWOX5:: WOX5-mV**	WOX5 promoter	Ω-element (pGGB002)	WOX5	mVenus	tUBQ10 (pGGE009)	Hyg (pGGF005)	pGGZ001	Spec	pRD48
	**pPLT3:: PLT3-mV**	PLT3 promoter	Ω-element (pGGB002)	PLT3	mVenus	tUBQ10 (pGGE009)	Hyg (pGGF005)	pGGZ001	Spec	pRD73
	**pPLT3:: PLT3-mCh**	PLT3 promoter	Ω-element (pGGB002)	PLT3	mCherry	tUBQ10 (pGGE009)	-	pGGM000	Kan	pRD83
	**pWOX5:: WOX5-mV**	WOX5 promoter	Ω-element (pGGB002)	WOX5	mVenus	tUBQ10 (pGGE009)	Hyg (pGGF005)	pGGN000	Kan	pRD84
	**pPLT3::PLT3-mCh pWOX5::WOX5-mV**	pRD83 + pRD84						pGGZ001	Spec	pRD89
	**pPLT3:: PLT3ΔPrD-mV**	PLT3 promoter	Ω-element (pGGB002)	PLT3 ΔPrD	mVenus	tUBQ10 (pGGE009)	Hyg (pGGF005)	pGGZ001	Spec	pRD125
	**inducible PLT3ΔPrD-mV**	Ubi-XVE oLexA-35S	Ω-element (pGGB002)	PLT3 ΔPrD	mVenus	tUBQ10 (pGGE009)	Hyg (pGGF005)	pGGZ001	Spec	pRD106
	**inducible PLT3ΔPrD-mCh**	Ubi-XVE oLexA-35S	Ω-element (pGGB002)	PLT3 ΔPrD	mCherry	tUBQ10 (pGGE009)	Hyg (pGGF005)	pGGZ001	Spec	pRD138
	**inducible PLT2-mV**	Ubi-XVE oLexA-35S	Ω-element (pGGB002)	PLT2	mVenus	tUBQ10 (pGGE009)	Hyg (pGGF005)	pGGZ001	Spec	pRD102
	**inducible PLT4-mV**	Ubi-XVE oLexA-35S	Ω-element (pGGB002)	PLT4	mVenus	tUBQ10 (pGGE009)	Hyg (pGGF005)	pGGZ001	Spec	pRD150
	**inducible PLT4-mCh**	Ubi-XVE oLexA-35S	Ω-element (pGGB002)	PLT4	mCherry	tUBQ10 (pGGE009)	Hyg (pGGF005)	pGGZ001	Spec	pRD151
Gateway	**inducible WOX5-mV**	Ubi-XVE oLexA-35S	-	WOX5	mVenus	T3A	Hyg	pRD04	Spec	pRD26
	**inducible WOX5-mCh**	Ubi-XVE oLexA-35S	-	WOX5	mCherry	T3A	Hyg	pABind mCherry	Spec	pFB02
	**inducible PLT1-mV**	Ubi-XVE oLexA-35S	-	PLT1	mVenus	T3A	Hyg	pRD04	Spec	pRD56
	**inducible PLT1-mCh**	Ubi-XVE oLexA-35S	-	PLT1	mCherry	T3A	Hyg	pABind mCherry	Spec	pRD144
	**inducible PLT2-mCh**	Ubi-XVE oLexA-35S	-	PLT2	mCherry	T3A	Hyg	pABind mCherry	Spec	pRD147
	**inducible PLT3-mV**	Ubi-XVE oLexA-35S	-	PLT3	mVenus	T3A	Hyg	pRD04	Spec	pRD25
	**inducible PLT3-mCh**	Ubi-XVE oLexA-35S	-	PLT3	mCherry	T3A	Hyg	pABind mCherry	Spec	pFB06
	**inducible PLT3ΔQ-mV**	Ubi-XVE oLexA-35S	-	PLT3 ΔQ	mVenus	T3A	Hyg	pRD04	Spec	pRD57
	**inducible PLT3ΔQ-mCh**	Ubi-XVE oLexA-35S	-	PLT3 ΔQ	mCherry	T3A	Hyg	pABind mCherry	Spec	pRD81

Appendix Table S12: ***Arabidopsis*** **mutants and transgenic lines used in this study**

gene ID	alias	reference
AT3G11260	*wox5-1*	SALK038262 (ABRC)
AT5G10510	*plt3-1*	Galinha *et al,* 2007
AT1G51190	*plt2*	SALK_128164 (ABRC)
AT1G51190, AT5G10510	*plt2, plt3*	this study, crossing of *plt2* and *plt3-1*
AT3G11260, AT5G10510	*wox5, plt3*	this study, crossing of *wox5-1* and *plt3-1*
AT3G11260, AT1G51190, AT5G10510	*wox5, plt2, plt3*	this study, crossing of *plt2, plt3* and *wox5, plt3*
AT5G10510	pPLT3::erCFP (*Col-0*)	(Galinha *et al,* 2007)
AT5G10510	pPLT3::erCFP (*wox5-1*)	this study, crossing of pPLT3::erCFP (*Col-0*) and *wox5-1*
AT5G10510	pPLT3::PLT3-YFP (*Col-0*)	(Galinha *et al,* 2007)
AT5G10510	pPLT3::PLT3-YFP (*wox5-1*)	this study, crossing of pPLT3::PLT3-YFP (*Col-0*) and *wox5-1*
AT5G10510	pPLT3::PLT3-mVenus (*plt2, plt3*)	this study
AT5G10510	pPLT3::PLT3ΔPrD-mVenus (*plt2, plt3*)	this study
AT5G10510	estradiol inducible PLT3-mVenus (*Col-0*)	this study
AT3G11260	pWOX5::mVenus-NLS (*Col-0*)	this study
AT3G11260	pWOX5::mVenus-NLS (*plt2*)	this study
AT3G11260	pWOX5::mVenus-NLS (*plt3-1*)	this study
AT3G11260	pWOX5::mVenus-NLS (*plt2, plt3*)	this study
AT3G11260	35S::WOX5-GFP-GR	Berckmans *et al,* 2020
AT3G11260 AT5G10510	35S::WOX5-GFP-GR x pPLT3::erCFP	this study
AT3G11260	35S::WOX5-GR	Sarkar *et al,* 2007

Appendix Table S13: List of genotyping and qPCR primers

gene ID	alias	primer name	orien-tation	Sequence 5´→ 3´	used for
AT3G11260	*wox5-1*	GK_WOX5 F	F	AAACAGTTGAGGACTTTACATCTGA	genotyping
		WOX5 R	R	CGGATAATATGTCATAATTCAAAAT	
AT5G10510	*plt3-1*	GK_PLT3L	F	TTGTGATTTGCCATTGACTAAAGGT	genotyping
		GK_PLT3R	R	GAAAACAGTCCAATGGTCTCACATC	
AT1G51190	*plt2*	RD_plt2 SALK128164 neu L	F	GCTTAAATAGATATATGGTCATGCTTATATTC	genotyping
		RD_plt2 SALK128164 neu R	R	CAAGAAGACTCCAGCCGATC	
		SALK LBB1V2		AAACCAGCGTGGACCGCTTGCTGCAACTCT	
AT5G10510	PLT3	RTAIL6-8	F	GGGATAATAGCTGTAGGAGAGAAG	qPCR, Krizek, 2015
		RTAIL6-9	R	TCGAGCTGCCTTATCTTCTTTG	
AT3G18780	ACTIN	ACTIN F	F	CCGCTCTTTCTTTCCAAGC	qPCR, Shimotohno *et al,* 2018
		ACTIN R	R	CCGGTACCATTGTCACACAC	

References

Berckmans B, Kirschner G, Gerlitz N, Stadler R, Simon R (2020) CLE40 Signaling Regulates Root Stem Cell Fate. *Plant Physiol* 182: 1776–1792

Galinha C, Hofhuis H, Luijten M, Willemsen V, Blilou I, Heidstra R, Scheres B (2007) PLETHORA proteins as dose-dependent master regulators of Arabidopsis root development. *Nature* 449: 1053–1057

Krizek BA (2015) Intronic sequences are required for AINTEGUMENTA-LIKE6 expression in Arabidopsis flowers. *BMC Res Notes* 8: 556

Livak KJ, Schmittgen TD (2001) Analysis of relative gene expression data using real-time quantitative PCR and the 2(-Delta Delta C(T)) Method. *Methods* 25: 402–408

Sarkar AK, Luijten M, Miyashima S, Lenhard M, Hashimoto T, Nakajima K, Scheres B, Heidstra R, Laux T (2007) Conserved factors regulate signalling in Arabidopsis thaliana shoot and root stem cell organizers. *Nature* 446: 811–814

Shimotohno A, Heidstra R, Blilou I, Scheres B (2018) Root stem cell niche organizer specification by molecular convergence of PLETHORA and SCARECROW transcription factor modules. *Genes Dev* 32: 1085–1100
