# Supplementary material for: PLETHORA‐WOX5 interaction and subnuclear localization control Arabidopsis root stem cell maintenance
Source: EMBO Rep. 2022 Apr 4;23(6):e54105. doi: 10.15252/embr.202154105 (PMC9171415; doi:10.15252/embr.202154105)
Supplement: Supplementary file 2 — Expanded View Figures PDF [file EMBR-23-e54105-s003.pdf]

Expanded View Figures

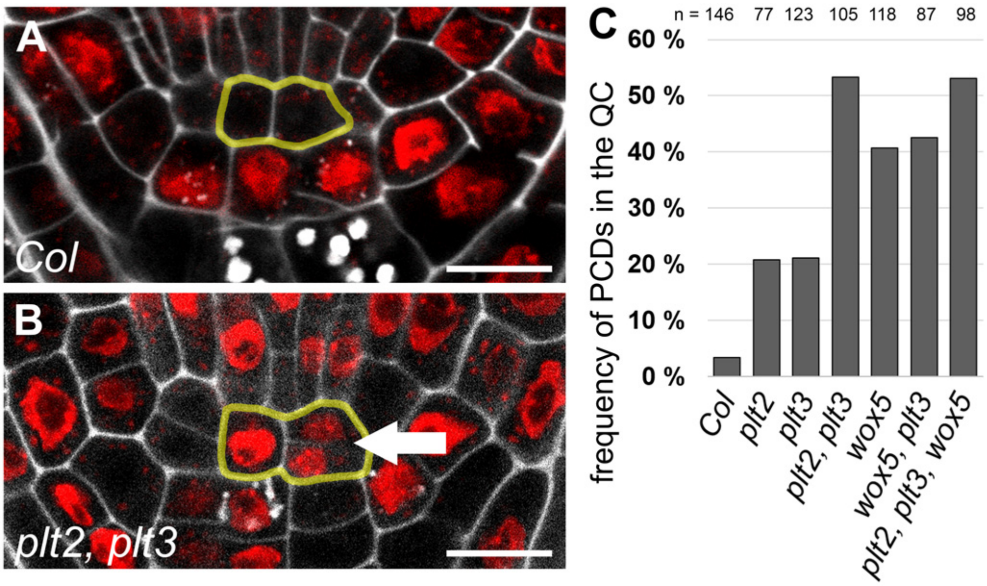

Figure EV1. *plt* and *wox5* mutants show more periclinal cell divisions in the QC.

A Representative figure of an *Arabidopsis* wild-type root SCN staining. QC cells are outlined in yellow. Scale bars represent 10 µm.

B Representative figure of an *Arabidopsis plt2, plt3* double mutant root SCN staining showing a periclinal cell division (PCD) in the QC (arrow). QC cells are outlined in yellow. Scale bars represent 10 µm.

C Analysis of the PCD phenotype. The frequency of roots (in percent) showing at least one PCD in the QC is plotted as a bar graph. Number of analyzed roots (*n*) (biological replicates) is indicated for each genotype and results from 2 to 5 technical replicates. PCD = periclinal cell division.

Figure EV2. Mutant rescue experiments.

SCN stainings were performed in *Arabidopsis thaliana* seedlings in the indicated single and double mutant backgrounds expressing either WOX5-mV, PLT3-mV or PLT3ΔPrD-mV driven by their endogenous promoters as well as in *Col* wild type.

A–I The combined results of the SCN staining are shown as 2D plots. Number of CSC layers is shown on the y axis and the QC division phenotype is shown on the x-axis. The darker the color, the more roots show the respective phenotype (see color gradient on the right indicating the frequencies).

J, K Analyses of the SCN staining for CSC layer (J) or QC division (K) phenotypes. The frequencies of roots showing 0–3 CSC layers, or 0–4 dividing QC cells are plotted as bar graphs. Number of analyzed roots (*n*) (biological replicates) is indicated for each genotype and results from 2 to 4 technical replicates. EdU = 5-ethynyl-2′-deoxyuridine; CSC = columella stem cell; QC = quiescent center; W5 = WOX5, P3 = PLT3.

Source data are available online for this figure.

© 2022 The Authors

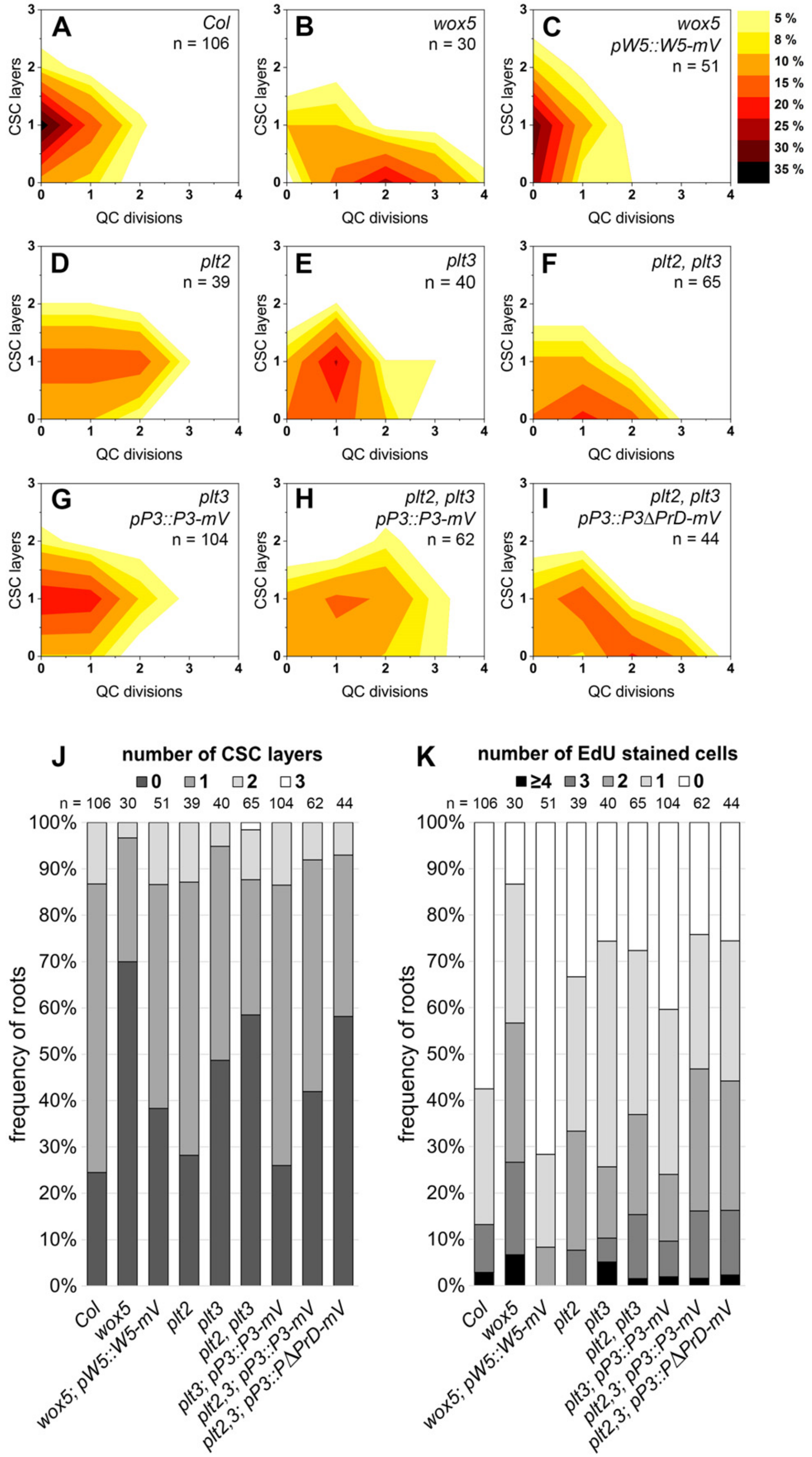

Figure EV2.

© 2022 The Authors

Figure EV3. Subnuclear localization and PrD prediction of WOX5, PLT1, PLT2, PLT3, and PLT4.

A, D, G, J, M Representative images of (sub-)nuclear localization of WOX5-mV (A), PLT1-mV(D), PLT2-mV (G), PLT3-mV (J) and PLT4-mV (m) in transiently expressing *N. benthamina* epidermal cells. Scale bars represent 5 μm.

B, E, H, K, N Schematic representation of WOX5 (B), PLT1 (E) PLT2 (H), PLT3 (K), and PLT4 (N) protein domains. The areas in red are predicted prion-like domains (PrDs), analyzed using the PLAAC prediction tool. Yellow areas are polyQ stretches (defined as more than three Qs in a row) in the PLT3 and PLT4 amino acid sequence.

C, F, I, L, O Protein sequences of WOX5 (C) PLT1 (F), PLT2 (I), PLT3 (L), and PLT4 (O). The red highlighted sequences are the predicted prion-like domains (PrDs). mV = mVenus fluorescent protein; PrD = prion-like domain; EAR = Ethylene-responsive binding factor-associated repression domain; WUS = WUSCHEL box; AP2 = APETALA2 domain; NLS = nuclear localization signal.

Source data are available online for this figure.

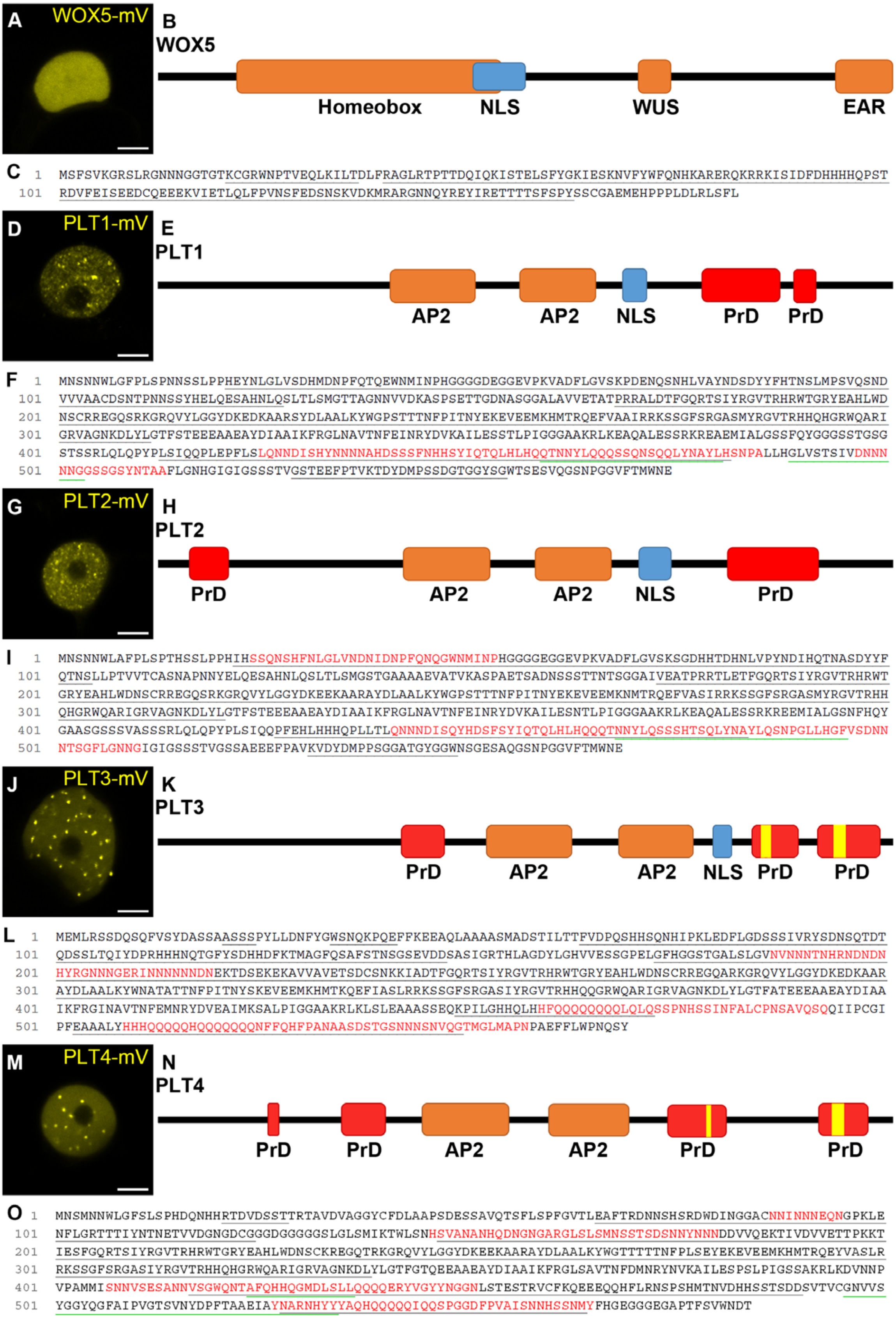

Figure EV3.

 © 2022 The Authors

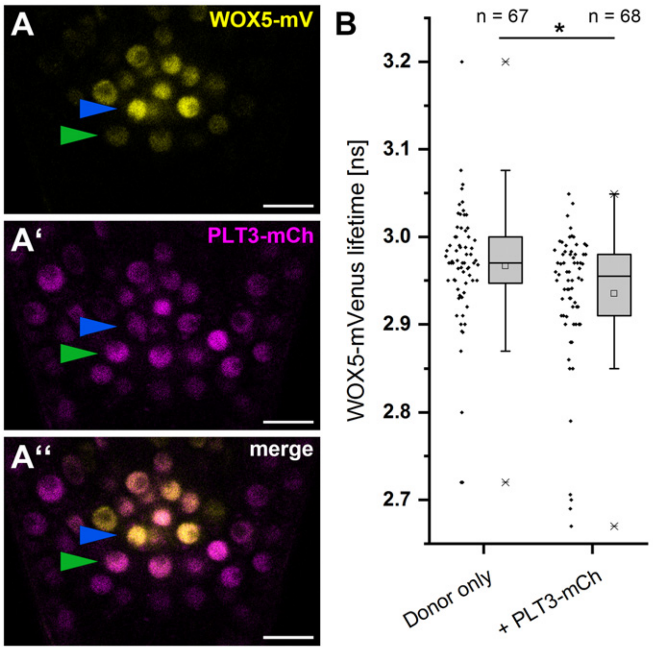

Figure EV4. PLT3-WOX5 interaction in the *Arabidopsis* root.

A–A′ Representative image of the SCN in a lateral root of an *Arabidopsis* reporter line expressing WOX5-mV (A) and PLT3-mCh (A′) in *Col-0* background driven by their respective endogenous promoters. The TFs localize to overlapping domains (A″). Blue arrowheads mark QC cells, green arrowheads mark CSCs. Scalebars represent 10 μm.

B Fluorescence Lifetime Imaging (FLIM) results of experiments performed in the SCN of lateral *Col-0* roots expressing either only WOX5-mV (donor-only) or both WOX5-mV and PLT3-mCh driven by their respective endogenous promoters. Number of analyzed roots (*n*) (biological replicates) is indicated for each genotype and results from 4 technical replicates. Donor fluorescence lifetimes in ns are summarized in combined scatter and box plots. Box = 25–75% of percentile, whisker = 1.5 interquartile range, — = median, □ = mean value, X = minimum/maximum value. The Kruskal–Wallis ANOVA with subsequent Dunn's test was used to test for statistical significance. Asterisk indicates statistical significance ($\alpha = 0.01$).
mV = mVenus; mCh = mCherry; SCN = stem cell niche.

© 2022 The Authors

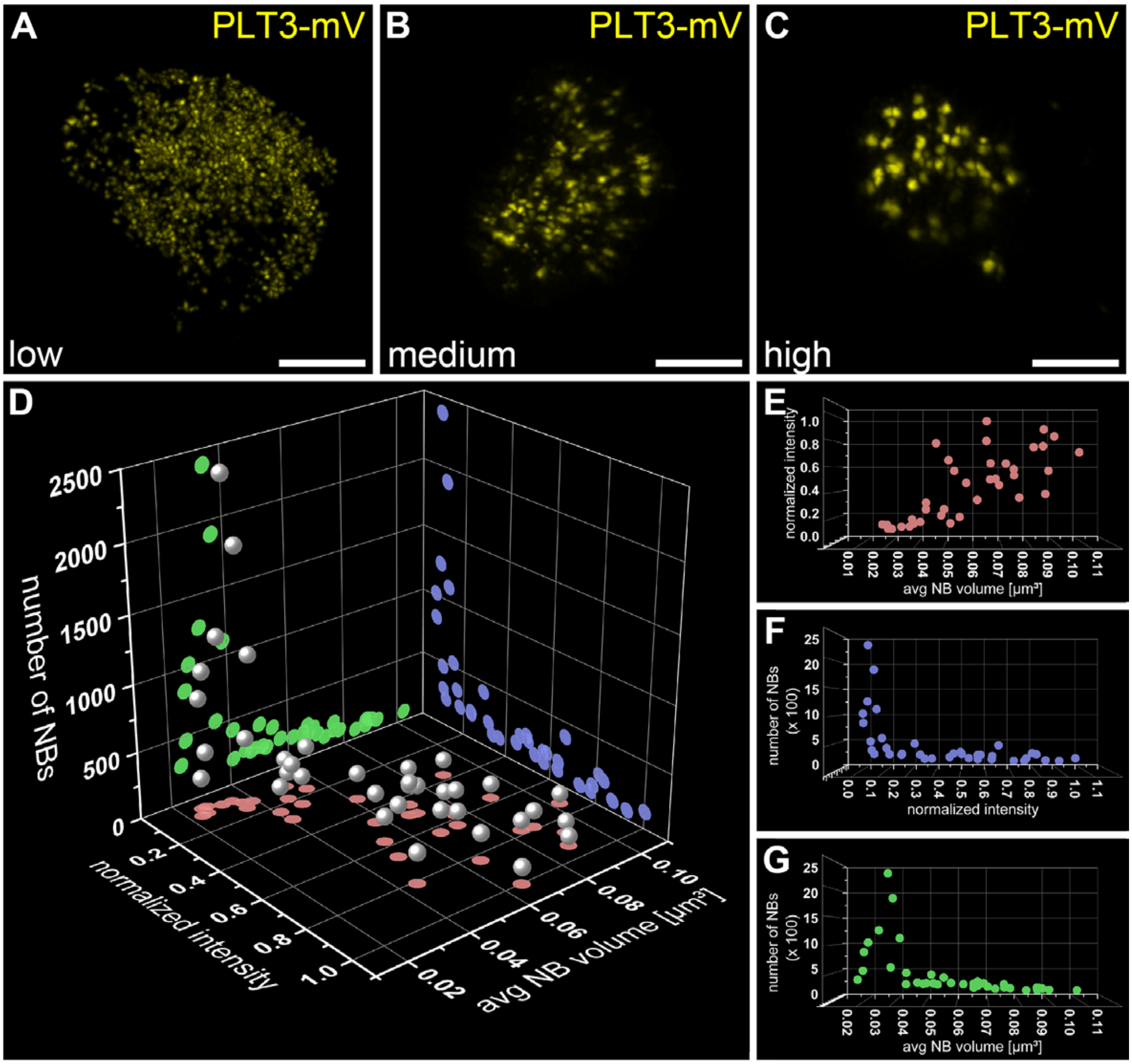

Figure EV5. Concentration dependency of PLT3 nuclear body formation.

A–C Representative images of low (A), medium (B), and high (C) PLT3-mVenus expressing nuclei in transiently expressing *N. benthamiana* leaf epidermal cells are shown. Scale bars represent 5 µm.

D–G Analyses of intensities, numbers, and average volume of PLT3 NBs in individual nuclei, $n = 37$ (biological replicates).

 © 2022 The Authors
