# Supplementary material for: PLETHORA‐WOX5 interaction and subnuclear localization control Arabidopsis root stem cell maintenance
Source: EMBO Rep. 2022 Apr 4;23(6):e54105. doi: 10.15252/embr.202154105 (PMC9171415; doi:10.15252/embr.202154105)
Supplement: Supplementary file 3 — Movie EV1 [file EMBR-23-e54105-s006.zip › movie EV1 legend.docx]

**Movie EV1 - Dynamic formation of nuclear bodies in a PLT3-mVenus expressing LRP.** The video shows a developing lateral root primordium in an *Arabidopsis thaliana* plant expressing mVenus tagged PLT3 driven by its endogenous promoter (pPLT3::PLT3 mVenus) over 18 hours. Scale bar represents 25 µm.
