# Supplementary material for: PLETHORA‐WOX5 interaction and subnuclear localization control Arabidopsis root stem cell maintenance
Source: EMBO Rep. 2022 Apr 4;23(6):e54105. doi: 10.15252/embr.202154105 (PMC9171415; doi:10.15252/embr.202154105)
Supplement: Supplementary file 8 [file EMBR-23-e54105-s001.pdf]

EMBO reports

PLETHORA-WOX5 interaction and subnuclear localisation control *Arabidopsis* root stem cell maintenance

Rebecca Burkart, Vivien Strotmann, Gwendolyn Kirschner, Abdullah Akinci, Laura Czempik, Anika Dolata, Alexis Maizel, Stefanie Weidtkamp-Peters, and Yvonne Stahl

DOI: 10.15252/embr.202154105

Corresponding author(s): Yvonne Stahl (Yvonne.Stahl@hhu.de)

Review Timeline:

Submission Date:	5th Oct 21
Editorial Decision:	3rd Nov 21
Revision Received:	24th Feb 22
Editorial Decision:	17th Mar 22
Revision Received:	17th Mar 22
Accepted:	18th Mar 22

Editor: Achim Breiling

Transaction Report:

(Note: With the exception of the correction of typographical or spelling errors that could be a source of ambiguity, letters and reports are not edited. Depending on transfer agreements, referee reports obtained elsewhere may or may not be included in this compilation. Referee reports are anonymous unless the Referee chooses to sign their reports.)

1st Editorial Decision **3rd Nov 2021**

Dear Prof. Stahl,

Thank you for the submission of your research manuscript to EMBO reports. We have now received the reports from the three referees that were asked to evaluate your study, which can be found at the end of this email.

As you will see, the referees think that these findings are of interest. However, they have several comments, concerns and suggestions, indicating that a major revision of the manuscript is necessary to allow publication of the study in EMBO reports. As the reports are below, and all their points need to be addressed, I will not detail them here.

Given the constructive referee comments, we would like to invite you to revise your manuscript with the understanding that all referee concerns must be addressed in the revised manuscript or in the detailed point-by-point response. Acceptance of your manuscript will depend on a positive outcome of a second round of review. It is EMBO reports policy to allow a single round of revision only and acceptance of the manuscript will therefore depend on the completeness of your responses included in the next, final version of the manuscript.

Revised manuscripts should be submitted within three months of a request for revision. Please contact me to discuss the revision should you need additional time.

When submitting your revised manuscript, please also carefully review the instructions that follow below.

PLEASE NOTE THAT upon resubmission revised manuscripts are subjected to an initial quality control prior to exposition to re-review. Upon failure in the initial quality control, the manuscripts are sent back to the authors, which may lead to delays. Frequent reasons for such a failure are the lack of the data availability section (please see below) and the presence of statistics based on n=2 (the authors are then asked to present scatter plots or provide more data points).

When submitting your revised manuscript, we will require:

1) a .docx formatted version of the final manuscript text (including legends for main figures, EV figures and tables), but without the figures included. Please make sure that changes are highlighted to be clearly visible. Figure legends should be compiled at the end of the manuscript text.

2) individual production quality figure files as .eps, .tif, .jpg (one file per figure), of main figures and EV figures. Please upload these as separate, individual files upon re-submission.

The Expanded View format, which will be displayed in the main HTML of the paper in a collapsible format, has replaced the Supplementary information. You can submit up to 5 images as Expanded View. Please follow the nomenclature Figure EV1, Figure EV2 etc. The figure legend for these should be included in the main manuscript document file in a section called Expanded View Figure Legends after the main Figure Legends section. Additional Supplementary material should be supplied as a single pdf file labeled Appendix. The Appendix should have page numbers and needs to include a table of content on the first page (with page numbers) and legends for all content. Please follow the nomenclature Appendix Figure Sx, Appendix Table Sx etc. throughout the text, and also label the figures and tables according to this nomenclature.

For more details, please refer to our guide to authors:
http://www.embopress.org/page/journal/14693178/authorguide#manuscriptpreparation

Please consult our guide for figure preparation:
http://wol-prod-cdn.literatumonline.com/pb-assets/embo-site/EMBOPress_Figure_Guidelines_061115-1561436025777.pdf

See also the guidelines for figure legend preparation:
https://www.embopress.org/page/journal/14693178/authorguide#figureformat

3) a .docx formatted letter INCLUDING the reviewers' reports and your detailed point-by-point responses to their comments. As part of the EMBO Press transparent editorial process, the point-by-point response is part of the Review Process File (RPF), which will be published alongside your paper.

4) a complete author checklist, which you can download from our author guidelines (https://www.embopress.org/page/journal/14693178/authorguide). Please insert page numbers in the checklist to indicate where the requested information can be found in the manuscript. The completed author checklist will also be part of the RPF.

Please also follow our guidelines for the use of living organisms, and the respective reporting guidelines:
http://www.embopress.org/page/journal/14693178/authorguide#livingorganisms

5) that primary datasets produced in this study (e.g. RNA-seq, ChIP-seq, structural and array data) are deposited in an appropriate public database. If no primary datasets have been deposited, please also state this a dedicated section (e.g. 'No primary datasets have been generated and deposited'), see below.

See also: http://embor.embopress.org/authorguide#datadeposition

Please remember to provide a reviewer password if the datasets are not yet public.

The accession numbers and database should be listed in a formal "Data Availability " section (placed after Materials & Methods) that follows the model below. This is now mandatory (like the COI statement). Please note that the Data Availability Section is restricted to new primary data that are part of this study.

Data availability

The datasets produced in this study are available in the following databases:

- RNA-Seq data: Gene Expression Omnibus GSE46843 (https://www.ncbi.nlm.nih.gov/geo/query/acc.cgi?acc=GSE46843)
- [data type]: [name of the resource] [accession number/identifier/doi] ([URL or identifiers.org/DATABASE:ACCESSION])

*** Note - All links should resolve to a page where the data can be accessed. ***

Moreover, I have these editorial requests:

6) We strongly encourage the publication of original source data with the aim of making primary data more accessible and transparent to the reader. The source data will be published in a separate source data file online along with the accepted manuscript and will be linked to the relevant figure. If you would like to use this opportunity, please submit the source data (for example scans of entire gels or blots, data points of graphs in an excel sheet, additional images, etc.) of your key experiments together with the revised manuscript. If you want to provide source data, please include size markers for scans of entire gels, label the scans with figure and panel number, and send one PDF file per figure.

7) Our journal encourages inclusion of *data citations in the reference list* to directly cite datasets that were re-used and obtained from public databases. Data citations in the article text are distinct from normal bibliographical citations and should directly link to the database records from which the data can be accessed. In the main text, data citations are formatted as follows: "Data ref: Smith et al, 2001" or "Data ref: NCBI Sequence Read Archive PRJNA342805, 2017". In the Reference list, data citations must be labeled with "[DATASET]". A data reference must provide the database name, accession number/identifiers and a resolvable link to the landing page from which the data can be accessed at the end of the reference. Further instructions are available at: http://www.embopress.org/page/journal/14693178/authorguide#referencesformat

8) Regarding data quantification and statistics, can you please specify, where applicable, the number "n" for how many independent experiments were performed, if these were biological or technical replicates, the bars and error bars (e.g. SEM, SD) and the test used to calculate p-values in the respective figure legends. Please provide statistical testing where applicable, and also add a paragraph detailing this to the methods section. See:
http://www.embopress.org/page/journal/14693178/authorguide#statisticalanalysis

9) Please also note our reference format:
http://www.embopress.org/page/journal/14693178/authorguide#referencesformat

10) Please present the results part in individual sections with sub-headings. This would render this part more comprehensible.

I look forward to seeing a revised version of your manuscript when it is ready. Please let me know if you have questions or comments regarding the revision.

Please use this link to submit your revision: https://embor.msubmit.net/cgi-bin/main.plex

Yours sincerely,

Achim Breiling
Editor
EMBO Reports

Referee #1:

In this interesting manuscript Burkart and colleagues investigate the interaction between PLT TFs and WOX5 in maintaining the

QC quiescence and stem cell activity. Utilizing genetic experiments combine with a mixed EdU/mPSPI staining they first show that those TFs cooperate to maintain columella stem cell activity. Differently in the QC those TFs act independently to maintain the quiescence of those cells. Via a set of molecular biology and biochemistry experiments the authors subsequently show that PLT3 differently from WOX5 forms nuclear bodies. They show that WOX5 and PLT3 physically interact and that nuclear bodies formation is important for this interaction. Via domain deletion experiments they show that a Prion like domain is responsible for the formation of PLT3 nuclear bodies and that those bodies are fundamental for the function of PLT3 protein.

To this reviewer the manuscript looks solid and reports novel findings. However some aspects look not so clear to me.

Major points:
1. In the first part of the manuscript the authors report the genetic interaction between WOX5 and PLT2 and 3 in the control of columella stem cells and QC quiescence. Despite they report solid experiments, the cause effect link on the QC and columella stem cell function of those TFs is missing. Indeed, the utilized genetic backgrounds have perturbed SCN since the embryogenesis, hence, those do not permit to understand whether the described phenotypes are due to the lack of the QC and SCN functioning only or to the activity of PLT3and WOX5 (e.g. lack of a functional QC leads cell non autonomously to CSC differentiation). I would suggest authors to utilize also inducible gain of function versions of those TFs to clarify this point.
2. PLT3 and PLT2 are expressed both in the QC and CSC. It is not clear to this reviewer whether the described phenotypes of plt3,plt2 derive from the activity of PLTs in the QC or in the SCN or both. As authors focus on the different functional interaction of WOX5 and PLTs in the QC and in the CSC, I would suggest authors to provide tissue specific evidence of this different function (e.g. QC/CSC expression of PLT3 in plt3 and wox5 mutants).
3. I do not understand why authors focus mostly on PLT3 and not other PLTs that are expressed in the QC in a similar fashion (PLT2, PLT1 etch...), despite to lower (?) extent. This point should be better explained.
4. L258-261- The authors nicely show that having PrD PLT3 forms nuclear bodies, and they show that other PLTs have the PrD domain but lack or show short Q rich domain. It is not clear to this reviewer whether the ability to form nuclear bodies is a characteristic only of PLT3 or also of the other PLTs. Authors should clarify this point.

Minor points:
L84-authors should report some examples of the several physiological reactions of NBs to enable readers to critically understand their findings.
Fig.2 C is out of focus and should be replaces.

Referee #2:

The manuscript by Stahl and co-workers re-investigates the PLT-WOX regulation with respect to mutual transcription regulation and confirms earlier findings that PLT confines WOX5 expression whereas WOX5 promotes PLT expression. This feedback loop is important for QC function and CSC maintenance. However, in terms of QC quiescence these genes appear to be working in parallel. These different mechanisms of action indicate the complex relation of PLT and WOX5 genes in the root stem cell niche. The authors show detailed analyses of the phenotypes in single and multiple mutants for support and correlate the CSC differentiation with increased QC divisions as a mechanism to replenish lost stem cells. The manuscript becomes interesting when the authors look carefully at the localization of the proteins inside the nucleus. Depending on the position of the cell in the developing organ (CSC or LRP) the PLT proteins localize in nuclear bodies and sequester also WOX5. This can explain the "acting together to maintain CSC homeostasis" that was derived from the phenotypic mutant analyses looking at CSC differentiation. The NB localization is coupled to the presence of so-called prion domains in the PLT proteins which leads the authors to propose a function for this localization as being a determinant factor for stem cell and future CC fate.

The experiments are performed with high quality incorporating detailed phenotyping, novel staining methods and high-tech interaction studies.

Below I will remark on the results described in this manuscript.

Ln89 and throughout the manuscript there is a mixed use of "CSC" and "distal stem cell". These are the same cell and maybe I missed it but it would be good to clearly state this somewhere in the beginning.
Ln143-153 and Fig 3. This section describes the CSC differentiation using the new staining method. First, it will help the reader if the images contain marks, e.g. arrowheads, that point out the QC or mutant QC and the CSC or mutant CSC. For example, it is not obvious where the QC is in fig 3H. Second, dividing cells stained through EDU accumulation containing little starch are scored as differentiated cells thereby disturbing the starch free CSC layer which is therefore scored as a differentiating CSC layer. This is of course a way of scoring but it also means that there are stem cells remaining even when some CSCs in the layer are differentiating. Some nuancing helps to make this clear. Even so, is there evidence that these EDU stained but starch containing CSCs cells are truly differentiating when they appear to be/have divided recently. Or is it a matter of definition. It will be good to make this clear.
Ln157, Fig1 should be Fig3
Ln158, I would change the "QC within 24 hours" into "QC within the 24 hours staining window"
Ln160, there is QC division frequency in the plt2plt3 double that is 57%. However, this double shows a duplicated QC according

to Fig2D. Therefor I wonder if only a single plane is used for counting the divisions and whether this plane represents the bottom layer of QC cells. The authors should make this clear.
Ln170, states "according to our hypothesis". I may have missed it but I did not find this hypothesis stated before, only that in line 124 the authors intend "to probe for their interdependency". Please rephrase.
Ln215, 245, 247, 261 and maybe at more places, Suppl fig 3 should be 4
Regarding Suppl Fig3, there are no arrowheads as indicated in the legend
Ln257-61 states that the PLT PrD domains are responsible for the recruitment of WOX5 to NBs. I have a few points that I like to raise here. First, I wonder if WOX5 is also recruited to the NB in LRPs. WOX5 is expressed already in stage II primordia and therefore it is possible to check for colocalization. Please test this. The advantage being that here the NB are seen often and pronounced according to the authors. It will provide additional relevance for the proposed interaction. Also, as mentioned in the next pages, if the co-localisation is observed it provides the authors with a better opportunity to test interaction with FLIM in vivo. Second, I am confused about the "most frequently" since the authors show that all PLTs are observed in NB in Suppl Fig4. How often do the authors actually see localization in NB in betha leaf cells for the different PLTs?
Ln283, Fig 7H should be I
Ln292, Fig 7I,J should be J,K
Ln293-301. Did the authors measure the interaction of WOX5 and PLT3 using the complemented mutants? Please state. And, earlier the authors show there are NBs observed in the root niche as shown in Fig5. Then why not cross lines in which the NBs are observed. And coming back to my earlier remark, in Fig5A there is clear NB in multiple cells of the LRP. Please try to measure interaction in these cells if WOX5 is colocalized to NBs here. And Suppl Fig4 should be 3 now.
Ln320-1 states the PrD containing the polyQ are required for NB formation and WOX5 complex formation. Still, the NB form in the PLT3 polyQ mutant but no PLT3-WOX5 interaction takes place indicating that the polyQ is required for interaction. However, also interaction of WOX5 to PLT1 and 2 is observed which do not have polyQ annotated. Possibly the polyQ deletion disturbs the PrD domain which is the reason that interaction does not take place? Possibly polyQ annotation is not straightforward. I feel that now there is emphasis on polyQ required for complex formation, e.g. ln379, whereas this may not be so straighforward. Please discuss.
Ln331-3. I feel this single summarizing sentence does not give credit to the complex relation between PLTs and WOX5 and the results that the authors add to this relation here. Please rephrase in few sentences.

Referee #3:

The manuscript by Burkart et al., presents interesting new findings about the role of well known plants specific transcription factors, PLTs and WOX5. Over the years both the factors have been studied in the context of root development and their role in regulating the maintenance of the stem cell niche is well established. So far, people in the field have been looking at these regulators like many other accessory transcription factors and thought of their predominant role as nuclear localized proteins. This study provokes new thinking and convincingly shows that there is a clear sub-nuclear localization in the columella stem cell niche. They further went ahead and showed that PLT3 interacts with WOX5 and gets recruited to nuclear bodies. This recruitment is important for maintenance of the columella stem cell niche, well supported by genetics. In general I like this part of the work. These transcription factors also regulate various regenerative responses in plants, the present work opens up the possibilities of investigating the importance of such subnuclear localization in other contexts of the development too.

I have the following points:

1- Regarding the transcriptional regulation of PLT3 by WOX5, is it direct or indirect? The authors should check whether PLT3 can be rapidly upregulated by induction of WOX5. They can use steroid inducible versions of WOX5-GR to address this. Or do they imply that transcription regulation of PLT3 by WOX5 is an indirect one? This is important as PLT1 and PLT3 were shown to directly regulate WOX5 transcription (Shimotohno et al, 2018).

2- What made the authors analyse plt2,plt3 double mutant? I can understand that based on predominant expression of PLT3 in distal stem cells they chose plt3, but why plt2 ? Why not plt1 mutant? In the result section it reads as an abrupt choice given the redundant role of PLT1, PLT2 and PLT3 in maintenance of the root stem cell niche.

3- Also, while interpreting the significance of regulation of WOX5 by PLTs and vice versa, they should take into account the fact that WOX5 is under direct transcriptional regulation of PLT. Now that they have a clearer picture of additional PLT-WOX5 interaction (both at the level of protein-protein interaction and protein-DNA interaction) they must provide a model for much deeper insights into the cumulative role of PLTs and WOX5.

4- Throughout the figures, I did not find "n" (total number of samples/root tips) taken for various reporter and mutant analysis. Please indicate that on the panels wherever it is applicable.

Dear Editor,

we would like to thank all three referees for taking the time to give us valuable and constructive comments on our manuscript. We have carefully revised our manuscript in response to these very helpful comments and addressed all questions raised by the referees.

We included the respective explanations to the main text and materials and methods and added new datasets (Appendix Tables S11 S12, and S13) for three new figures (Appendix Figures 1-3). Furthermore, we have updated ten existing figures (Figures 1-4, Figure 7, Figure 8, and EV Figures 1-4) as well as the related source data tables (Appendix Tables S4 – S6). We have highlighted these changes in the main text and appendix and added comments indicating which question was answered.

We have also added the requested editorial changes by providing sub-headings in the results section, updating the callout of figures and tables, adjusting the reference style and by providing information on statistics, which are also marked by track changes.

We think that, thanks to the referees, our revised manuscript is much improved, and we hope that the referees will be able to support its publication with EMBO reports.

Below you may find our point-by-point response to all raised questions can be found highlighted in blue.

With kind regards on behalf of all authors

Yvonne Stahl

Referee #1:

In this interesting manuscript Burkart and colleagues investigate the interaction between PLT TFs and WOX5 in maintaining the QC quiescence and stem cell activity. Utilizing genetic experiments combine with a mixed EdU/mPSPI staining they first show that those TFs cooperate to maintain columella stem cell activity. Differently in the QC those TFs act independently to maintain the quiescence of those cells. Via a set of molecular biology and biochemistry experiments the authors subsequently show that PLT3 differently from WOX5 forms nuclear bodies. They show that WOX5 and PLT3 physically interact and that nuclear bodies formation is important for this interaction. Via domain deletion experiments they show that a Prion like domain is responsible for the formation of PLT3 nuclear bodies and that those bodies are fundamental for the function of PLT3 protein.

To this reviewer the manuscript looks solid and reports novel findings. However some aspects look not so clear to me.

Major points:

Q1. In the first part of the manuscript the authors report the genetic interaction between WOX5 and PLT2 and 3 in the control of columella stem cells and QC quiescence. Despite they report solid experiments, the cause effect link on the QC and columella stem cell function of those TFs is missing. Indeed, the utilized genetic backgrounds have perturbed SCN since the embryogenesis, hence, those do not permit to understand whether the described phenotypes are due to the lack of the QC and SCN functioning only or to the activity of PLT3and WOX5 (e.g. lack of a functional QC leads cell non autonomously to CSC differentiation). I would suggest authors to utilize also inducible gain of function versions of those TFs to clarify this point.

We thank this referee for asking this important question. To answer this question, we have now included data from our *Arabidopsis* line carrying an estradiol inducible PLT3-mVenus construct in wild-type (*Col-0)* background (i. PLT3-mV) to avoid possible disturbances in the SCN from embryogenesis onwards (see Appendix Figure S2).

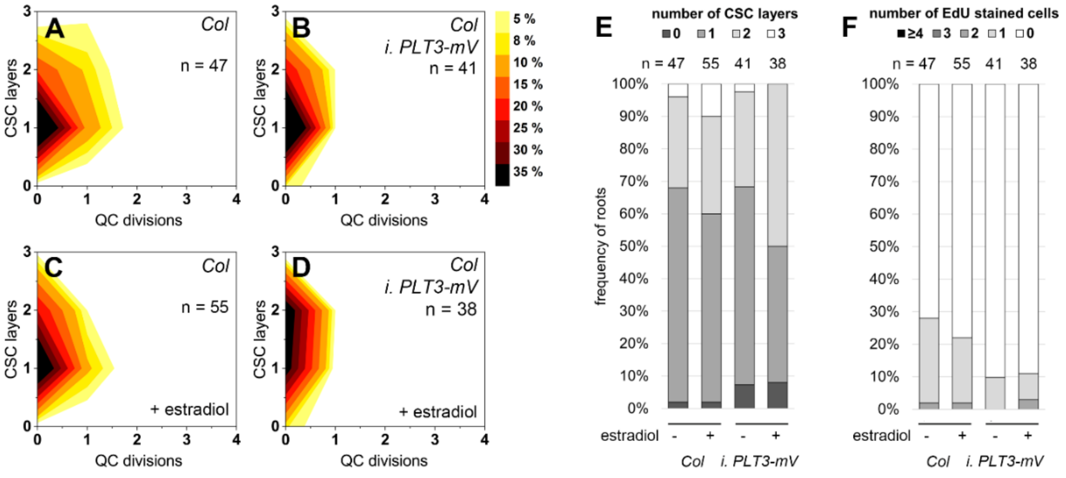

Appendix Figure S2: Ectopic expression of PLT3-mV leads to supernumerary CSC layers

SCN stainings were performed for 24 h on five days old *Arabidopsis thaliana Col-0* seedlings (**A**, **C**) or *Col-0* carrying an estradiol inducible PLT3-mV construct (**B**, **D**). A, B, non-induced seedlings. C, D, seedlings induced with 25 µM β-estradiol. **A**-**D**, combined results of the SCN staining are shown as 2D plots. Number of CSC layers are shown on the y axis and the QC division phenotype is shown on the x axis. The darker the colour, the more roots show the respective phenotype (see colour gradient on the right indicating the frequencies). **E**, **F**, Analyses of the SCN staining for CSC layer (**E**) or QC division (**F**) phenotypes. The frequencies of roots showing 0-3 CSC layers or 0-4 dividing QC cells are plotted as bar graphs. Number of analysed roots (n) is indicated for each genotype and results from three independent experiments. EdU = 5-ethynyl-2´-deoxyuridine; i = inducible; CSC = columella stem cell; QC = quiescent centre.

Here, we found upon induction of PLT3-mVenus an increase in the frequency of two CSC layers from 29 % to 50 %. Here, the effect of enhanced divisions appears to be limited to the CSC, as no additional QC divisions were observed after induction of PLT3-mV (see Appendix Figure S2). Therefore, we argue that PLT3 induction is causal for the observed CSC proliferation. In *plt3* mutants we have observed the opposite effect of less CSC layers and more QC divisions (see Figures 3 and 4). Therefore we reason, that the observed CSC division phenotypes are due to PLT3 function and are not due to potential early embryonic defects that have been reported previously for multiple *plt* mutants (e.g. *plt1*, *plt2* double mutants) (Aida *et al*, 2004) or in *plt3, tcp20, scr* triple or *plt1, plt3, tcp20, scr* quadruple mutants (Shimotohno *et al*, 2018). We have included these results in the Appendix (Appendix Figure S2) and have also added a statement regarding this data to the main text:

Lines 172-176: "*After overexpression of PLT3-mV by estradiol induction in wild-type Col-0 background, we observed the opposite effect, an increase from 29 to 50 % of two CSC layers (see Appendix Figure S2). Therefore, we argue, that the observed CSC phenotypes are due to PLT3 function and are not caused by potential early embryonic defects described previously for multiple plt mutants (Aida et al, 2004).*"

The effect of inducible gain-of-function versions of WOX5 have already been tested by several other independent groups. Here, the analyses of 35S:WOX5-GR lines after induction revealed, that WOX5 overexpression in a wild-type (*Col-0*) background with up to the induction normal SCN organization also leads to ectopic divisions distal to the QC and therefore the accumulation of supernumerary CSC layers (Savina *et al*, 2020; Pi *et al*, 2015; Berckmans *et al*, 2020; Sarkar *et al*, 2007). Thereby it could be demonstrated, that in wild-type roots, ectopic *WOX5* expression is causal for the observed over-proliferation phenotypes. This agrees with our mutant analyses in which we could show for both *plt3* and *wox5* mutants alone and in the higher order multiple mutants a reduction of CSC layers and therefore argue that WOX5 and PLT3 together are responsible for CSC maintenance (see Figures 3 and 4). Therefore, we have also added the following statement to the main text:

Lines 165-167: "*WOX5 was shown to be necessary for CSC maintenance as loss of WOX5 causes their differentiation, while inducible overexpression of WOX5 leads to enhanced proliferation (Savina et al, 2020; Berckmans et al, 2020; Pi et al, 2015; Sarkar et al, 2007).*"

Q2. PLT3 and PLT2 are expressed both in the QC and CSC. It is not clear to this reviewer whether the described phenotypes of plt3,plt2 derive from the activity of PLTs in the QC or in the SCN or both. As authors focus on the different functional interaction of WOX5 and PLTs in the QC and in the CSC, I would suggest authors to provide tissue specific evidence of this different function (e.g. QC/CSC expression of PLT3 in plt3 and wox5 mutants).

We highly appreciate the suggestion of this referee and we have deeply thought about how to carry out the proposed experiments. Finally, we concluded that at this moment these kinds of experiments would be in our opinion extremely difficult to conduct with the needed accuracy and would therefore be very hard to interpret. We would like to explain our conclusion:

Both WOX5 and PLTs were previously described to be mobile TFs (Scheres & Krizek, 2018; Mähönen *et al*, 2014; Burkart *et al*, 2019; Pi *et al*, 2015) and we therefore would expect it to be difficult to interpret data from *Arabidopsis* lines expressing these TFs only from the QC or CSC, as the movement of these TFs within these adjacent domains (from the QC to the CSC or vice versa) would in our opinion very likely prevent a tissue specific functional analysis.

Therefore, and because of the amount of time it would take to produce and test these lines in *Arabidopsis*, we regret that we couldn´t carry out these experiments within the timeframe for the revision of this manuscript and hope the reviewer understands our concerns regarding the difficulty of such experiments and their interpretation at this time.

Q3. I do not understand why authors focus mostly on PLT3 and not other PLTs that are expressed in the QC in a similar fashion (PLT2, PLT1 etch...), despite to lower (?) extent. This point should be better explained.

We thank this referee for the chance to clarify this important point and agree, that in the manuscript we did not sufficiently explain our choice. We are aware of the redundancy of PLTs in the root SCN and that multiple *plt* mutants show very severe root phenotypes (Aida *et al*, 2004; Galinha *et al*, 2007).We focused our research on PLT3, as it is the PLT that is expressed highest in the CSCs in comparison to the other PLTs expressed in the root SCN (Aida *et al*, 2004) and, importantly, because we found it to produce more NBs than all the other PLTs (which we have also quantified now, see response to Q4 below).

Furthermore, PLT3, also known as AIL6, was recently predicted as one of the central nodes regulating other QC-enriched TF in the underlying gene regulatory network within the *Arabidopsis* root SCN. In contrast, PLT1 and PLT2 were predicted as minor nodes only and PLT4 (BBM) was not predicted as a node (de Luis Balaguer *et al*, 2017). We have added an explanatory sentence to the main text and hope that it is now more comprehensible for the reader why we focus mostly on PLT3.

We have therefore added this explanatory section to the main text:

Lines 95-100: "*...where, in comparison to all the other PLTs, particularly PLT3 is highly expressed (Figure 1B) (Galinha et al, 2007). Furthermore, PLT3 was recently predicted as one of the central nodes regulating other QC-enriched TF in the underlying gene regulatory network (GRN) within the Arabidopsis root SCN. In contrast, PLT1 and PLT2 were predicted as minor nodes only and PLT4 (BBM) was not predicted as a node (de Luis Balaguer et al, 2017).*"

We additionally chose PLT2 for single and multiple mutant analyses as a second representative of the four PLTs expressed in the SCN because it has a similar arrangement of PrDs, which we found most important in our localization and interaction assays, within its protein sequence. We avoided to use higher order PLT mutants and especially the *plt1* mutants as they show very severe (and probably pleiotropic) root phenotypes which would make interpretation of the rather subtle QC and CSC phenotypes difficult. We have added part of this explanation to the main text and hope that this reviewer and the readers will find it explanatory and easy to follow:

Lines 118-121: "*PLTs are known for their redundant function in SCN maintenance, that can be very strong especially when PLT1 is mutated in combination with other PLTs (Aida et al, 2004; Galinha et al, 2007). Because we aimed to look at the rather subtle QC and distal SCN phenotypes, we therefore included only plt2 mutants for our analyses.*"

Q4. L258-261- The authors nicely show that having PrD PLT3 forms nuclear bodies, and they show that other PLTs have the PrD domain but lack or show short Q rich domain. It is not clear to this reviewer whether the ability to form nuclear bodies is a characteristic only of PLT3 or also of the other PLTs. Authors should clarify this point.

We fully agree with this reviewer and reviewer #2 that we need to clarify this point. Therefore, we have now acquired quantitative data on the number of NBs that form after exactly 5 hours of estradiol induction in PLT1, PLT2, PLT3 and PLT4 expressing *N. benthamiana* (see below). All imaging acquisition settings were kept the same to assure comparability between the samples. We have included this data in Appendix Figure S3 and Appendix Table S11.

In these experiments we found, that PLT3 shows the most NBs (at least three times more) in comparison to PLT1, PLT2 and PLT4. PLT1, PLT2 and PLT4 also form NBs at a similar level.

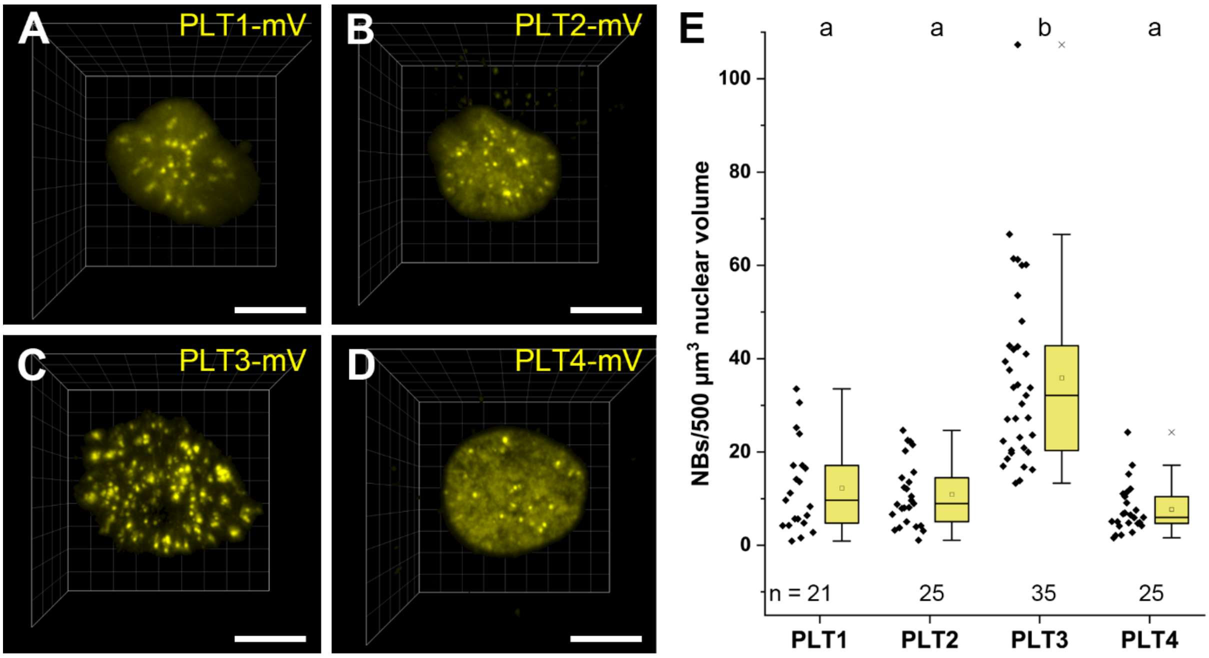

Appendix Figure S3: Quantification of NBs in PLT1-4

A-D, Representative 3D images of nuclei expressing PLT1-mV (**A**), PLT2-mV (**B**), PLT3-mV (**C**), and PLT4-mV (**D**) in *N. benthamiana* leaf epidermis 5 hours after induction with β-estradiol. Scalebars represent 10 µm. **E**, quantification of the number of NBs per 500 μm^3 nuclear volume. Box = 25-75 % of percentile, whisker = 1.5 interquartile range, – = median, □ = mean value, × = outliers. The Kruskal-Wallis ANOVA analysis with subsequent Dunns test was used to test for statistical significance. Samples with identical letters do not show significant differences ($\alpha = 0.01$). The number of nuclei (n) is indicated for each sample and results from two independent experiments. NB = nuclear bodies; mV = mVenus.

We have added the following statement to the main text for clarification:

Lines 243-247: "*We quantified NB formation of PLTs in this transient system using estradiol inducible versions of PLT1-4 tagged with mVenus by acquiring z-stacks through the expressing nuclei exactly 5 hours after induction. Here, we found that PLT3 forms at least three times more NBs compared to PLT1, PLT2 and PLT4 (see Appendix Figure S3 and Appendix Table S11).*"

Minor points:

Q5. L84-authors should report some examples of the several physiological reactions of NBs to enable readers to critically understand their findings.

We agree with the referee and have added more examples of some of the until now suggested physiological roles of NBs to the main text (see below):

Lines 84-86: "*…self-assembling protein/RNA containing compartments thought to regulate a variety of physiological responses to differential environmental cues like light, temperature or osmotic changes (Meyer, 2020; Mao et al, 2011; Jung et al, 2020).*"

Lines 443-445: "*Another recently described PrD- and polyQ-containing protein, EARLY FLOWERING3 (ELF3), forms NBs by LLPS in response to temperature thereby regulating flowering time in Arabidopsis (Jung et al, 2020).*"

Q6. Fig.2 C is out of focus and should be replaces.

We agree with the referee's opinion and have replaced the image in Figure 2C.

Referee #2:

The manuscript by Stahl and co-workers re-investigates the PLT-WOX regulation with respect to mutual transcription regulation and confirms earlier findings that PLT confines WOX5 expression whereas WOX5 promotes PLT expression. This feedback loop is important for QC function and CSC maintenance. However, in terms of QC quiescence these genes appear to be working in parallel. These different mechanisms of action indicate the complex relation of PLT and WOX5 genes in the root stem cell niche. The authors show detailed analyses of the phenotypes in single and multiple mutants for support and correlate the CSC differentiation with increased QC divisions as a mechanism to replenish lost stem cells. The manuscript becomes interesting when the authors look carefully at the localization of the proteins inside the nucleus. Depending on the position of the cell in the developing organ (CSC or LRP) the PLT proteins localize in nuclear bodies and sequester also WOX5. This can explain the "acting together to maintain CSC homeostasis" that was derived from the phenotypic mutant analyses looking at CSC differentiation. The NB localization is coupled to the presence of so-called prion domains in the PLT proteins which leads the authors to propose a function for this localization as being a determinant factor for stem cell and future CC fate.

The experiments are performed with high quality incorporating detailed phenotyping, novel staining methods and high-tech interaction studies.

Below I will remark on the results described in this manuscript.

Q1. Ln89 and throughout the manuscript there is a mixed use of "CSC" and "distal stem cell". These are the same cell and maybe I missed it but it would be good to clearly state this somewhere in the beginning.

We thank the referee for making us aware of this mixed use of “CSC” and “distal stem cells” and we have now clarified that they are the same cell types in the introduction.

Line 63: “…*CSCs, also called distal stem cells,…*”

Q2. Ln143-153 and Fig 3. This section describes the CSC differentiation using the new staining method. First, it will help the reader if the images contain marks, e.g. arrowheads, that point out the QC or mutant QC and the CSC or mutant CSC. For example, it is not obvious where the QC is in fig 3H.

We fully agree with the referee and have now marked the QC positions in the longitudinal and transversal optical sections throughout Figure 3 and hope that the images are now more accessible to the reader.

Q3. Second, dividing cells stained through EDU accumulation containing little starch are scored as differentiated cells thereby disturbing the starch free CSC layer which is therefore scored as a differentiating CSC layer. This is of course a way of scoring but it also means that there are stem cells remaining even when some CSCs in the layer are differentiating. Some nuancing helps to make this clear.

We thank the reviewer for the chance to better explain this important aspect of the SCN staining analysis. CSC layer and QC division scoring is performed separately on images from the same root. CSC layer scoring is only done on the longitudinal optical section. We defined an intact CSC layer as a stem-cell layer below QC position without any differentiated cells (visible by no starch-accumulation).

Cells in the layer below QC position containing starch granules are scored as differentiated, and we therefore score the whole layer as differentiated, even if only some of these cells contain starch. This is because usually CSCs divide in a coordinated manner, all at the same time (visible as 2 CSC layers in around 25% of *Col-0* roots), and only after this differentiate and accumulate starch.

We have added this explanation to the materials and methods section for clarification of our scoring routine:

Lines 593-597: "*For this, we defined an intact CSC layer as a stem-cell layer below QC position without any differentiated cells (visible by no starch-accumulation). Cells in the layer below QC position containing starch granules were scored as differentiated, and we therefore score the whole layer as differentiated, even if only some of these cells contain starch.*"

Q4. Even so, is there evidence that these EDU stained but starch containing CSCs cells are truly differentiating when they appear to be/have divided recently. Or is it a matter of definition. It will be good to make this clear.

We thank the referee for this important question. Cells below the QC region which contain starch are by our definition differentiated columella cells (CCs). CCs can also divide at some point and therefore EdU can be incorporated during the S-phase of their cell cycle. This can also be observed in CCs further distal to the QC.

Q5. Ln157, Fig1 should be Fig3

We apologize for the mislabelling and have changed Figure 1 to Figure 3.

Q6. Ln158, I would change the "QC within 24 hours" into "QC within the 24 hours staining window"

We agree with the referee´s suggestion and have changed the main text according to the referees' suggestion in line 186.

Q7. Ln160, there is QC division frequency in the plt2plt3 double that is 57%. However, this double shows a duplicated QC according to Fig2D. Therefor I wonder if only a single plane is used for counting the divisions and whether this plane represents the bottom layer of QC cells. The authors should make this clear.

We thank the reviewer for giving us the chance to further clarify the method of SCN analyses in the case of duplicated QC cells as in *plt2, plt3* double mutants. Here, only one layer of QC cells, the one with the most QC divisions, was counted. This is indeed usually the bottom layer. We have added an explanatory sentence to the materials and methods section for clarification.

Lines 600-601: "*In case of duplicated QC cells as in plt2, plt3 double mutants only one layer of QC cells, the one with the most QC divisions, was counted (usually the bottom layer).*"

Q8. Ln170, states "according to our hypothesis". I may have missed it but I did not find this hypothesis stated before, only that in line 124 the authors intend "to probe for their interdependency". Please rephrase.

We thank the referee for this observation and changed this sentence for clarity.

Lines 197-199: "*Taken together, this data implies an additive effect of PLT2, PLT3 and WOX5 regarding the QC-division phenotype, suggesting that WOX5 and PLTs act in parallel pathways to maintain the quiescence of the QC.*"

Q9. Ln215, 245, 247, 261 and maybe at more places, Suppl fig 3 should be 4

We apologize for this mislabelling and have changed the main text accordingly (Suppl Fig 3 is now called Figure EV4 throughout).

Q10. Regarding Suppl Fig3, there are no arrowheads as indicated in the legend

We thank the reviewer for noticing the missing arrowheads. We have now added them to this Figure (now called Figure EV3).

Q11. Ln257-61 states that the PLT PrD domains are responsible for the recruitment of WOX5 to NBs. I have a few points that I like to raise here. First, I wonder if WOX5 is also recruited to the NB in LRPs. WOX5 is expressed already in stage II primordia and therefore it is possible to check for colocalization. Please test this.

We thank the reviewer for the chance to explain this. We did check for this in the *Arabidopsis* line that we used for FLIM, but, unfortunately, the expression of WOX5 driven by its endogenous promoter is too weak in the early LRP to detect if it is recruited to NBs.

Q12. The advantage being that here the NB are seen often and pronounced according to the authors. It will provide additional relevance for the proposed interaction. Also, as mentioned in the next pages, if the co-localisation is observed it provides the authors with a better opportunity to test interaction with FLIM in vivo.

As mentioned above, unfortunately, the expression of WOX5 is too weak, especially in the CSCs, to observe NBs. Nevertheless, we were able to measure FRET-FLIM in *Arabidopsis* LRP and observed a small but significant lifetime reduction as a sign of *in vivo* protein-protein interaction of WOX5 and PLT3 (as shown in Figure EV3).

Q13. Second, I am confused about the "most frequently" since the authors show that all PLTs are observed in NB in Suppl Fig4. How often do the authors actually see localization in NB in betha leaf cells for the different PLTs?

We fully agree with this reviewer and reviewer #1 that we should clarify this point. Therefore, we have now acquired quantitative data on the number of NBs that form after exactly 5 hours of estradiol induction in PLT1-4 expressing *N. benthamiana* (see below and as Appendix Figure S3). All imaging acquisition settings were kept the same to assure comparability between the samples. We have included this data in Appendix Figure S3 and Appendix Table S11.

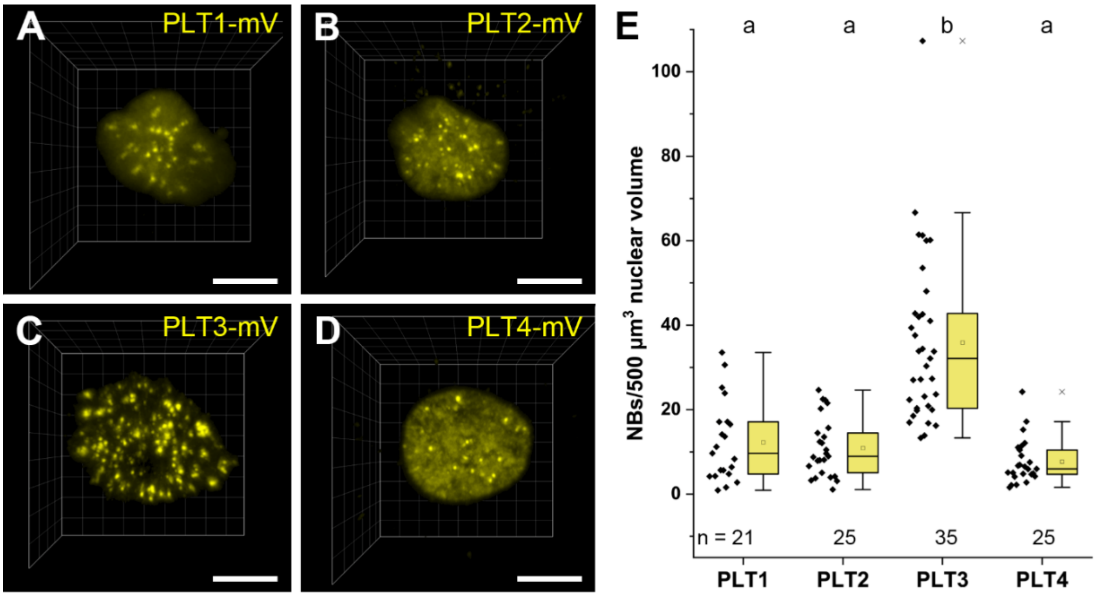

Appendix Figure S3: Quantification of NBs in PLT1-4

A-D, Representative 3D images of nuclei expressing PLT1-mV (**A**), PLT2-mV (**B**), PLT3-mV (**C**), and PLT4-mV (**D**) in *N. benthamiana* leaf epidermis 5 hours after induction with β-estradiol. Scalebars represent 10 µm. **E**, quantification of the number of NBs per 500 μm^3 nuclear volume. Box = 25-75 % of percentile, whisker = 1.5 interquartile range, – = median, □ = mean value, × = outliers. The Kruskal-Wallis ANOVA analysis with subsequent Dunns test was used to test for statistical significance. Samples with identical letters do not show significant differences (α = 0.01). The number of nuclei (n) is indicated for each sample and results from two independent experiments. NB = nuclear bodies; mV = mVenus.

In these experiments we found, that PLT3 shows the most NBs (at least three times more) in comparison to PLT1, 2 and 4. PLT1, 2 and 4 also form NBs at a similar level.

Therefore, we have added the following statement to the main text for clarification:

Lines 243-247: "*We quantified NB formation of PLTs in this transient system using estradiol inducible versions of PLT1-4 tagged with mVenus by acquiring z-stacks through the expressing nuclei exactly 5 hours after induction. Here, we found that PLT3 forms at least three times more NBs compared to PLT1, PLT2 and PLT4 (see Appendix Figure S3 and Appendix Table S11).*"

Q14. Ln283, Fig 7H should be I

Ln292, Fig 7I,J should be J,K

We thank the referee for making us aware of our mislabelling and changed the main text accordingly.

Q15. Ln293-301. Did the authors measure the interaction of WOX5 and PLT3 using the complemented mutants? Please state.

The FRET-FLIM experiments showing interaction of WOX5 with PLT3 in *Arabidopsis* were done in *Col-0* wild-type roots expressing pPLT3:PLT3-mCherry and pWOX5:WOX5-mVenus. We have now indicated this in the figure legend (Figure EV3) and main text (line 339).

Q16. And, earlier the authors show there are NBs observed in the root niche as shown in Fig5. Then why not cross lines in which the NBs are observed. And coming back to my earlier remark, in Fig5A there is clear NB in multiple cells of the LRP. Please try to measure interaction in these cells if WOX5 is colocalized to NBs here.

Unfortunately, as already stated above, the expression of WOX5, also in the early LRPs, is too weak to measure FRET-FLIM, even though we are using very sensitive τ-SPAD avalanche photodiode detectors. Hopefully in the future, when even more sensitive detectors become available, it will be possible to measure this.

Q17. And Suppl Fig4 should be 3 now.

We thank the referee for making us aware of this mislabelling and have changed the main text accordingly.

Q18. Ln320-1 states the PrD containing the polyQ are required for NB formation and WOX5 complex formation. Still, the NB form in the PLT3 polyQ mutant but no PLT3-WOX5 interaction takes place indicating that the polyQ is required for interaction. However, also interaction of WOX5 to PLT1 and 2 is observed which do not have polyQ annotated. Possibly the polyQ deletion disturbs the PrD domain which is the reason that interaction does not take place? Possibly polyQ annotation is not straightforward. I feel that now there is emphasis on polyQ required for complex formation, e.g. ln379, whereas this may not be so straighforward. Please discuss.

We thank the reviewer for raising this important point. We agree with the referee, that based on our experiments we cannot rule out that the deletion of the polyQ domains could disturb the whole PrD domain, and that this might be the reason for the disturbed protein-protein interaction of WOX5 and PLT3, while NB formation is still possible. Also, the significantly smaller polyQ streches in PLT1 and 2 (which we did not classify as polyQ domain, as we only counted more than three Qs in a row as polyQ; we added this information to the legend of Figure EV4) and the smaller polyQ domain in PLT4 could still serve as interaction sites for WOX5. In the future more deletion variants of different PLTs and analyses of PPI of these with WOX5 would need to be done to clarify this. Here, not only a general lifetime reduction, but novel advances to calculate the binding affinities by analysing the FRET FLIM data would certainly help to illuminate the involvement of the different domains

necessary to interact with WOX5. We have discussed the raised issues now in the main text and toned down our hypothesis regarding the importance of polyQ domains for PPI with WOX5:

Lines 363-368: "*Still, the exact effect of the polyQ domains on PLT-WOX5 interaction remains to be determined as PLT1, PLT2 and PLT4 still show protein-protein interaction with WOX5, even though they do not contain big polyQ domains like in PLT3. We therefore cannot rule out that the deletion of the polyQ domain in PLT3 (PLT3ΔQ) could leads to a disturbed PrD domain resulting in a loss of interaction with WOX5.*"

Line 432: *"…PrDs (including polyQs)…"*

Line 464: "*poly Q domains*" deleted

Line 489: *"…intrinsically disordered domains, such as polyQ-stretches which…"*

Q19. Ln331-3. I feel this single summarizing sentence does not give credit to the complex relation between PLTs and WOX5 and the results that the authors add to this relation here. Please rephrase in few sentences.

We agree with the referee, and we have rephrased this part now to better reflect the highly intertwined regulations on transcriptional and PPI level, as well as on the observed compartmentalization of PLT3 and WOX5 to NBs.

Lines 378-384: "*In summary, our findings show that QC quiescence and CSC maintenance are interdependently regulated by WOX5 and PLTs. We could show that mutual transcriptional regulation of PLTs and WOX5, but also their direct protein-protein interaction is required for QC division and CSC fate regulation. Here, especially the observed subnuclear partitioning of PLT3 to NBs in the CSCs of mature RAMs which is dependent on the presence of PrDs is important as it could provide another layer of regulation to the complex and intertwined SCN maintenance of the Arabidopsis root.*"

Referee #3:

The manuscript by Burkart et al., presents interesting new findings about the role of well known plants specific transcription factors, PLTs and WOX5. Over the years both the factors have been studied in the context of root development and their role in regulating the maintenance of the stem cell niche is well established. So far, people in the field have been looking at these regulators like many other accessory transcription factors and thought of their predominant role as nuclear localized proteins. This study provokes new thinking and convincingly shows that there is a clear sub-nuclear localization in the columella stem cell niche. They further went ahead and showed that PLT3 interacts with WOX5 and gets recruited to nuclear bodies. This recruitment is important for maintenance of the columella stem cell niche, well supported by genetics. In general I like this part of the work. These transcription factors also regulate various regenerative responses in plants, the present work opens up the possibilities of investigating the importance of such subnuclear localization in other contexts of the development too.

I have the following points:

Q1. Regarding the transcriptional regulation of PLT3 by WOX5, is it direct or indirect? The authors should check whether PLT3 can be rapidly upregulated by induction of WOX5. They can use steroid inducible versions of WOX5-GR to address this. Or do they imply that transcription regulation of PLT3 by WOX5 is an indirect one? This is important as PLT1 and PLT3 were shown to directly regulate WOX5 transcription (Shimotohno et al, 2018).

We thank the referee for this important question and conducted the suggested experiments. We have carried out two independent experiments utilizing two published *Arabidopsis* lines (35S:WOX5-GR, (Sarkar *et al*, 2007) and 35S::WOX5-GFP-GR (Berckmans *et al*, 2020) to test for direct or indirect upregulation of *PLT3* expression after WOX5 induction (see Appendix Figure S1, Appendix Tables S12 and S13).

In our quantitative PCR (qPCR) experiments (Appendix Figure S1A) we could not detect a significant change of *PLT3* expression after induction of WOX5 for 4 hours with DEX. We could only observe a CHX-induced upregulation of *PLT3* expression that was not significantly different if the seedlings were simultaneously induced with DEX or not. This generic upregulation upon CHX treatment has been observed before (Shimotohno *et al*, 2018). We confirmed these results by analysing F1 seedlings of crosses of 35S::WOX5-GFP-GR with pPLT3::erCFP lines (Appendix Figure S1B-E). Also in these experiments, we did not detect any significant changes of *PLT3* expression after 4 h of DEX induction in any of the three different regions of interest (ROI) analysed. Only after a prolonged DEX-induction for 21 h we detected a significant up to two-fold increase of *PLT3* expression. This is in agreement with published qPCR results using roots of 5 days old 35S::WOX5-GR seedlings, showing a two-fold increase of *PLT1* expression only after 24 h of DEX treatment, but not after 2 h of induction (Ding & Friml, 2010).

Therefore, we conclude that *PLT3,* like *PLT1,* is not directly transcriptionally regulated by WOX5, but only indirectly, possibly by other factors, such as the described WOX5-induced TAA1-mediated auxin biosynthesis (Savina *et al*, 2020; Pardal & Heidstra, 2021). On the other hand, as this referee mentioned, a direct regulation of *WOX5* expression by PLT1 and PLT3 has been demonstrated by similar experiments before (Shimotohno *et al*, 2018) reflecting the complex and intertwined regulations of these factors in the RAM.

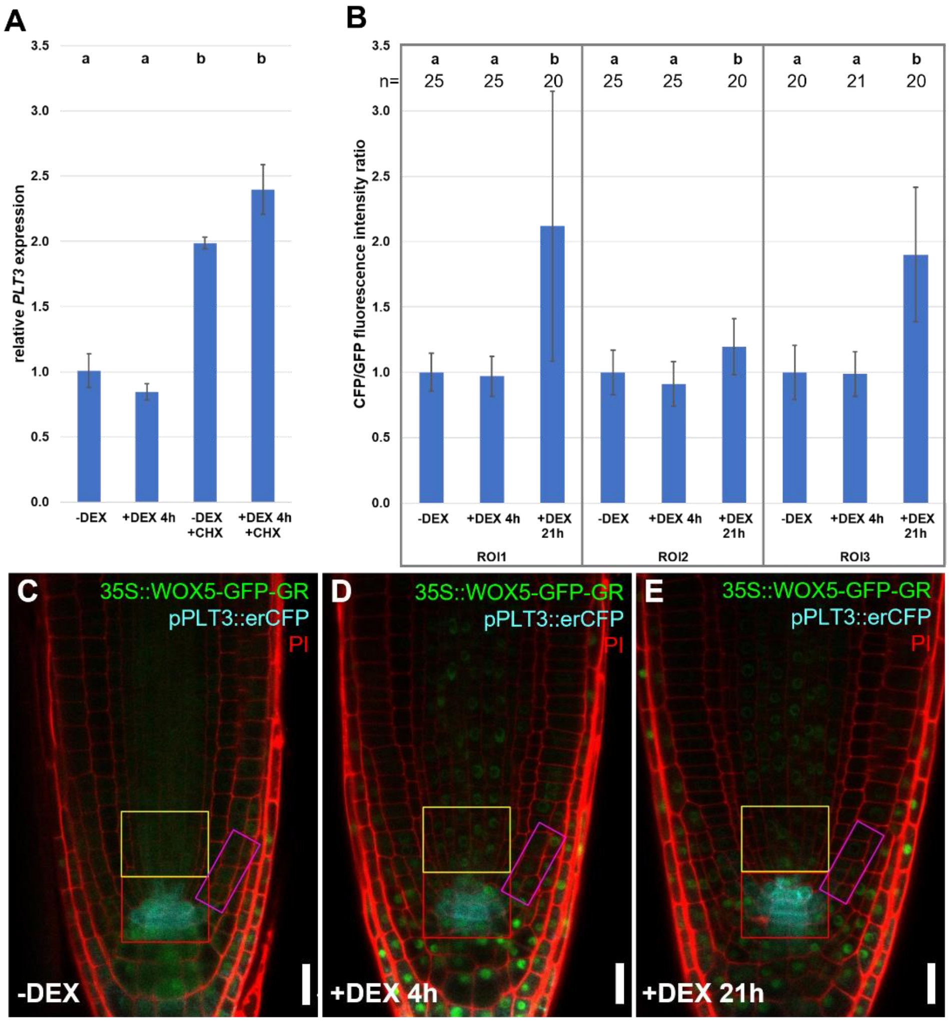

Appendix Figure S1: Quantification of PLT3 expression after WOX5 induction

A, qPCR analyses on root RNA isolated from 5 days old 35S:WOX5-GR *Arabidopsis* seedlings uninduced or induced for 4 h with 20 µM dexamethasone (DEX) with or without simultaneous incubation with 10 µM cycloheximide (CHX). Three biological replicates and two technical replicates per treatment were carried out. The data was normalized to the -DEX sample. Error bars show standard deviations. **B**, Quantification of CFP/GFP fluorescence intensity ratios in 5 days old *Arabidopsis* roots of F1 crosses (35S::WOX5-GFP-GR × pPLT3::erCFP) without induction (-DEX), or induction with dexamethasone (+DEX) for 4 or 21h. The data was normalized to the not induced roots (-DEX) per analysed region of interest (ROI). Error bars represent standard deviations. The data was statistically analysed by one-way ANOVA and Holm-Sidak post-hoc multiple comparisons test. Samples with identical letters do not show significant differences ($\alpha = 0.01$). The number of roots (n) is indicated for each sample and results from two independent experiments. **C-E**, representative images of the roots analysed in **B**. Cell walls are stained by propidium iodide (PI) in red. ROI1 is marked in red, ROI2 is marked in yellow, ROI3 is marked in magenta. Scalebars represent 10 µm.

Therefore, we have added the following explanations to the main text and additionally adjusted Figure 8A (see in response to Q3):

Line 104-111: *"Next, we addressed, if PLT3 expression is regulated directly or indirectly upon WOX5 induction by using the published Arabidopsis lines 35::WOX5-GR (Sarkar et al, 2007) and 35S::WOX5-GFP-GR (Berckmans et al, 2020) in quantitative PCR experiments (qPCR) and crosses with pPLT3::erCFP (Galinha et al, 2007), respectively. In both independent experiments we found no change of PLT3 expression after 4 h of WOX5 induction. After 21 h of WOX5 induction we found PLT3 expression significantly upregulated up to two-fold and therefore, we conclude that PLT3 expression is not directly regulated by WOX5 (Appendix Figure S1, Appendix Table S12 and S13)."*

Line 113: *"…albeit in an indirect manner."*

Line 387-389: *"…, albeit in an indirect manner, possibly by other factors, such as the described WOX5-induced TAA1-mediated auxin biosynthesis (Pardal & Heidstra, 2021; Savina et al, 2020). This PLT expression confines…"*

Q2. What made the authors analyse plt2,plt3 double mutant? I can understand that based on predominant expression of PLT3 in distal stem cells they chose plt3, but why plt2 ? Why not plt1 mutant? In the result section it reads as an abrupt choice given the redundant role of PLT1, PLT2 and PLT3 in maintenance of the root stem cell niche.

We thank the reviewer for the chance to explain this choice better. We chose PLT3 because it has a predominant expression in the distal stem cells (Galinha *et al*, 2007). Recently, PLT3 was also identified as a central node in the regulation of TFs involved in root SCN maintenance, whereas PLT1 and PLT2 were identified as minor nodes (de Luis Balaguer *et al*, 2017). And, importantly, PLT3 shows the most NBs in comparison to PLT1, 2 and 4 (see new quantification in Appendix figure S3 and Appendix Table S11). We have added the following statement to the main text:

Lines 95-100: "*…where, in comparison to all the other PLTs, particularly PLT3 is highly expressed (Figure 1B) (Galinha et al, 2007). Furthermore, PLT3 was recently predicted as one of the central nodes regulating other QC-enriched TF in the underlying gene regulatory network (GRN) within the Arabidopsis root SCN. In contrast, PLT1 and PLT2 were predicted as minor nodes only and PLT4 (BBM) was not predicted as a node (de Luis Balaguer et al, 2017).*"

We are aware of the redundancy of the PLTs and we chose to use the *plt2* mutant for double mutant analyses because we did not want to use too strong mutant alleles (like the *plt1* mutant) in combination with *plt3* as we intended to avoid too strong and possibly pleiotropic effects which were previously described for *plt1* multiple mutants, e.g. extremely disturbed SCN, very short root phenotypes or even failure of root formation (Aida *et al*, 2004; Galinha *et al*, 2007). Furthermore, PLT2 also shows a similar arrangement of a C-terminal and a N-terminal PrD domain like PLT3, but far less glutamines. We have added explanatory sentences regarding this choice to the main text:

Lines 118-121: "*PLTs are known for their redundant function in SCN maintenance, that can be very strong especially when PLT1 is mutated in combination with other PLTs (Aida et al, 2004; Galinha et al, 2007). Because we aimed to look at the rather subtle QC and distal SCN phenotypes, we therefore included only plt2 mutants for our analyses.*"

Q3. Also, while interpreting the significance of regulation of WOX5 by PLTs and vice versa, they should take into account the fact that WOX5 is under direct transcriptional regulation of PLT. Now that they have a clearer picture of additional PLT-WOX5 interaction (both at the level of protein-protein interaction and protein-DNA interaction) they must provide a model for much deeper insights into the cumulative role of PLTs and WOX5.

We agree with the referee that we did not discuss this regulation extensively in the main text. As already mentioned above in response to Q1 of this referee, we have carried out the suggested experiments and have added the following paragraph to the main text:

Line 104-111: *"Next, we addressed, if PLT3 expression is regulated directly or indirectly upon WOX5 induction by using the published Arabidopsis lines 35::WOX5-GR (Sarkar et al, 2007) and 35S::WOX5-GFP-GR (Berckmans et al, 2020) in quantitative PCR experiments (qPCR) and crosses with pPLT3::erCFP (Galinha et al, 2007), respectively. In both independent experiments we found no change of PLT3 expression after 4 h of WOX5 induction. After 21 h of WOX5 induction we found PLT3 expression significantly upregulated up to two-fold and therefore, we conclude that PLT3 expression is not directly regulated by WOX5 (Appendix Figure S1, Appendix Table S12 and S13)."*

Line 113: *"…albeit in an indirect manner."*

Line 387-389: *"…, albeit in an indirect manner, possibly by other factors, such as the described WOX5-induced TAA1-mediated auxin biosynthesis (Pardal & Heidstra, 2021; Savina et al, 2020). This PLT expression confines…"*

Line 468: *"…or repress WOX5 expression."*

We have also adjusted the models in Figure 8 accordingly (see below):

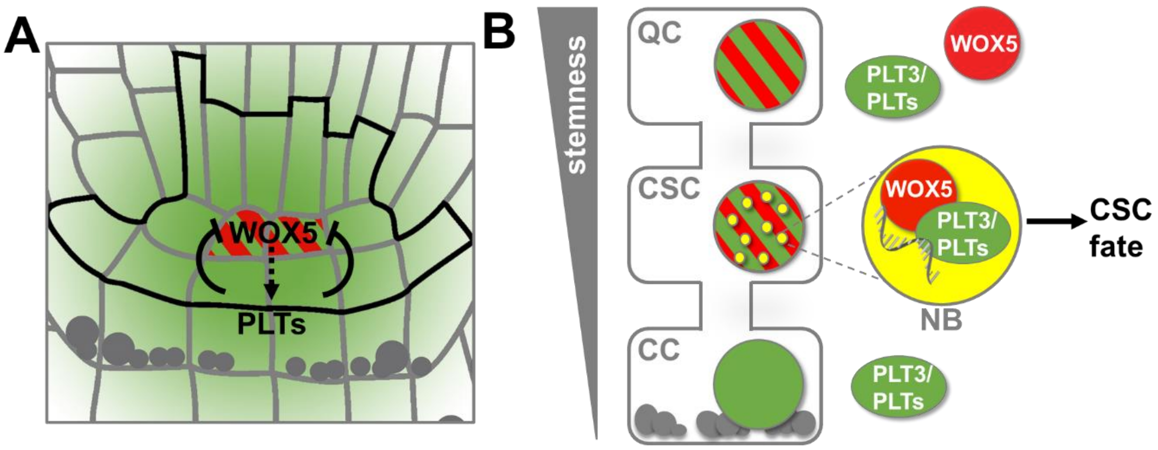

Figure 8 - Model of PLT and WOX5 transcriptional regulation, interaction and subnuclear localisation during distal root stem cell maintenance.

A, Transcriptional regulation of *WOX5* (red) and *PLT* (green) expression by negative feedback regulation in the *Arabidopsis* RAM. *WOX5* is expressed in the QC and indirectly promotes *PLT* expression (dashed arrow), whereas *PLT* expression is directly regulating *WOX5* expression confining it to the QC position (solid barred line). **B**, Both WOX5 (red) and PLT3 (green) are present homogenously within the nuclei of the QC cells. WOX5 can move to the CSCs and is recruited there by PLT3 to NBs (yellow), where interaction takes place and RNA is present (grey in magnification). This maintains the stem cell character of the CSCs (arrow) but already leads to a determination to subsequent CC fate. Gray dots represent starch granules.

Q4. Throughout the figures, I did not find "n" (total number of samples/root tips) taken for various reporter and mutant analysis. Please indicate that on the panels wherever it is applicable.

We thank the reviewer for making us aware of this. We have now added the total number of samples and number of independent experiments throughout all figure panels and figure legends where applicable and have also increased the number of analyzed roots used in Figure 1 and 2 (see also updated Appendix Table S5 and S6).

References

Aida M, Beis D, Heidstra R, Willemsen V, Blilou I, Galinha C, Nussaume L, Noh Y-S, Amasino R, Scheres B (2004) The PLETHORA genes mediate patterning of the Arabidopsis root stem cell niche. *Cell* 119: 109–120

Berckmans B, Kirschner G, Gerlitz N, Stadler R, Simon R (2020) CLE40 Signaling Regulates Root Stem Cell Fate. *Plant Physiol* 182: 1776–1792

Burkart RC, Strotmann VI, Kirschner GK, Akinci A, Czempik L, Maizel A, Weidtkamp-Peters S, Stahl Y (2019) PLETHORA and WOX5 interaction and subnuclear localisation regulates Arabidopsis root stem cell maintenance. *bioRxiv:* DOI: 10.1101/818187

de Luis Balaguer MA, Fisher AP, Clark NM, Fernandez-Espinosa MG, Möller BK, Weijers D, Lohmann JU, Williams C, Lorenzo O, Sozzani R (2017) Predicting gene regulatory networks by combining spatial and temporal gene expression data in Arabidopsis root stem cells. *Proc Natl Acad Sci USA* 114: E7632-E7640

Ding Z, Friml J (2010) Auxin regulates distal stem cell differentiation in Arabidopsis roots. *Proc Natl Acad Sci USA* 107: 12046–12051

Galinha C, Hofhuis H, Luijten M, Willemsen V, Blilou I, Heidstra R, Scheres B (2007) PLETHORA proteins as dose-dependent master regulators of Arabidopsis root development. *Nature* 449: 1053–1057

Mähönen AP, Tusscher K ten, Siligato R, Smetana O, Díaz-Triviño S, Salojärvi J, Wachsman G, Prasad K, Heidstra R, Scheres B (2014) PLETHORA gradient formation mechanism separates auxin responses. *Nature* 515: 125–129

Pardal R, Heidstra R (2021) Root stem cell niche networks: it's complexed! Insights from Arabidopsis. *J Exp Bot* 72: 6727–6738

Pi L, Aichinger E, van der Graaff E, Llavata-Peris CI, Weijers D, Hennig L, Groot E, Laux T (2015) Organizer-Derived WOX5 Signal Maintains Root Columella Stem Cells through Chromatin-Mediated Repression of CDF4 Expression. *Dev Cell* 33: 576–588

Sarkar AK, Luijten M, Miyashima S, Lenhard M, Hashimoto T, Nakajima K, Scheres B, Heidstra R, Laux T (2007) Conserved factors regulate signalling in Arabidopsis thaliana shoot and root stem cell organizers. *Nature* 446: 811–814

Savina MS, Pasternak T, Omelyanchuk NA, Novikova DD, Palme K, Mironova VV, Lavrekha VV (2020) Cell Dynamics in WOX5-Overexpressing Root Tips: The Impact of Local Auxin Biosynthesis. *Front Plant Sci* 11: 560169

Scheres B, Krizek BA (2018) Coordination of growth in root and shoot apices by AIL/PLT transcription factors. *COPB* 41: 95–101

Shimotohno A, Heidstra R, Blilou I, Scheres B (2018) Root stem cell niche organizer specification by molecular convergence of PLETHORA and SCARECROW transcription factor modules. *Genes Dev* 32: 1085–1100

1st Revision - Editorial Decision 17th Mar 2022

Dear Prof. Stahl,

Thank you for the submission of your revised manuscript to our editorial offices. I have received the reports from the three referees that were asked to re-evaluate your study, you will find below. As you will see, the referees now fully support publication of your work in EMBO reports.

Before we can proceed with formal acceptance, I have these editorial requests I also ask you to address in a final revised manuscript:

- I could not find the files for the EV figures. Please provide these as production quality figure files as .eps, .tif, .jpg (one file per figure). Please upload these as separate, individual files upon re-submission.

- Please make sure that the number "n" for how many independent experiments were performed, their nature (biological versus technical replicates), the bars and error bars (e.g. SEM, SD) and the test used to calculate p-values is indicated in the respective figure legends (main and EV figures), and that statistical testing has been done where applicable. Please avoid phrases like 'independent experiment', but clearly state if these were biological or technical replicates. Please add complete statistical testing to all diagrams. Please also indicate (e.g. with n.s.) if testing was performed, but the differences are not significant.

- The description of the replicates in the figure legends is not clear. E.g. for Fig. 1D you state 'Number of analyzed roots (n) is indicated for each genotype and results from two independent experiments (biological replicates)'. In 1D there are 24 and 27 datapoints shown, which you state are from different roots (plants?). Thus, it seems we have here 24 and 27 biological replicates. Now, it is unclear what 'results from two independent experiments (biological replicates)' here means. Or, are these 27 (24) technical replicates, and the diagram shows one of two independent experiments? For Fig. 7K it is stated: 'n = 7, from four independent experiments (biological replicates)'. Again, there are (maybe) 7 datapoints for each condition (bodies-only, nucleoplasm). Are these then technical replicates and the diagram is representative of one biological replicate (out of 4)? Similar wording can be found in most of the data information paragraphs (main figures) or the EV figure legends. Please go through all of these and clarify.

- We updated our journal's competing interests policy in January 2022 and request authors to consider both actual and perceived competing interests. Please review the policy https://www.embopress.org/competing-interests and add a statement declaring your competing interests. Please name that section 'Disclosure and Competing Interests Statement' and add it after the acknowledgements section.

- There seems to be no callout for panel 7F. Please check.

- We need separate (and sequential) callouts for all the figure panels, including EV and Appendix figures. There are no separate callouts for the panels of Fig. 8 and the panels of the three Appendix figures. Please add these. As the EV figures have not been uploaded, it is presently unclear if their panels haven been called out separately. Please check and in case add separate callouts

- Please name the "Methods" section "Materials and Methods".

- Please provide the legend for Movie EV2 as text file and upload this ZIPed together with the movie file. Finally, please remove the movie legend from the main manuscript text.

In addition, I would need from you:
- a short, two-sentence summary of the manuscript (not more than 35 words).
- two to four short bullet points highlighting the key findings of your study
- a schematic summary figure (in jpeg or tiff format with the exact width of 550 pixels and a height of not more than 400 pixels) that can be used as a visual synopsis on our website.

I look forward to seeing the final revised version of your manuscript when it is ready. Please let me know if you have questions regarding the revision.

Yours sincerely,

Achim Breiling
Senior Editor
EMBO Reports

Referee #1:

The Authors have addressed all of my concerns with the original manuscript. For this reviewer the revised manuscript is ready for publication.

Referee #2

I am content with the answers and modifications the authors provided which further improved this very nice piece of work.

Referee #3:

The authors have addressed my comments adequately.

The authors have addressed all minor editorial requests.

2nd Revision - Editorial Decision **18th Mar 2022**

Prof. Yvonne Stahl
Heinrich-Heine University
Institute for Developmental Genetics
Universitätsstr. 1
Düsseldorf, NRW D-40225
Germany

Dear Prof. Stahl,

I am very pleased to accept your manuscript for publication in the next available issue of EMBO reports. Thank you for your contribution to our journal.

At the end of this email I include important information about how to proceed. Please ensure that you take the time to read the information and complete and return the necessary forms to allow us to publish your manuscript as quickly as possible.

As part of the EMBO publication's Transparent Editorial Process, EMBO reports publishes online a Review Process File to accompany accepted manuscripts. As you are aware, this File will be published in conjunction with your paper and will include the referee reports, your point-by-point response and all pertinent correspondence relating to the manuscript.

If you do NOT want this File to be published, please inform the editorial office within 2 days, if you have not done so already, otherwise the File will be published by default [contact: emboreports@embo.org]. If you do opt out, the Review Process File link will point to the following statement: "No Review Process File is available with this article, as the authors have chosen not to make the review process public in this case."

Should you be planning a Press Release on your article, please get in contact with emboreports@wiley.com as early as possible, in order to coordinate publication and release dates.

Thank you again for your contribution to EMBO reports and congratulations on a successful publication. Please consider us again in the future for your most exciting work.

Yours sincerely,

Achim Breiling
Editor
EMBO Reports

****

THINGS TO DO NOW:

Please note that you will be contacted by Wiley Author Services to complete licensing and payment information. The required 'Page Charges Authorization Form' is available here: https://www.embopress.org/pb-assets/embo-site/er_apc.pdf

You will receive proofs by e-mail approximately 2-3 weeks after all relevant files have been sent to our Production Office; you should return your corrections within 2 days of receiving the proofs.

Please inform us if there is likely to be any difficulty in reaching you at the above address at that time. Failure to meet our deadlines may result in a delay of publication, or publication without your corrections.

All further communications concerning your paper should quote reference number EMBOR-2021-54105V3 and be addressed to emboreports@wiley.com.

Should you be planning a Press Release on your article, please get in contact with emboreports@wiley.com as early as possible, in order to coordinate publication and release dates.

EMBO PRESS

YOU MUST COMPLETE ALL CELLS WITH A PINK BACKGROUND

PLEASE NOTE THAT THIS CHECKLIST WILL BE PUBLISHED ALONGSIDE YOUR PAPER

Corresponding Author Name: Yvonne Stahl
Journal Submitted to: EMBO Reports
Manuscript Number: EMBOR-2021-54105

USEFUL LINKS FOR COMPLETING THIS FORM

http://www.antibodypedia.com
http://1degreebio.org
http://www.equator-network.org/reporting-guidelines/improving-bioscience-research-repor
http://grants.nih.gov/grants/olaw/olaw.htm
http://www.mrc.ac.uk/Ourresearch/Ethicsresearchguidance/Useofanimals/index.htm
http://ClinicalTrials.gov
http://www.consort-statement.org
http://www.consort-statement.org/checklists/view/32-consort/66-title
http://www.equator-network.org/reporting-guidelines/reporting-recommendations-for-tumo
http://datadryad.org
http://figshare.com
http://www.ncbi.nlm.nih.gov/gap
http://www.ebi.ac.uk/ega
http://biomodels.net/
http://biomodels.net/miriam/
http://jjj.biochem.sun.ac.za
https://osp.od.nih.gov/biosafety-biosecurity-and-emerging-biotechnology/
http://www.selectagents.gov/

Reporting Checklist For Life Sciences Articles (Rev. June 2017)

This checklist is used to ensure good reporting standards and to improve the reproducibility of published results. These guidelines are consistent with the Principles and Guidelines for Reporting Preclinical Research issued by the NIH in 2014. Please follow the journal's authorship guidelines in preparing your manuscript.

A- Figures
1. Data
The data shown in figures should satisfy the following conditions:

- the data were obtained and processed according to the field's best practice and are presented to reflect the results of the experiments in an accurate and unbiased manner.
- figure panels include only data points, measurements or observations that can be compared to each other in a scientifically meaningful way.
- graphs include clearly labeled error bars for independent experiments and sample sizes. Unless justified, error bars should not be shown for technical replicates.
- if n< 5, the individual data points from each experiment should be plotted and any statistical test employed should be justified
- Source Data should be included to report the data underlying graphs. Please follow the guidelines set out in the author ship guidelines on Data Presentation.

2. Captions

Each figure caption should contain the following information, for each panel where they are relevant:

- a specification of the experimental system investigated (eg cell line, species name).
- the assay(s) and method(s) used to carry out the reported observations and measurements
- an explicit mention of the biological and chemical entity(ies) that are being measured.
- an explicit mention of the biological and chemical entity(ies) that are altered/varied/perturbed in a controlled manner.

- the exact sample size (n) for each experimental group/condition, given as a number, not a range;
- a description of the sample collection allowing the reader to understand whether the samples represent technical or biological replicates (including how many animals, litters, cultures, etc.).
- a statement of how many times the experiment shown was independently replicated in the laboratory.
- definitions of statistical methods and measures:
  - common tests, such as t-test (please specify whether paired vs. unpaired), simple χ^2 tests, Wilcoxon and Mann-Whitney tests, can be unambiguously identified by name only, but more complex techniques should be described in the methods section;
  - are tests one-sided or two-sided?
  - are there adjustments for multiple comparisons?
  - exact statistical test results, e.g., P values = x but not P values < x;
  - definition of 'center values' as median or average;
  - definition of error bars as s.d. or s.e.m.

Any descriptions too long for the figure legend should be included in the methods section and/or with the source data.

In the pink boxes below, please ensure that the answers to the following questions are reported in the manuscript itself. Every question should be answered. If the question is not relevant to your research, please write NA (non applicable). We encourage you to include a specific subsection in the methods section for statistics, reagents, animal models and human subjects.

B- Statistics and general methods

Please fill out these boxes (Do not worry if you cannot see all your text once you press return)

1.a. How was the sample size chosen to ensure adequate power to detect a pre-specified effect size?	Sample size was not chosen upfront
1.b. For animal studies, include a statement about sample size estimate even if no statistical methods were used.	n.a.
2. Describe inclusion/exclusion criteria if samples or animals were excluded from the analysis. Were the criteria pre-established?	n.a.
3. Were any steps taken to minimize the effects of subjective bias when allocating animals/samples to treatment (e.g. randomization procedure)? If yes, please describe.	no
For animal studies, include a statement about randomization even if no randomization was used.	n.a.
4.a. Were any steps taken to minimize the effects of subjective bias during group allocation or/and when assessing results (e.g. blinding of the investigator)? If yes please describe.	n.a.
4.b. For animal studies, include a statement about blinding even if no blinding was done	n.a.
5. For every figure, are statistical tests justified as appropriate?	yes
Do the data meet the assumptions of the tests (e.g., normal distribution)? Describe any methods used to assess it.	Data was tested for normal distribution by Kolmogorov-Smirnov testing. In case of normal distribution below 0.05 niveau, the data was subsequently analyzed by one-way ANOVA and post-hoc Holm-Sidak multiple comparisons test with alpha = 0.01 to identify statistically significant differences. In case of not normally distributed data, the non-parametric Kruskal-Wallis test and posthoc Dunns multiple comparisons test with alpha = 0.01 was used to identify statistically significant differences.
Is there an estimate of variation within each group of data?	see above

Is the variance similar between the groups that are being statistically compared?	see above

C- Reagents

6. To show that antibodies were profiled for use in the system under study (assay and species), provide a citation, catalog number and/or clone number, supplementary information or reference to an antibody validation profile. e.g., Antibodypedia (see link list at top right), 1DegreeBio (see link list at top right).	n.a.
7. Identify the source of cell lines and report if they were recently authenticated (e.g., by STR profiling) and tested for mycoplasma contamination.	n.a.

* for all hyperlinks, please see the table at the top right of the document

D- Animal Models

8. Report species, strain, gender, age of animals and genetic modification status where applicable. Please detail housing and husbandry conditions and the source of animals.	n.a.
9. For experiments involving live vertebrates, include a statement of compliance with ethical regulations and identify the committee(s) approving the experiments.	n.a.
10. We recommend consulting the ARRIVE guidelines (see link list at top right) (PLoS Biol. 8(6), e1000412, 2010) to ensure that other relevant aspects of animal studies are adequately reported. See author guidelines, under 'Reporting Guidelines'. See also: NIH (see link list at top right) and MRC (see link list at top right) recommendations. Please confirm compliance.	n.a.

E- Human Subjects

11. Identify the committee(s) approving the study protocol.	n.a.
12. Include a statement confirming that informed consent was obtained from all subjects and that the experiments conformed to the principles set out in the WMA Declaration of Helsinki and the Department of Health and Human Services Belmont Report.	n.a.
13. For publication of patient photos, include a statement confirming that consent to publish was obtained.	n.a.
14. Report any restrictions on the availability (and/or on the use) of human data or samples.	n.a.
15. Report the clinical trial registration number (at ClinicalTrials.gov or equivalent), where applicable.	n.a.
16. For phase II and III randomized controlled trials, please refer to the CONSORT flow diagram (see link list at top right) and submit the CONSORT checklist (see link list at top right) with your submission. See author guidelines, under 'Reporting Guidelines'. Please confirm you have submitted this list.	n.a.
17. For tumor marker prognostic studies, we recommend that you follow the REMARK reporting guidelines (see link list at top right). See author guidelines, under 'Reporting Guidelines'. Please confirm you have followed these guidelines.	n.a.

F- Data Accessibility

18: Provide a "Data Availability" section at the end of the Materials & Methods, listing the accession codes for data generated in this study and deposited in a public database (e.g. RNA-Seq data: Gene Expression Omnibus GSE39462, Proteomics data: PRIDE PXD000208 etc.) Please refer to our author guidelines for 'Data Deposition'.<br><br>Data deposition in a public repository is mandatory for:<br>a. Protein, DNA and RNA sequences<br>b. Macromolecular structures<br>c. Crystallographic data for small molecules<br>d. Functional genomics data<br>e. Proteomics and molecular interactions	Data availability section is provided, but no primary datasets have been generated and deposited.
19. Deposition is strongly recommended for any datasets that are central and integral to the study; please consider the journal's data policy. If no structured public repository exists for a given data type, we encourage the provision of datasets in the manuscript as a Supplementary Document (see author guidelines under 'Expanded View' or in unstructured repositories such as Dryad (see link list at top right) or Figshare (see link list at top right).	Source datasets are provided as supplementary documents and EV tables
20. Access to human clinical and genomic datasets should be provided with as few restrictions as possible while respecting ethical obligations to the patients and relevant medical and legal issues. If practically possible and compatible with the individual consent agreement used in the study, such data should be deposited in one of the major public access-controlled repositories such as dbGAP (see link list at top right) or EGA (see link list at top right).	n.a.
21. Computational models that are central and integral to a study should be shared without restrictions and provided in a machine-readable form. The relevant accession numbers or links should be provided. When possible, standardized format (SBML, CellML) should be used instead of scripts (e.g. MATLAB). Authors are strongly encouraged to follow the MIRIAM guidelines (see link list at top right) and deposit their model in a public database such as Biomodels (see link list at top right) or JWS Online (see link list at top right). If computer source code is provided with the paper, it should be deposited in a public repository or included in supplementary information.	n.a.

G- Dual use research of concern

22. Could your study fall under dual use research restrictions? Please check biosecurity documents (see link list at top right) and list of select agents and toxins (APHIS/CDC) (see link list at top right). According to our biosecurity guidelines, provide a statement only if it could.	no
